# Estimates of the global burden of cervical cancer associated with HIV

**DOI:** 10.1016/S2214-109X(20)30459-9

**Published:** 2020-11-16

**Authors:** Dominik Stelzle, Luana F Tanaka, Kuan Ken Lee, Ahmadaye Ibrahim Khalil, Iacopo Baussano, Anoop S V Shah, David A McAllister, Sami L Gottlieb, Stefanie J Klug, Andrea S Winkler, Freddie Bray, Rachel Baggaley, Gary M Clifford, Nathalie Broutet, Shona Dalal

**Affiliations:** a Center for Global Health, Department of Neurology, Faculty of Medicine, Technical University of Munich, Munich, Germany; b Chair of Epidemiology, Department of Sport and Health Sciences, Technical University of Munich, Munich, Germany; c British Heart Foundation Centre for Cardiovascular Science, University of Edinburgh, Edinburgh, UK; d International Agency for Research on Cancer, Lyon, France; e Department of Non-Communicable Disease Epidemiology, London School of Hygiene & Tropical Medicine, London, UK; f Institute of Health and Wellbeing, University of Glasgow, Glasgow, UK; g Department of Sexual and Reproductive Health and Research, World Health Organization, Geneva, Switzerland; h Department of Global HIV, Hepatitis and STIs Programmes, World Health Organization, Geneva, Switzerland; i Centre for Global Health, Institute of Health and Society, Faculty of Medicine, University of Oslo, Oslo, Norway

## Abstract

**Background:**

HIV enhances human papillomavirus (HPV)-induced carcinogenesis. However, the contribution of HIV to cervical cancer burden at a population level has not been quantified. We aimed to investigate cervical cancer risk among women living with HIV and to estimate the global cervical cancer burden associated with HIV.

**Methods:**

We did a systematic literature search and meta-analysis of five databases (PubMed, Embase, Global Health [CABI.org], Web of Science, and Global Index Medicus) to identify studies analysing the association between HIV infection and cervical cancer. We estimated the pooled risk of cervical cancer among women living with HIV across four continents (Africa, Asia, Europe, and North America). The risk ratio (RR) was combined with country-specific UNAIDS estimates of HIV prevalence and GLOBOCAN 2018 estimates of cervical cancer to calculate the proportion of women living with HIV among women with cervical cancer and population attributable fractions and age-standardised incidence rates (ASIRs) of HIV-attributable cervical cancer.

**Findings:**

24 studies met our inclusion criteria, which included 236 127 women living with HIV. The pooled risk of cervical cancer was increased in women living with HIV (RR 6·07, 95% CI 4·40–8·37). Globally, 5·8% (95% CI 4·6–7·3) of new cervical cancer cases in 2018 (33 000 new cases, 95% CI 26 000–42 000) were diagnosed in women living with HIV and 4·9% (95% CI 3·6–6·4) were attributable to HIV infection (28 000 new cases, 20 000–36 000). The most affected regions were southern Africa and eastern Africa. In southern Africa, 63·8% (95% CI 58·9–68·1) of women with cervical cancer (9200 new cases, 95% CI 8500–9800) were living with HIV, as were 27·4% (23·7–31·7) of women in eastern Africa (14 000 new cases, 12 000–17 000). ASIRs of HIV-attributable cervical cancer were more than 20 per 100 000 in six countries, all in southern Africa and eastern Africa.

**Interpretation:**

Women living with HIV have a significantly increased risk of cervical cancer. HPV vaccination and cervical cancer screening for women living with HIV are especially important for countries in southern Africa and eastern Africa, where a substantial HIV-attributable cervical cancer burden has added to the existing cervical cancer burden.

**Funding:**

WHO, US Agency for International Development, and US President's Emergency Plan for AIDS Relief.

## Introduction

Although cervical cancer is one of the most preventable and treatable malignant diseases, it is the fourth most commonly detected cancer in women worldwide, with more than half a million new cases and 311 365 deaths in 2018.^1^ Infection with high-risk human papillomavirus (HPV; types 16, 18, 31, 33, 35, 39, 45, 51, 52, 56, 58, and 59) alone does not cause cervical cancer.^2^ Other known risk factors include smoking, increased parity, and infection with HIV.

Geographical disparities in the cervical cancer disease burden are stark and reflect the availability, coverage, and quality of preventive strategies and the prevalence of risk factors. Nearly nine in ten women who die from cervical cancer live in low-income and middle-income countries (LMICs).^1^ Inequalities are widening: observed incidence rates are declining most rapidly in high-income countries, with some countries moving towards cervical cancer elimination in the coming decades.^3^ By contrast, rates are increasing in some sub-Saharan African settings^1^, ^4^ and have increased or remained relatively stable at high levels in several eastern European and western Asian countries.^1^, ^4^, ^5^

Cervical cancer is the most frequently detected cancer in women living with HIV and is classified as an AIDS-defining illness.^6^ With the advent of antiretroviral therapy (ART), AIDS-related mortality has declined considerably, and life expectancy of people living with HIV has increased nearly to the same level as those without HIV.^7^ As a result, the number of adult women living with HIV has increased from an estimated 3·3 million in 1990 to 18·8 million in 2018; 60% of these women live in eastern and southern Africa.^8^

**Research in context**

**Evidence before this study**

A meta-analysis published in 2007 reported an increased risk of cervical cancer among women living with HIV (risk ratio 5·8, 95% CI 3·0–11·3), based on data from six high-income countries. No subsequent studies have been done to update this estimate, and no studies have extended results to low-income and middle-income countries.

**Added value of this study**

We present a pooled estimate of cervical cancer risk derived from 24 individual studies, which included 236 127 women living with HIV from four continents (Africa, Asia, Europe, and North America). We also provide global estimates of the proportion of women with cervical cancer living with HIV alongside the population attributable fraction and incidence of cervical cancer attributable to HIV infection.

**Implications of all the available evidence**

Women living with HIV have a substantially increased risk for cervical cancer when compared with women without HIV infection. Worldwide, roughly 6% of women with cervical cancer are living with HIV and just under 5% of all cases of cervical cancer are attributable to HIV. However, these proportions vary widely by region; 85% of women with cervical cancer and HIV live in sub-Saharan Africa, underscoring the major contribution of HIV to cervical cancer burden in the region. In countries with a high burden of both cervical cancer and HIV, it is vital to integrate HIV and cervical cancer care and vaccinate girls against human papillomavirus to secure long-term declines in the future disease burden.

Although high-burden settings for cervical cancer and HIV overlap, the extent of the contribution of HIV to the burden of cervical cancer and the proportion of cervical cancer cases due to co-infection with HIV have yet to be quantified. In this meta-analysis and modelling study, we aimed to provide a pooled estimate of the relative risk for cervical cancer among women living with HIV and present the estimated number of cases of cervical cancer among women living with HIV and attributable to HIV, based on UNAIDS HIV prevalence and GLOBOCAN 2018 cancer estimates for 2018.

## Methods

### Identification and selection of relevant studies

We systematically searched five databases (PubMed, Embase, Global Health [CABI.org], Web of Science, and Global Index Medicus) to identify studies analysing the association between HIV infection and cervical cancer. The detailed search strategy can be found in the appendix (pp 3–6). In short, we used a WHO validated search strategy for “HIV” and “AIDS” and combined these terms with “cervical cancer” and standard WHO epidemiological search terms (eg, “incidence” and “prevalence”). The search was done on April 29, 2019. We did not restrict our search by language or year of publication. We included published epidemiological studies (cohort, case-control, and registry linkage studies) reporting the risk of cervical cancer (International Classification of Diseases 10th revision [ICD-10] code C53) as either a risk ratio (RR), odds ratio (OR), hazard ratio (HR), incidence rate ratio (IRR), or standardised incidence ratio (SIR) among women living with HIV compared with either women without HIV infection or the general female reference population. Our exclusion criteria were HIV status based on self-report and studies with fewer than four cases of cervical cancer.

### Data extraction and risk of bias assessment

The published literature was screened for inclusion by two of us (DS and LFT) independently. These same reviewers extracted data from the included studies. Discrepancies were resolved by consensus between the two reviewers. In quantitative analyses, only one study per cohort was included to avoid double inclusion of patients. We selected the study with either the longest follow-up time or the largest sample size. We listed all studies that met inclusion criteria and studies used for the calculation of the pooled RR (appendix pp 7–18); for those excluded, we reported the main reason. The flowchart for study selection and the MOOSE checklist are in the appendix (pp 19–21). Risk of bias was assessed based on the Newcastle-Ottawa scale, a tool used to appraise observational studies.^9^ We classified studies as having a high risk of bias if scores were lower than eight stars. All other studies were considered as having low or moderate risk of bias.

### Estimates of HIV prevalence and cervical cancer incidence for the year 2018

For the statistical modelling, HIV prevalence estimates for adult women (aged ≥15 years) for the year 2018 were obtained from UNAIDS.^8^ HIV prevalence estimates were available for 170 of 194 UN member states. Country-specific estimates (total number of cases and incidence rates per 100 000) for invasive cervical cancer (ICD-10 code C53) provided by the International Agency for Research on Cancer (IARC) were available for 185 countries.^1^, ^10^ Both data sources combined were available for 170 countries. The 15 countries and territories for which no HIV estimates were available accounted for fewer than 1000 cases of cervical cancer in 2018 (appendix p 64) and did not affect either the overall or regional estimates in our analysis.

### Statistical analysis

A detailed description of our methods can be found elsewhere (appendix pp 23–25).^11^ We considered RR, OR, and SIR to be equivalent in our pooled estimates. In summary, estimates for the overall RR for cervical cancer and those stratified by subgroups were calculated using random-effects models, based on the risk estimate and its SE; weighting was based on an inverse variance method. Heterogeneity across studies was analysed by the *I*^2^ index.

Using a simulation-based approach, we propagated uncertainty from estimates of the pooled RR and HIV prevalence to calculate the proportion of women with cervical cancer living with HIV for each country (equation 1) and the population attributable fraction (PAF) for each country (equation 2). Cause-specific risk estimates from random effects meta-analysis were represented as log normal (ln) distributions, from which 10 000 samples were obtained using R (version 3.6.1). We represented HIV prevalence as a beta distribution and obtained 10 000 samples for each country. We subsequently did the calculation shown for each of the 10 000 samples to derive the country-specific population attributable fraction (PAF_c_). HIV prevalence is country specific (HIV prevalence_c_) and the pooled RR is universal.

(1) $$\mathrm{Pr}oportionofwomenwithcervicalcancerlivingwithHIV=\frac{HIVprevalence_{c}\times pooledRR}{\left(1-HIVprevalence_{c})+(HIVprevalence_{c}\times pooledRR\right)}$$

(2) $$Populationattributablefraction(PAF_{c})=\frac{HIVprevalence_{c}\times (pooledRR-1)}{1+HIVprevalence_{c}\times (pooledRR-1)}$$

Multiplying the number of incident cervical cancer cases by the proportion of women with cervical cancer living with HIV and by the PAF yielded estimates for both the total number of new cases of cervical cancer among women living with HIV and the total number of cases of cervical cancer attributable to HIV for the year 2018, respectively. Multiplying the incidence of cervical cancer per 100 000 by the PAF yielded the incidence rate for cervical cancer attributable to HIV (and the incidence rate of cervical cancer not attributable to HIV). We ran these analyses for all countries and integrated the results into regional and global estimates. We report the results by country, WHO region, UNAIDS region, and UN Africa subregion (appendix pp 49–60). World maps were created to display these estimates by country, divided into five levels, with the darkest shades representing the highest estimates. Statistical analyses were done in R (version 3.6.1) using the packages *metafor* (version 2.4-0) and *rworldmap* (version 1.3-6).

The protocol of this systematic review was published on PROSPERO, number CRD42019139813.

### Role of the funding source

SLG, RB, NB, and SD are staff members at WHO, and AIK, IB, FB, and GMC are employed at IARC. The external funders of the study (US Agency for International Development [USAID] and the US President's Emergency Plan for AIDS Relief [PEPFAR]) had no role in study design, data collection, data analysis, data interpretation, or writing of the report. DS and SD had full access to all data in the study. SD had final responsibility for the decision to submit for publication.

## Results

24 studies met our inclusion criteria and provided 31 risk estimates, which were used to calculate the overall estimate (appendix pp 7–18, 21). Overall, 236 127 women living with HIV were included in these studies and 2138 cases of cervical cancer were reported (among women living with HIV and those without HIV infection). At study entry, median age was 38 years (IQR 34–42) and median ART coverage was 40% (IQR 25–49).

The included studies were done between 1981 and 2016 and were located in 17 countries (including one territory) located on four continents: Africa (Benin, Côte d'Ivoire, Nigeria, Rwanda, Senegal, Tanzania, Togo, and Uganda), Asia (China, India, and Taiwan), Europe (France, Germany, Italy, and Switzerland), and North America (Canada and the USA). Most were registry linkage studies (n=11), followed by cohort studies (n=7) and case-control studies (n=6). 19 studies (providing 26 risk estimates) were considered to have a high risk of bias based on the Newcastle-Ottawa scale.

**Figure 1:**
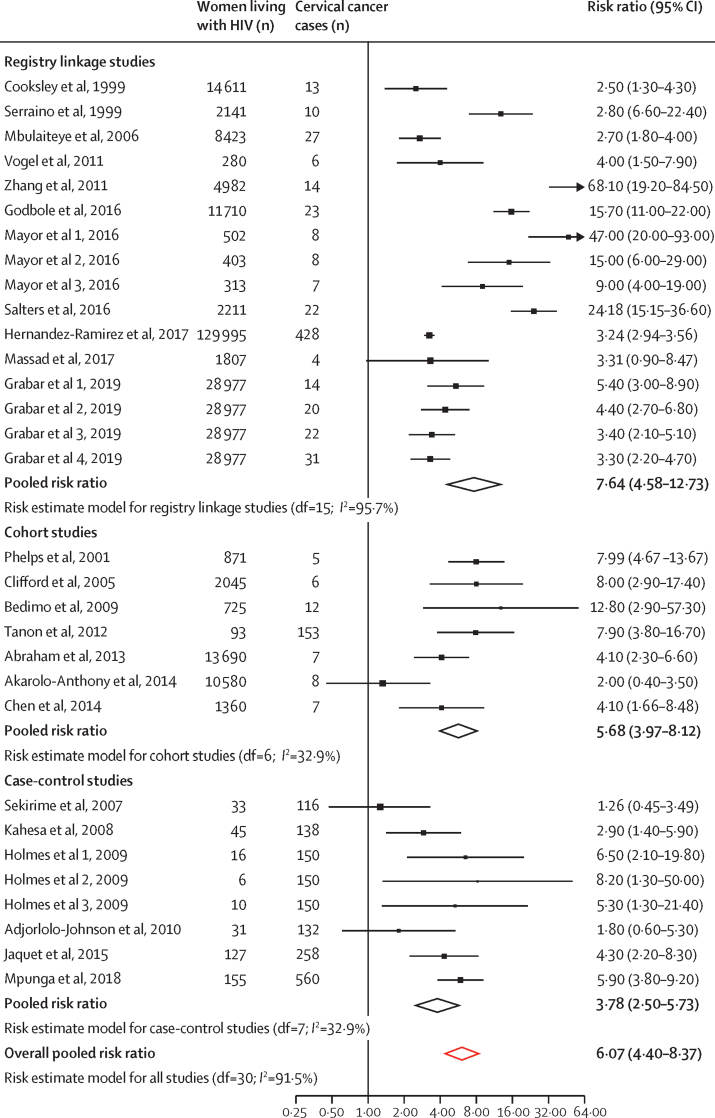
Risk of developing cervical cancer among women living with HIV, by type of study

The overall pooled risk of cervical cancer among women living with HIV was increased (RR 6·07, 95% CI 4·40–8·37), based on highly heterogeneous data (*I*^2^=91·5%; figure 1; appendix p 22). Risk estimates ranged from 1·26 to 68·10, and only four estimates were not higher than unity in women living with HIV.

**Figure 2:**
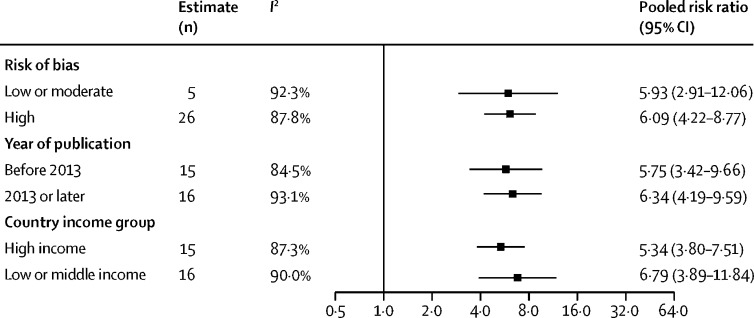
Risk of developing cervical cancer among women living with HIV, by subgroups Meta-regression to assess interaction was not significant for risk of bias (p=0·96), year of publication (p=0·77), or country income group (p=0·46).

In subgroup analyses by study design, registry linkage studies yielded a more than seven-fold risk for developing cervical cancer among women living with HIV compared with the general population (pooled RR 7·64, 95% CI 4·58–12·73), and in cohort studies the pooled RR was more than five-fold (5·68, 3·97–8·12; figure 1). For case-control studies, the corresponding risk was more than three-fold (pooled RR 3·78, 95% CI 2·50–5·73). The CIs of the subgroups overlapped. Figure 2 shows no substantial differences in pooled RRs for studies with different degrees of bias (high *vs* low or moderate), year of publication (studies published before 2013 *vs* those published in 2013 or later), country income group (studies done in LMICs *vs* high-income countries), or continent (appendix p 48). Since cervical cancer can be considered a rare outcome, corresponding measures of association (SIR, OR, and RR) should be similar in magnitude, and no differences were noted between SIRs, ORs, or RRs (appendix p 48). A universal RR was, therefore, used to calculate estimates of cervical cancer burden associated with HIV.

Globally, an estimated 33 000 (95% CI 26 000–42 000) new cases of cervical cancer occurred among women living with HIV, corresponding to 5·8% (95% CI 4·6–7·3) of 569 847 cases of cervical cancer in the year 2018 (table). Particularly affected was the African region, specifically the UN Africa subregions of southern Africa (63·8%, 95% CI 58·9–68·1), with 9200 new cases (95% CI 8500–9800), and eastern Africa (27·4%, 23·7–31·7), with 14 000 new cases (12 000–17 000). In combination, these subregions contributed 70% of this double burden of HIV and cervical cancer (table). By contrast, the proportion of new cervical cancer cases was low in the Western Pacific region (0·4%, 95% CI 0·3–0·6), the Eastern Mediterranean region (0·5%, 0·3–0·8), and the South-East Asia region (1·4%, 1·0–2·1; table).

**Table:** Estimated proportion of cervical cancer associated with HIV and population attributable fraction in 2018, by WHO regions and UN Africa subregions

		**Number (95% CI) of new cervical cancer cases, 2018**	**HIV prevalence (95% CI) in females aged ≥15 years**	**Proportion (95% CI) of new cervical cancer patients living with HIV**	**Number (95% CI) of new cervical cancer patients living with HIV, 2018**	**Population attributable fraction (95% CI) for HIV**	**Number (95% CI) of cervical cancer cases attributable to HIV, 2018**
Global		569 847 (545 771–594 985)	0·67% (0·59–0·78)	5·8% (4·6–7·3)	33 000 (26 000–42 000)	4·9% (3·6–6·4)	28 000 (20 000–36 000)
WHO region							
	South-East Asia	158 692 (151 987–165 692)	0·21% (0·18–0·27)	1·4% (1·0–2·1)	2300 (1500–3300)	1·2% (0·8–1·8)	1900 (1200–2900)
	Western Pacific	142 251 (136 241–148 526)	0·07% (0·06–0·08)	0·4% (0·3–0·6)	590 (420–820)	0·3% (0·2–0·5)	490 (330–720)
	African	112 036 (107 302–116 978)	4·74% (4·14–5·41)	25·1% (20·1–30·7)	28 000 (23 000–34 000)	21·0% (15·6–26·8)	24 000 (18 000–30 000)
	Americas	71 689 (68 660–74 851)	0·25% (0·21–0·30)	1·7% (1·1–2·4)	1200 (810–1700)	1·4% (0·9–2·1)	980 (630–1500)
	European	69 114 (66 194–72 163)	0·20% (0·18–0·22)	1·5% (1·1–2·1)	1000 (730–1400)	1·2% (0·8–1·8)	860 (570–1200)
	Eastern Mediterranean	15 903 (15 231–16 605)	0·06% (0·04–0·09)	0·5% (0·3–0·8)	80 (50–120)	0·4% (0·3–0·6)	60 (40–100)
UN Africa subregion							
	Eastern Africa	52 500 (42 840–65 052)	2·96% (2·56–3·42)	27·4% (23·7–31·7)	14 000 (12 000–17 000)	22·9% (19·8–26·4)	12 000 (10 000–14 000)
	Central Africa	12 635 (9644–16 573)	1·64% (1·38–1·92)	12·3% (10·3–13·4)	1600 (1300–1800)	10·2% (8·6–12·0)	1300 (1100–1500)
	North Africa	7639 (6323–9325)	0·07% (0·05–0·11)	0·5% (0·3–0·8)	40 (30–60)	0·4% (0·3–0·6)	30 (20–50)
	Southern Africa	14 409 (13 371–15 564)	29·83% (27·54–31·82)	63·8% (58·9–68·1)	9200 (8500–9800)	53·2% (49·1–56·8)	7700 (7100–8200)
	West Africa	31 939 (24 702–45 586)	1·39% (1·09-1·78)	9·5% (7·5–12·2)	3000 (2400–3900)	7·9% (6·2–10·1)	2500 (2000–3200)

Eswatini had the largest proportion of women with cervical cancer living with HIV (75·0%, 95% CI 68·2–80·8), followed by Lesotho (69·3%, 61·7–76·0), Botswana (66·5%, 58·6–73·5), South Africa (63·4%, 55·2–70·7), and Zimbabwe (52·2%, 43·5–60·9; appendix pp 37–43). By contrast, this proportion was less than 5% in 122 countries globally. In absolute terms, most women with cervical cancer living with HIV were from South Africa (8220 cases, 95% CI 7090–9320), followed by Tanzania (2610, 1900–3520), Mozambique (2150, 1670–2690), Uganda (2050, 1560–2640), and Malawi (1790, 1370–2300; appendix pp 37–43).

In 2018, an estimated 4·9% (95% CI 3·6–6·4) of all cases of cervical cancer globally (28 000 cases, 95% CI 20 000–36 000) were attributable to HIV (table), ranging from less than 1% in the Western Pacific region (0·3%, 95% CI 0·2–0·5; 490 cases, 95% CI 330–720), and the Eastern Mediterranean region (0·4%, 0·3–0·6; 60 cases, 40–100), to more than 20% in the African region (21·0%, 15·6–26·8; 24 000 cases, 18 000–30 000; table). Cases of cervical cancer in the African region alone accounted for 85% of cases attributable to HIV.

**Figure 3:**
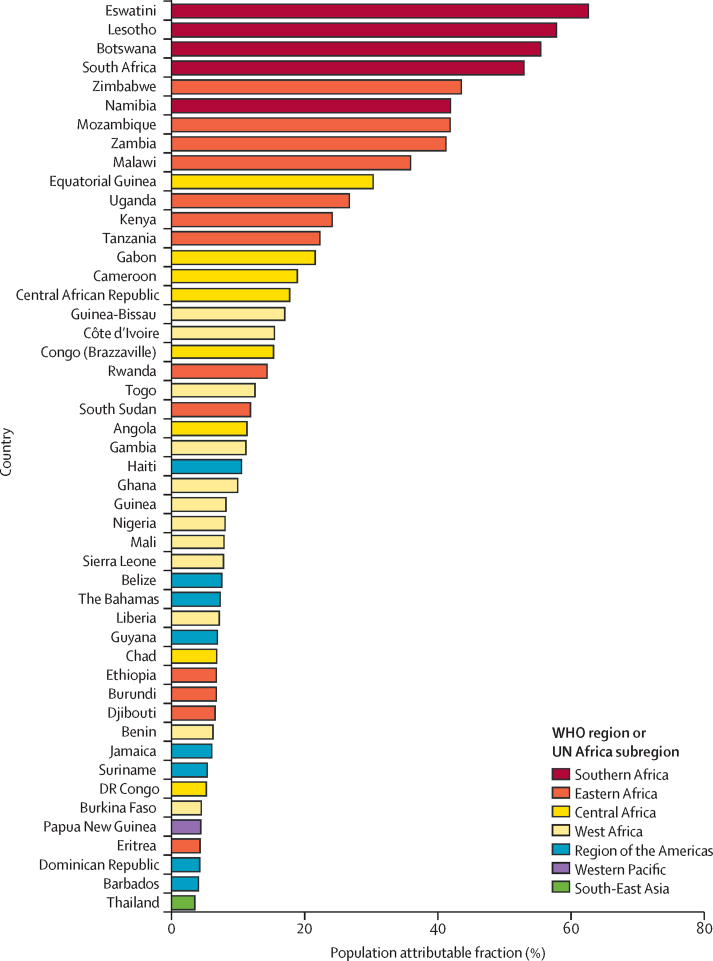
Estimated population attributable fraction for cervical cancer and HIV in the 50 highest ranked countries Estimates are ranked from highest to lowest proportion. The figure shows data for 48 countries; data for Turkmenistan (European; 5–9·99%) and Trinidad and Tobago (Americas; <5%) are not shown because HIV estimates are not published by UNAIDS.

**Figure 4:**
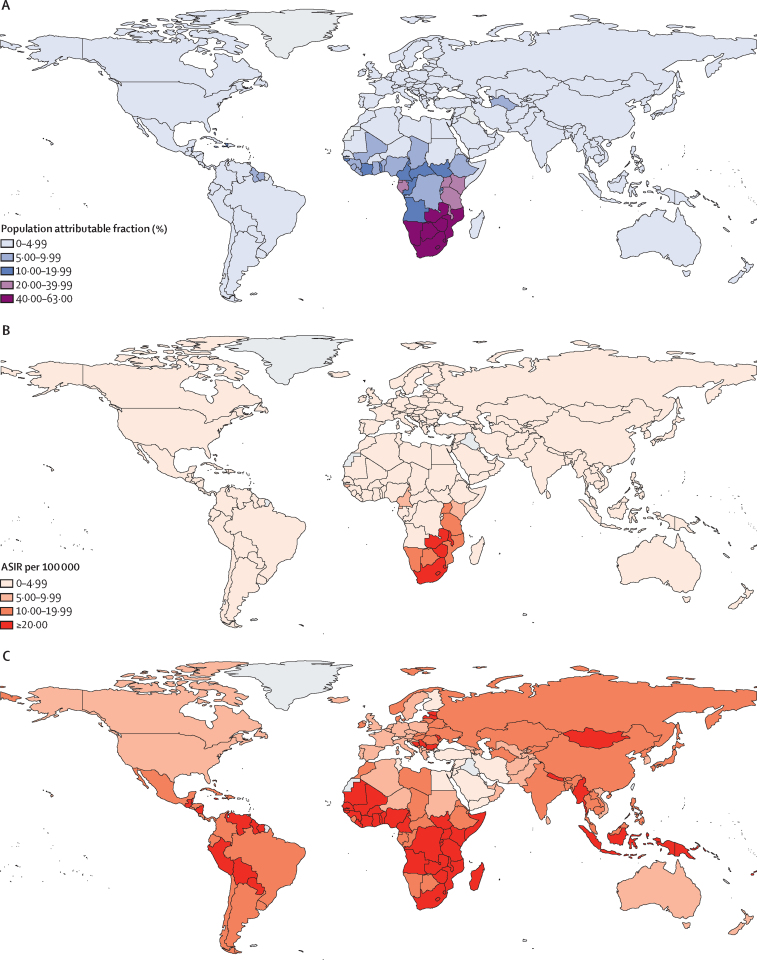
Maps showing cases of cervical cancer attributable, or not, to HIV (A) Population attributable fraction of women with cervical cancer living with HIV in 2018. (B) ASIR attributable to HIV (per 100 000) in 2018. (C) ASIR not attributable to HIV (per 100 000) in 2018. ASIR=age-standardised incidence rate.

Of 50 countries with the highest-ranking PAFs (appendix pp 37–43), 38 were in the African region, nine were in the Region of the Americas, and one each was in the Western Pacific, South-East Asia, and European regions (figure 3). The highest PAFs were seen for Eswatini (62·6%, 95% CI 52·8–70·8), Lesotho (57·9%, 95% CI 47·7–66·6), Botswana (55·5%, 45·4–64·4), South Africa (52·9%, 42·8–61·9), and Zimbabwe (43·6%, 33·6–53·2). Conversely, in 127 countries, PAFs were lower than 5% (figure 4A; appendix pp 37–43). Additional data, maps, and figures by WHO and UNAIDS regions are provided in the appendix (pp 27–36).

Age-standardised incidence rates (ASIRs) of cervical cancer attributable to HIV were highest in the southern and eastern Africa UN subregions (figure 4B). HIV-attributable ASIRs were 10–20 per 100 000 in Botswana, Mozambique, Namibia, Tanzania, and Uganda and were higher than 20 per 100 000 in Eswatini, Lesotho, Malawi, South Africa, Zambia, and Zimbabwe. ASIRs of cervical cancers not attributable to HIV were higher than 10 per 100 000 in all countries in sub-Saharan Africa and were higher than 20 per 100 000 in most countries in sub-Saharan Africa (figure 4C).

## Discussion

Our meta-analysis of 24 studies shows that women living with HIV have a six-fold higher risk of developing cervical cancer relative to their counterparts without HIV. Globally, an estimated 6% of new cervical cancer cases in 2018 were diagnosed among women living with HIV and 5% of all cases were attributable to HIV infection. Due to the substantial variation in HIV prevalence worldwide, the proportion of women living with HIV among patients with cervical cancer ranged from less than 5% in 122 countries to 40% or more in nine countries. In both relative and absolute terms, the southern and eastern Africa UN subregions bear the largest share of this burden, with a respective 64% and 27% of women with cervical cancer living with HIV in these regions. According to our estimates, six countries—namely Malawi, Mozambique, South Africa, Tanzania, Uganda, and Zimbabwe—accounted for about half of all women living with HIV who developed cervical cancer in 2018. Although HIV is an important contributor to the burden of cervical cancer, other factors (particularly screening coverage and performance) also affect cervical cancer incidence rates, as shown by the variability in incidence rates overall.

The increased risk for cervical cancer in women living with HIV is a result of a combination of factors.^2^ Although HPV infection is the necessary underlying cause of all cervical cancers, women living with HIV are more likely to acquire an HPV infection—and less likely to clear infection—than are women without HIV, both factors contributing to higher rates of persistent HPV infection in this population.^12^, ^13^ Furthermore, HIV has an indirect role in oncogenesis, mainly via immune suppression, enhancing the effects of high-risk HPV.^14^ This role is highlighted by evidence that cervical cancer is associated with a lower CD4 cell count and no ART among women living with HIV.^15^ Due to these synergistic actions, in countries with a high prevalence of both HPV and HIV infections, the cervical cancer burden is substantially raised, particularly among women not on ART.^15^ Not only does HIV increase the risk for cervical cancer but also it increases rates of recurrence after treatment of precancer^16^ and reduces life expectancy.^17^ In a prospective study from Botswana,^17^ women living with HIV with cervical cancer were more likely to die from this malignant disease than were women without HIV infection, even after adjusting for disease stage and other relevant variables. Survival disparities were even more pronounced for early-stage disease.^17^

Cervical cancer screening followed by adequate management and HPV vaccination are two prevention tools recommended by WHO that are highly effective, with cost-effectiveness of US$100 or less per disability-adjusted life-year averted in LMICs. This cost places these interventions among global so-called best buys for non-communicable disease prevention.^18^ Globally, only a third of girls live in countries that have introduced the HPV vaccine, with LMICs the least likely to offer the vaccine.^19^ Increasing HPV vaccination coverage in countries with a high burden of cervical cancer attributable to HIV is a critical priority. Furthermore, some areas with the highest cervical cancer burden, including among women living with HIV, are those where population-wide screening coverage is low.^20^ Of the ten countries with the highest absolute burden of cervical cancer associated with HIV, seven had rolled out national HPV immunisation programmes, with full-dose coverage ranging from 25% to 80% by 2019.^20^, ^21^ In the same year, only four countries had ongoing population-based screening, but all covered less than 70% of the target population.^20^ Efforts by PEPFAR have led to initiation of cervical cancer screening for women living with HIV in some countries.^22^

In the short-term, screening is the main strategy to reduce cervical cancer incidence, by detecting precancerous lesions that can be treated before evolving to cancer. Currently, screening strategies recommended by WHO comprise visual inspection with acetic acid, cytology (conventional or liquid-based), and HPV DNA testing.^23^ HPV testing shows superior performance when compared with cytology or visual inspection with acetic acid. Effectiveness at the population level is affected not only by the accuracy of the screening method but also by its coverage and by treatment uptake.

Women living with HIV with access to care have clinical appointments at least every 6 months, which provides an opportunity for delivery of cervical cancer screening and treatment interventions, alongside appropriate follow-up. Methods to reach women who would not otherwise participate in screening could greatly improve participation. Self-sampling of cervical specimens has shown promising results and might be applicable in some settings.^24^ Our findings suggest that in settings with high HIV prevalence, integration of cervical cancer screening, treatment, and referral systems within HIV services could have a positive prevention effect on a large proportion of cervical cancers.^25^ Due to the increased risk for cervical cancer, specific guidelines for women living with HIV have been issued by WHO^26^ and are being updated. More data are needed on the relative performance of different implementation approaches, particularly in LMICs.

Over the longer term, HPV vaccination has a key role in the prospect of eliminating cervical cancer. The first publicly funded national HPV vaccination programmes were implemented in 2006 and by October, 2020, 57% of countries worldwide had introduced HPV vaccination.^19^ Regions that were the first to implement HPV vaccination programmes have already reported benefits, which include decreased prevalence of HPV types 16 and 18 infection and declines in the incidence of genital warts and of precancerous lesions among young women.^27^ A reduction in the incidence of cervical cancer will take longer to be detectable and its extent will be largely dependent on vaccination uptake.

WHO recommended that HPV vaccination strategies target young girls aged 9–14 years, before sexual debut, to ensure the highest level of protection.^28^ However, inequalities in access are striking, and only a third of girls aged 10 years were living in countries that had implemented HPV vaccination by 2019.^29^ Therefore, many girls living in countries with a high cervical cancer burden attributable to HIV remain unprotected against HPV infection. Expansion of HPV vaccination to areas with high HIV prevalence is especially important to achieve long-term reductions in the overall cervical cancer burden in those settings, as future birth cohorts might continue to be at high risk of HIV infection.

Trials of HPV vaccination in people living with HIV have shown its safety and indicate that the vaccine is immunogenic in this population.^28^, ^30^, ^31^ The public health benefit of vaccinating older adolescents and young women living with HIV, in view of their higher risk for cervical cancer, needs to be investigated further, since most new HIV infections are acquired sexually and, therefore, exposure to HPV infection might have already occurred.

The fraction and incidence of cervical cancer attributable to HIV offers a picture of how the burden of cervical cancer would be affected by removal of HIV as a risk factor in an ideal scenario. This insight clearly brings into focus the importance of HIV prevention measures on the future burden of cervical cancer in sub-Saharan Africa. Early diagnosis of HIV infection and timely initiation of ART might also contribute to reducing the cervical cancer burden among women living with HIV. In a meta-analysis,^15^ ART (*vs* no ART) was associated with lower prevalence of high-risk HPV and significantly lowered the risk of cervical cancer among women living with HIV.^32^

The data presented in this study have limitations, arising from two main sources. The first limitation of the data is that, although we calculated a pooled overall RR estimate, statistical heterogeneity of the included studies was considerable. Nonetheless, we decided to pool the data because part of this heterogeneity could be accounted for by differences in access to early diagnosis and ART and by the background cervical cancer risk in the studied populations, including the availability and uptake of cervical cancer screening. Moreover, we did not find substantial regional variations, differences between risk measures (RR, OR, or SIR), or changes in degrees of bias that would justify stratifying the RR for our estimates. Furthermore, 11 of the 24 included studies were registry linkage studies, providing SIRs as a measure of risk for which the comparison group was the general population (including women living with HIV). This could have led to an underestimation of risk in a high HIV prevalence setting, where women living with HIV would contribute to a considerable share of cases in the general population.^33^ With the exception of Uganda, regions where registry linkage studies were done have a low HIV prevalence. Hence, it is unlikely that the SIRs were substantially underestimated due to use of the general population as a comparison group. On the other hand, most registry linkage studies were done in high-income areas where population-wide screening for cervical cancer is in place and women living with HIV generally have improved access to screening, which might have attenuated the SIRs. Due to scant information on exposures available in registry linkage studies, most of the SIR estimates were solely adjusted by age. Therefore, other relevant risk factors for cervical cancer (eg, smoking, parity, and oral contraceptive use) were not considered, possibly resulting in an overestimation of risk. This overestimation is apparent in the subgroup analyses, in which registry linkage studies tended to provide higher estimates than did cohort studies and other study designs, after adjusting for other variables. Since HIV and HPV are strongly associated due to their common transmission route (sexual contact), there is a tendency for PAFs to be overestimated, particularly in countries with the highest prevalence of HIV infection. Nevertheless, without suitable data to disentangle this interaction, the provided estimates are, currently, the best possible approximation of the excess cervical cancer burden attributable to HIV. Furthermore, estimates of the proportion of cervical cancer cases diagnosed among women living with HIV are unaffected by this methodological consideration.

The second limitation of the data is that our analyses were based on cervical cancer and HIV estimates^8^, ^10^ which, although based on the best available data, might not be accurate for countries with insufficient surveillance information. Typically, these are the countries identified in this study as having high HIV-related cervical cancer burden.

A final limitation of our study is that we do not show estimates stratified by age, conscious of the fact that such an approach requires a systematic appraisal of appropriate age-specific RR estimates. Only one of the 24 studies included in the meta-analysis provided age-stratified RRs.^34^ This study reported an overall RR of 6, consistent with our findings, but reported decreasing RRs by age group. Although this age effect is the topic of further analyses and validation, it is beyond the scope of the present study.

In conclusion, our study presents the first global estimates of cervical cancer attributable to HIV. We show that women living with HIV have a six-fold higher risk of developing cervical cancer compared with women without HIV infection. In view of this increased risk, we estimate that nearly 5% of all cervical cancer cases worldwide are attributable to HIV infection. This attributable fraction varies greatly by region and is highest across southern and eastern Africa and some countries in western Africa, where coverage of HPV vaccination, and of cervical cancer screening and treatment for all women and girls, is suboptimal. It is important to improve access to cervical cancer prevention for all women in all settings, but particularly in countries where access to health services is difficult and HIV prevalence is high. Focusing on both cervical cancer prevention and HIV prevention, including integration of cervical cancer screening and treatment with HIV services, can optimise benefits in countries hardest hit by both epidemics. Such an approach in countries with the highest burden of HIV-attributable cervical cancer will contribute substantially to delivering on the WHO 2030 scale-up targets of 90% coverage of vaccination of girls, 70% coverage of screening, and 90% coverage of treatment leading to elimination of cervical cancer as a major public health problem in the future and reversal of the widening disparities in the disease burden seen today.

**This online publication has been corrected. The corrected version first appeared at thelancet.com/lancetgh on November 20, 2020**
