# Correction to *Lancet Glob Health* 2020; published online Nov 16. https://doi.org/10.1016/S2214-109X(20)30459-9

**DOI:** 10.1016/S2214-109X(20)30509-X

**Published:** 2020-11-20

*Stelzle D, Tanaka LF, Lee KK, et al. Estimates of the global burden of cervical cancer associated with HIV.* Lancet Glob Health *2020; published online Nov 16. https://doi.org/10.1016/S2214-109X(20)30459-9*—In this Article, (%) has been removed from the key in figure 4 parts B and C so it reads “ASIR per 100 000”. A new version of the appendix has been uploaded. These corrections have been made as of Nov 20, 2020.

