# Supplementary material for: Estimates of the global burden of cervical cancer associated with HIV
Source: Lancet Glob Health. 2020 Nov 16;9(2):e161–9. doi: 10.1016/S2214-109X(20)30459-9 (PMC7815633; doi:10.1016/S2214-109X(20)30459-9)
Supplement: Supplementary appendix [file mmc1.pdf]

THE LANCET
Global Health

## Supplementary appendix

This appendix formed part of the original submission and has been peer reviewed. We post it as supplied by the authors.

**This online publication has been corrected. The corrected version first appeared at thelancet.com/lancetgh on November 20, 2020.**

Supplement to: Stelzle D, Tanaka LF, Lee KK et al. Estimates of the global burden of cervical cancer associated with HIV. *Lancet Glob Health* 2020; published online Nov 16. http://dx.doi.org/10.1016/S2214-109X(20)30459-9.

SUPPLEMENTARY MATERIAL

# Estimates of the global burden of cervical cancer associated with HIV

Dominik Stelzle*[1,2], Luana F Tanaka*[2], Kuan Ken Lee[3], Ahmadaye Ibrahim Khalil[4], Iacopo Baussano[4], Anoop SV Shah[3,5], David A McAllister[6], Sami L Gottlieb[7], Stefanie J Klug[2], Andrea S Winkler[1,8], Freddie Bray[4], Rachel Baggaley[9], Gary M. Clifford[4], Nathalie Broutet[7] and Shona Dalal[9]

* Contributed equally

Affiliations:

1. Center for Global Health, Department of Neurology, Faculty of Medicine, Technical University of Munich, Germany
2. Chair of Epidemiology, Department of Sport and Health Sciences, Technical University of Munich, Germany
3. BHF Centre for Cardiovascular Science, University of Edinburgh, United Kingdom
4. International Agency for Research on Cancer, Lyon, France
5. Usher Institute of Population Health Sciences and Informatics, University of Edinburgh, United Kingdom
6. Institute of Health and Wellbeing, University of Glasgow, United Kingdom
7. Department of Sexual and Reproductive Health and Research, WHO, Geneva, Switzerland
8. Centre for Global Health, Institute of Health and Society, Faculty of Medicine, University of Oslo, Norway
9. Department of Global HIV, Hepatitis and STIs Programmes, WHO, Geneva, Switzerland

Correspondence and requests for reprints:
Dr Shona Dalal,
Department of Global HIV, Hepatitis and STIs Programmes
World Health Organization
Geneva
E-Mail: Dalals@who.int

## List of tables:

TABLE 1: SEARCH STRINGS FOR ALL FIVE DATABASES ..... 3
TABLE 2: CHARACTERISTICS OF INCLUDED STUDIES ..... 7
TABLE 3: CHARACTERISTICS OF INCLUDED STUDIES - CONTINUED ..... 13
TABLE 4: MOOSE CHECKLIST[61] ..... 19
TABLE 5. ESTIMATED PROPORTION OF CERVICAL CANCER ASSOCIATED WITH HIV AND PAF TO HIV IN 2018, BY UNAIDS REGIONS 27
TABLE 6: COUNTRY-LEVEL BURDEN OF CERVICAL CANCER ASSOCIATED WITH HIV* ..... 37
TABLE 7: DEFINITION OF WHO REGIONS ..... 49
TABLE 8. DEFINITION OF UN AFRICA SUBREGION ..... 54
TABLE 9: DEFINITION OF UNAIDS REGIONS ..... 56
TABLE 10: DEFINITION OF INCOME LEVEL BY THE WORLD BANK ..... 61
TABLE 11. COUNTRIES AND TERRITORIES WITH NO HIV ESTIMATES, FOR WHICH NO ESTIMATES OF HIV-ASSOCIATED CERVICAL CANCER BURDEN WERE CALCULATED IN THIS STUDY ..... 64

## List of figures:

FIGURE 1: FLOW CHART OF THE SEARCH AND INCLUDED STUDIES (29/04/2019) ..... 21
FIGURE 2: FUNNEL PLOT OF INCLUDED STUDIES FOR THE OVERALL ESTIMATE ..... 22
FIGURE 3. META-REGRESSION BY PROPORTION ON ANTIRETROVIRAL THERAPY ..... 26
FIGURE 4: PROPORTION OF WOMEN WITH CERVICAL CANCER LIVING WITH HIV, 2018 ..... 28
FIGURE 5: TOTAL NUMBER OF INCIDENT CERVICAL CANCER CASES AMONG WLHIV ..... 29
FIGURE 6: TOTAL NUMBER OF INCIDENT CERVICAL CANCER CASES ATTRIBUTABLE TO HIV ..... 30
FIGURE 7: PROPORTION OF WOMEN LIVING WITH HIV AMONG ALL NEW CERVICAL CANCER PATIENTS IN 2018, STRATIFIED BY WHO REGION ..... 31
FIGURE 8: POPULATION ATTRIBUTABLE FRACTION FOR HIV ASSOCIATED WITH CERVICAL CANCER, STRATIFIED BY WHO REGION ..... 32
FIGURE 9: TOTAL NUMBER OF NEW CERVICAL CANCER CASES ATTRIBUTABLE TO HIV IN 2018, STRATIFIED BY WHO REGION ..... 33
FIGURE 10: PROPORTION OF WOMEN LIVING WITH HIV AMONG ALL NEW CERVICAL CANCER PATIENTS IN 2018, STRATIFIED BY UNAIDS REGION ..... 34
FIGURE 11: POPULATION ATTRIBUTABLE FRACTION FOR HIV ASSOCIATED WITH CERVICAL CANCER, STRATIFIED BY UNAIDS REGION 35
FIGURE 12: TOTAL NUMBER OF NEW CERVICAL CANCER CASES ATTRIBUTABLE TO HIV IN 2018, STRATIFIED BY UNAIDS REGIONS ..... 36
FIGURE 13. ESTIMATED PROPORTION AND POPULATION ATTRIBUTABLE FRACTION FOR HIV ASSOCIATED CERVICAL CANCER, RANKED BY BURDEN PROPORTION FROM HIGHEST TO LOWEST, ACCORDING TO UNAIDS REGION ..... 44
FIGURE 14. ESTIMATED POPULATION ATTRIBUTABLE FRACTION FOR CERVICAL CANCER AND HIV, RANKED FROM HIGHEST TO LOWEST PROPORTION, RESTRICTED TO 50 HIGHEST RANKED COUNTRIES (BY UNAIDS REGION)┴ ..... 46
FIGURE 15. COUNTRIES WITH >250 CERVICAL CANCER CASES ATTRIBUTABLE TO HIV IN 2018, BY UNAIDS REGION┴ ..... 47
FIGURE 16. FOREST PLOTS FOR ANALYSES BY RISK MEASURE AND CONTINENT ..... 48

Table 1: Search strings for all five databases

| | |
|---|---|
| | HIV & Cervical Cancer<br>Translate: Pubmed, Embase, GIM, Web of Science, CABI<br>Filters: Epidemiological, Database source, Publication Type<br>Created by: Kavita Kothari/Tomas Allen (librarians) |

PUBMED DATABASE

| # | Searches |
|---|---|
| 1 | "HIV Infections"[Mesh] OR “HIV”[MeSH] OR “hiv”[tw] OR “hiv-1”[tw] OR “hiv-2”[tw] OR “hiv1”[tw] OR “hiv2”[tw] OR hiv infect*[tw] OR “human immunodeficiency virus”[tw] OR “human immunedeficiency virus”[tw] OR “human immuno-deficiency virus”[tw] OR “human immune-deficiency virus”[tw] OR ((human immun*) AND (“deficiency virus”[tw])) OR “acquired immunodeficiency syndrome”[tw] OR “acquired immunedeficiency syndrome”[tw] OR “acquired immuno-deficiency syndrome”[tw] OR “acquired immune-deficiency syndrome”[tw] OR ((acquired immun*) AND (“deficiency syndrome”[tw])) OR "Sexually Transmitted Diseases, Viral"[MeSH:NoExp] OR "HIV Long-Term Survivors"[Mesh] |
| 2 | “Uterine Cervical Neoplasms”[MeSH Terms] OR “cervical cancer”[Tiab] OR “Cancer of Cervix” [TiAb] OR “Cancer of the Cervix” [TiAB] OR Cervical Neoplasia* [TiAB] OR Cervix Neoplasm*[tiab] OR “Neoplastic Cervix” [TiAB] OR ((Cervix[tiab] OR Cervical[tiab]) AND (Cancer[tiab] OR Cancers[tiab] OR Cancerous[tiab] OR Carcinoma[tiab] OR Neoplasia*[tiab] OR Neoplasm*[tiab] OR Neoplastic[tiab])) |
| 3 | ((Cervix[tiab] OR Cervical[tiab]) AND (preCancer[tiab] OR preCancerous[tiab] OR dysplasia[tiab] OR abnormal growth[tiab] OR "atypical squamous"[tiab] OR "Intraepithelial lesions"[tiab])) OR "Atypical Squamous Cells of the Cervix"[Mesh] OR "Squamous Intraepithelial Lesions of the Cervix"[Mesh] OR "Uterine Cervical Dysplasia"[Mesh] |
| 4 | Papillomaviridae [MeSH Terms] OR papillomaviridae[Tiab] "human papilloma virus infection"[TIAB] OR "human papillomavirus infection"[TiAb] OR hpv[TiAb] OR "Human papillomaviruses"[Tiab] |
| 5 | prevalence[TW] OR incidence[TW] OR epidemiol*[TW] OR survey[TW] OR "rapid assessment"[TW] OR "situation assessment"[TW] OR "situational assessment"[TW] OR rar[TW] OR cohort[TW] OR surveillance[TW] OR seroprevalence[TW] OR seroincidence[TW] OR seroepidemiol*[TW] OR screening[TW] OR mortality[tiab] OR mortalities[tiab] OR mortal[tiab] OR dead[Tiab] OR death[tiab] OR deaths[tiab] OR decease*[tiab] O R demise*[tiab] OR die[tiab] OR died[tiab] OR dies[tiab] OR dying[tiab] OR fatal[tiab] OR fatality[tiab] OR fatalities[tiab] OR non-survival*[tiab] OR Nonsurvival*[tiab] OR survival*[tiab] OR lethality[tiab] OR "epidemiologic methods"[Mesh] OR "epidemiologic studies"[Mesh] OR "sentinel surveillance"[Mesh] OR "seroepidemiologic studies"[Mesh] OR "cohort studies"[Mesh] OR "longitudinal studies"[Mesh] OR "follow-up studies"[Mesh] OR "prospective studies"[Mesh ] OR "Diagnostic Services"[Mesh] OR "Mass Screening"[Mesh] OR "Mortality"[Mesh] OR "mortality"[Subheading] OR "Hospital Mortality"[Mesh] OR "Death"[Mesh] OR "Cause of Death"[Mesh] OR "Death Certificates"[Mesh] OR "Fatal Outcome"[Mesh] OR "Autopsy"[Mesh] OR "Survival Rate"[Mesh ] |
| 6 | #1 AND (#2 OR #3 OR #4) |
| 7 | #6 AND #5 |
| 8 | NOT ("animals"[MeSH] NOT "humans"[MeSH]) AND "humans"[MeSH] |

EMBASE DATABASE

| # | Searches |
|---|---|
| 1 | 'human immunodeficiency virus infection'/exp OR 'human immunodeficiency virus'/exp OR 'hiv survivor'/exp OR hiv:ti,ab,kw OR 'hiv-1':ti,ab,kw OR 'hiv-2':ti,ab,kw OR 'human immunodeficiency virus':ti,ab,kw OR 'human immuno deficiency':ti,ab,kw OR 'human immunedeficiency virus':ti,ab,kw OR 'human immune deficiency virus':ti,ab,kw OR 'human immune-deficiency virus':ti,ab,kw OR 'acquired immune-deficiency syndrome':ti,ab,kw OR 'acquired immunedeficiency syndrome':ti,ab,kw OR 'acquired immunodeficiency syndrome':ti,ab,kw OR 'acquired immuno-deficiency syndrome':ti,ab,kw |
| 2 | 'uterine cervix tumor'/exp OR 'cervical cancer':ti,ab,kw OR 'cancer of cervix':ti,ab,kw OR 'cancer of the cervix':ti,ab,kw OR 'neoplastic cervix':ti,ab,kw OR ((cervix:ti,ab,kw OR cervical:ti,ab,kw) AND (cancer:ti,ab,kw OR cancers:ti,ab,kw OR cancerous:ti,ab,kw OR carcinoma:ti,ab,kw OR neoplasia*:ti,ab,kw OR neoplasm*:ti,ab,kw OR neoplastic:ti,ab,kw)) |
| 3 | ((Cervix:ti,ab,kw OR Cervical:ti,ab,kw) AND (preCancer:ti,ab,kw OR preCancerous:ti,ab,kw OR dysplasia:ti,ab,kw OR 'abnormal growth':ti,ab,kw OR 'atypical squamous':ti,ab,kw OR 'Intraepithelial lesions':ti,ab,kw)) OR 'Atypical Squamous Cells of the Cervix'/exp OR 'squamous intraepithelial lesion of the cervix'/exp OR 'uterine cervix dysplasia'/exp |
| 4 | 'Papillomaviridae'/exp OR 'papillomaviridae':ti,ab,kw OR ‘human papilloma virus infection':ti,ab,kw OR 'human papillomavirus infection':ti,ab,kw OR hpv:ti,ab,kw OR 'Human papillomaviruses':ti,ab,kw |
| 5 | prevalence:ti,ab,de OR incidence:ti,ab,de OR epidemiol*:ti,ab,de OR survey:ti,ab,de OR 'rapid assessment':ti,ab,de OR 'situation assessment':ti,ab,de OR 'situational assessment':ti,ab,de OR rar:ti,ab,de OR cohort:ti,ab,de OR surveillance:ti,ab,de OR seroprevalence:ti,ab,de OR seroincidence:ti,ab,de OR seroepidemiol*:ti,ab,de OR screening:ti,ab,de OR 'mass screening':ti,ab,de OR mortality:ti,ab OR mortalities:ti,ab OR mortal:ti,ab OR dead:ti,ab OR death:ti,ab OR deaths:ti,ab OR decease*:ti,ab OR demise*:ti,ab OR die:ti,ab OR died:ti,ab OR dies:ti,ab OR dying:ti,ab OR fatal:ti,ab OR fatality:ti,ab OR fatalities:ti,ab OR non-survival*:ti,ab OR Nonsurvival*:ti,ab OR survival*:ti,ab OR lethality:ti,ab OR epidemiology:de OR 'Cancer epidemiology'/exp OR 'cancer statistics'/exp OR 'disease surveillance'/exp OR 'health survey'/exp OR 'incidence'/exp OR 'infection rate'/exp OR 'prevelance'/exp OR 'sentinel surveillance'/exp OR 'seroepidemiology'/exp OR 'cohort analysis'/exp OR 'longitudinal study'/exp OR 'prospective study'/exp OR 'preventive health service'/exp OR 'mass screening'/exp OR 'cancer mortality'/exp OR 'all cause mortality'/exp OR 'mortality':de OR 'hospital mortality'/exp OR 'mortality rate'/exp OR 'death'/exp OR 'cause of death'/exp OR 'dying'/exp OR 'fatality'/exp OR 'lethality'/exp OR 'death certificate'/exp OR 'autopsy'/exp OR 'cancer survival'/exp OR 'survival rate'/exp |
| 6 | #2 OR #3 OR #4 |
| 7 | #6 AND #1 |
| 8 | #7 AND #5 |
| 9 | #5 AND #7 AND [embase]/lim |
| 10 | #5 AND #7 AND [embase]/lim NOT ('letter'/it OR 'editorial'/it OR 'note'/it) |
|  | No human filter is used in embase since it is known not to be reliable. |

Global Health DATABASE

| # | Searches |
|---|---|
| **1** | DE "HIV infections" OR DE "HIV-1 infections" OR DE "HIV-2 infections" OR DE "HIV-1 infections" OR DE "HIV-2 infections" OR  "human immunodeficiency viruses" OR DE "Human immunodeficiency virus 1" OR DE "Human immunodeficiency virus 2" OR<br><br>TX (“hiv” OR “hiv-1” OR “hiv-2” OR “hiv1” OR “hiv2” OR  hiv infect* OR “human immunodeficiency virus” OR “human immunedeficiency virus” OR “human immuno-deficiency virus” OR “human immune-deficiency virus” OR ((human immun*) AND (“deficiency virus”)) OR “acquired immunodeficiency syndrome” OR “acquired immunedeficiency syndrome” OR “acquired immuno-deficiency syndrome” OR “acquired immune-deficiency syndrome” OR ((acquired immun*) AND (“deficiency syndrome”))) |
| **2** | DE "cervical cancer" OR TI (“cervical cancer” OR “Cancer of Cervix”  OR “Cancer of the Cervix”  OR Cervical Neoplasia*  OR Cervix Neoplasm* OR “Neoplastic Cervix”  OR ((Cervix OR Cervical) AND (Cancer OR Cancers OR Cancerous OR Carcinoma OR Neoplasia* OR Neoplasm* OR Neoplastic))) OR AB (“cervical cancer” OR “Cancer of Cervix”  OR “Cancer of the Cervix”  OR Cervical Neoplasia*  OR Cervix Neoplasm* OR “Neoplastic Cervix”  OR ((Cervix OR Cervical) AND (Cancer OR Cancers OR Cancerous OR Carcinoma OR Neoplasia* OR Neoplasm* OR Neoplastic))) |
| **3** | TI((Cervix OR Cervical) AND (preCancer OR preCancerous OR dysplasia OR abnormal growth OR "atypical squamous" OR "Intraepithelial lesions")) OR AB((Cervix OR Cervical) AND (preCancer OR preCancerous OR dysplasia OR abnormal growth OR "atypical squamous" OR "Intraepithelial lesions")) OR DE "cervical intraepithelial neoplasia" |
| **4** | DE "Papillomavirus" OR DE "Papillomavirus" OR TI (papillomaviridae "human papilloma virus infection" OR "human papillomavirus infection" OR hpv OR "Human papillomaviruses") OR AB (papillomaviridae "human papilloma virus infection" OR "human papillomavirus infection" OR hpv OR "Human papillomaviruses") |
| **5** | TX(prevalence OR incidence OR epidemiol* OR survey OR "rapid assessment" OR "situation assessment" OR "situational assessment"  OR rar OR cohort OR surveillance OR seroprevalence OR seroincidence OR seroepidemiol* OR screening) OR TI(mortality OR mortalities OR mortal OR dead OR death OR deaths OR decease* OR demise* OR die OR died OR dies OR dying OR fatal OR fatality OR fatalities OR non-survival* OR Nonsurvival* OR survival* OR lethality) OR AB(mortality OR mortalities OR mortal OR dead OR death OR deaths OR decease* OR demise* OR die OR died OR dies OR dying OR fatal OR fatality OR fatalities OR non-survival* OR Nonsurvival* OR survival* OR lethality) OR DE "epidemiology" OR DE "disease surveys" OR DE "incidence" OR DE "disease incidence" OR DE "sentinel surveillance" OR DE "serological surveys" OR DE "longitudinal studies" OR DE "mortality" OR DE "death and dying" OR DE "causes of death" OR DE "death" OR DE "postmortem examinations" OR DE "survival" |
| **6** | #2 OR #3 OR #4 |
| **7** | #6 AND #1 |
| **8** | #7 AND #5 |

Web of Science DATABASE

| # | Searches |
|---|---|
| 1 | TS=(“hiv” OR “hiv-1” OR “hiv-2” OR “hiv1” OR “hiv2” OR hiv infect* OR “human immunodeficiency virus” OR “human immunedeficiency virus” OR “human immuno-deficiency virus” OR “human immune-deficiency virus” OR ((human immun*) AND (“deficiency virus”)) OR “acquired immunodeficiency syndrome” OR “acquired immunedeficiency syndrome” OR “acquired immuno-deficiency syndrome” OR “acquired immune-deficiency syndrome” OR ((acquired immun*) AND (“deficiency syndrome”)) ) |
| 2 | TS=(“cervical cancer” OR “Cancer of Cervix” OR “Cancer of the Cervix” OR Cervical Neoplasia* OR Cervix Neoplasm* OR “Neoplastic Cervix” OR ((Cervix OR Cervical) AND (Cancer OR Cancers OR Cancerous OR Carcinoma OR Neoplasia* OR Neoplasm* OR Neoplastic))) |
| 3 | TS=(“cervical cancer” OR “Cancer of Cervix” OR “Cancer of the Cervix” OR Cervical Neoplasia* OR Cervix Neoplasm* OR “Neoplastic Cervix” OR ((Cervix OR Cervical) AND (Cancer OR Cancers OR Cancerous OR Carcinoma OR Neoplasia* OR Neoplasm* OR Neoplastic))) |
| 4 | TS=(Papillomaviridae OR papillomaviridae "human papilloma virus infection" OR "human papillomavirus infection" OR hpv OR "Human papillomaviruses") |
| 5 | TS=(prevalence OR incidence OR epidemiol* OR survey OR "rapid assessment" OR "situation assessment" OR "situational assessment" OR rar OR cohort OR surveillance OR seroprevalence OR seroincidence OR seroepidemiol* OR screening OR mortality OR mortalities OR mortal OR dead OR death OR deaths OR decease* OR demise* OR die OR died OR dies OR dying OR fatal OR fatality OR fatalities OR non-survival* OR Nonsurvival* OR survival* OR lethality OR "longitudinal studies" OR "follow-up studies" OR "prospective studies" OR "Mass Screening" OR "Autopsy") |
| 6 | #1 AND (#2 OR #3 OR #4) |
| 7 | w/ numerical filter |

Global Index Medicus (GIM)

| # | **Searches** |
|---|---|
| **1** | (Cervix OR Cervical) |
| **2** | (precancer* OR squamous OR Intraepithelial OR Cancer* OR Carcinoma OR Neoplasia OR Neoplasm* OR Neoplastic) |
| **3** | Papillomaviridae OR papilloma OR papillomavirus* OR hpv |
| **4** | AIDS OR HIV OR hiv1 OR hiv2 OR (immunodeficiency virus) OR (immune deficiency virus) OR (acquired immunodeficiency) OR (acquired immunedeficiency) OR (acquired immune deficiency) OR (acquired immune-deficiency) |
| **5** | Epidemiolog* OR Comparative<br>OR ASSESSMENT OR statistics OR numerical OR data OR<br>Evaluation OR meta OR analysis OR multicenter OR mortalit* OR incidence OR surveillance OR prevalence OR morbidit* OR systematic OR burden OR rate OR ratio OR survey OR rar OR cohort OR seroprevalence OR seroincidence OR seroepidemiol* OR screening OR dead OR death* OR decease* OR demise* OR dying OR fatal* OR Nonsurvival* OR survival* OR lethality OR survey OR sentinel OR seroepidemiology OR cohort OR longitudinal OR prospective |
| 6 | (#1 AND #2) OR #3 |
| **7** | #4 AND #5 AND #6 |
| 8 | LILACS (Americas) (80)<br>IMSEAR (South-East Asia) (18)<br>WPRIM (Western Pacific) (11)<br>AIM (Africa) (3)<br>IMEMR (Eastern Mediterranean) (1) |

Table 2: Characteristics of included studies

| Authors | Year | Country | Study type | Study period | Cohort/setting | Mean age | Ethnicity | ART coverage | CD4 count/mL (mean/ category) | Viral load copies/mL (mean/ category) | AIDS patients only |
|---|---|---|---|---|---|---|---|---|---|---|---|
| Abraham et al.[1] | 2013 | USA | cohort study | 1996–2010 | NA-ACCORD | 37 | white (22%), black (50%), other (28%) | 29% | 342, 30% (>500) | 9120 | No |
| Adjorlolo-Johnson et al.[2] | 2010 | Côte d'Ivoire | case-control study | 1997–1999 | — | 46.7 | — | | | — | No |
| Akarolo-Anthony et al.[3] | 2014 | Nigeria | cohort study | 2005–2012 | IHVN HIV cohort | 33 | — | — | — | — | No |
| Allerdice et al.[4] | 2003 | Scotland | registry linkage study (population-based) | 1981–1996 | Scotland's HIV database | 28 | — | — | — | — | No |
| | | | | | | | | | | | |
| Bedimo et al.[5] | 2009 | USA | cohort study | 1997–2004 | VACS Virtual Cohort | 45.8 | white (32%), black (43%), hispanic (8%), others (17%) | — | — | — | No |
| Carlander et al.[6] | 2016 | Sweden | cohort study | 1993–2011 | InfCare HIV, Swedish Population Register (SPR), Swedish National Cervical Screening Register (NKCx) | 31 | Swedish (28%), sub-Saharan Africa (50%), east (10%), other or unknown (10%) | 91% | 178, 23% (>500) | — | No |
| Castilho et al.[7] | 2015 | Brazil | cohort study | 1998–2010 | INI cohort | 36 | white (53%), non-white (47%) | 74% | 287 | 31600 | No |
| Chaturvedi et al.[8] | 2009 | USA | registry linkage study (population-based) | 1980–2004 | HACM | 37 | — | — | 100, 35.8% (>200) | — | Yes |
| Chaturvedi et al.[9] | 2008 | USA | registry linkage study (population-based) | 1980–2004 | HACM | — | — | — | — | — | Yes |
| Chen et al.[10] | 2015 | Taiwan | case-control study | 2000–2011 | Longitudinal Health Insurance Database (LHID) | — | — | 99% | — | — | No |
| Chen et al.[11] | 2014 | Taiwan | cohort study | 2000–2008 | Longitudinal Health Insurance Database (LHID) | 31.6 | — | 42% | — | — | No |
| Clifford et al.[12] | 2005 | Switzerland | cohort study | 1985–2003 | Swiss HIV Cohort Study and Swiss cantonal cancer registries | — | — | 26% | — | — | No |
| Cooksley et al.[13] | 1999 | USA | registry linkage study | 1981–1994 | Texas | 37 | — | — | — | — | No |

| Authors | Year | Country | Study type | Study period | Cohort/setting | Mean age | Ethnicity | ART coverage | CD4 count/mL (mean/ category) | Viral load copies/mL (mean/ category) | AIDS patients only |
|---|---|---|---|---|---|---|---|---|---|---|---|
| | | | (population-based) | | | | | | | | |
| Dal Maso et al.[14] | 2009 | Italy | registry linkage study (population-based) | 1986–2005 | Italian AIDS Registry and 24 Italian cancer registries | — | — | — | — | — | Yes |
| Engels et al.[15] | 2008 | USA | registry linkage study (population-based) | 1991–2002 | HACM | 35.8 | white (30%), black (53%), hispanic (17%) | — | 491 | — | No |
| Engels et al.[16] | 2006 | USA | registry linkage study (population-based) | 1980–2002 | HACM | 36.2 | white (52%), black (30%), hispanic (17%), other (1%) | — | — | — | No |
| Franceschi et al.[17] | 1998 | Italy | registry linkage study (population-based) | 1986–1992 | National Registry of AIDS and 13 Cancer Registries | — | — | — | — | — | Yes |
| Frisch et al.[18] | 2001 | USA | registry linkage study (population-based) | 1978–1996 | HACM | 35 | white (21%), black (57%), hispanic (21%), other (1%) | — | — | — | Yes |
| Galceran et al.[19] | 2007 | Spain | registry linkage study (population-based) | 1981–1999 | Tarragona and Girona Cancer Registries | — | — | | — | — | Yes |
| Gallagher et al.[20] | 2001 | USA | registry linkage study (population-based) | 1981–1994 | New York State Cancer Registry with the combined NYS and New York City AIDS registries | — | non-blacks (55%), blacks (45%) [among cases only] | 0% | — | — | Yes |
| Godbole et al.[21] | 2016 | India | registry linkage study (population-based) | 1991–2009 | — | 31 | — | — | — | — | No |
| Grabar et al.[22] | 2019 | France | registry linkage study (hospital-based) | 1992–2009 | FHDH-ANRS CO4 | 39 | sub-Saharan Africa (15%), outside sub-Saharan Africa (85%) | 0% | 288 | — | No |
| Hawes et al.[23] | 2006 | Senegal | cohort study | 1994–1998 | University of Dakar Infectious Disease Clinic (n = 4349); two sexually transmitted disease clinics in Dakar (n = 773) and in M’Bour (n = 270) | 31 | — | 0% | 402, 32% (>500) | 17400 | No |

| Authors | Year | Country | Study type | Study period | Cohort/setting | Mean age | Ethnicity | ART coverage | CD4 count/mL (mean/ category) | Viral load copies/mL (mean/ category) | AIDS patients only |
|---|---|---|---|---|---|---|---|---|---|---|---|
| Helleberg et al.[24] | 2014 | Denmark | cohort study | 1995–2011 | The Danish civil registration system, the Danish cancer registry, the Danish HIV cohort study, the Copenhagen general population study | 41 | Danish (70%), African (14%), Asian (6%), other (11%) | 77% | 450 | — | No |
| Hernández-Ramírez et al.[25] | 2017 | USA | registry linkage study (population-based) | 1996–2012 | HACM | 45 | Non-hispanic white (25%), Non-Hispanic black (47%), Hispanic or Latino (28%) | — | — | — | No |
| Hessol et al.[26] | 2004 | USA | registry linkage study (population-based) | 1994–1995 | WIHS cohort | 36 | African American (54%), Hispanic/Lati— (26.7%), White (16.9%), Other (2.4%) | 63% | 46.3% (<200), 29.9% (200–350), 30.8% (>350) | 52% (>20,000), 28% (<4000) | No |
| Hleyhel et al.[27] | 2013 | France | cohort study | 1992–2009 | FHDH-ANRS CO4 | 33 | Sub-Saharan women (8%) / non-Sub-Saharan (92%) | 4% | 259, 20% (>500) | — | No |
| Holmes et al.[28] | 2009 | Senegal | case-control study | 1998–2000 | OPD in Pikine (suburb of Dakar); Dantec Hospital at the University of Dakar | 48 | — | — | — | — | No |
| Jaquet et al.[29] | 2015 | Benin, Côte d'Ivoire, Nigeria and Togo | case-control study | 2009–2012 | Referral hospitals of Abidjan and Cotonou | 49 | Benin (5.5%), Cote d'Ivore (56.3%), Nigeria (34.0%), Togo (4.2%) | 64% | — | — | No |
| Kadhel et al.[30] | 2012 | Guadeloupe | registry linkage study (hospital-based) | 1999–2006 | Guadeloupian HIV Survey Health Centre | 37.2 | — | 63% | 31.4% (>500), 43.6% (200-499), 25% (<200) | — | No |

| Authors | Year | Country | Study type | Study period | Cohort/setting | Mean age | Ethnicity | ART coverage | CD4 count/mL (mean/ category) | Viral load copies/mL (mean/ category) | AIDS patients only |
|---|---|---|---|---|---|---|---|---|---|---|---|
| Kahesa et al.[31] | 2008 | Tanzania | case-control study | 2007–2007 | Ocean Road Cancer Institute, Dar-es-salaam | 50 | — | — | — | — | No |
| Keller et al.[32] | 2015 | USA | case-control study | 1994–2002 | WIHS cohort | 34 | Black (52%), Hispanic (29%), White (16%), other (3%) | 16% | 25% (<200), 18% (200–349), 24% (350–499), 43% (>500) | 44% (<4,000 copies/mL), 23% (4,000–20,000 copies/mL), 33% (>20,000 copies/mL/mL) | No |
| Khuakoonratt et al.[33] | 2008 | India | cohort study | 2001–2005 | Bangkok Metropolitan Administration Medical College and Vajira Hospital | 39 | — | — | — | — | No |
| Mahale et al.[34] | 2018 | USA | registry linkage study (population-based) | 1996–2012 | HACM: 50+ | 56 | Non-Hispanic white (25.8%), Non-Hispanic black (47.6%), Hispanic (26.6%) | — | — | — | No |
| Massad et al.[35] | 2008 | USA | cohort study | 1994–2005 | WIHS cohort | 19.6 | Black (47%), Hispanic (43%), White Non-Hispanic and other (9%) | 18% | 40% (>500), 51% (200–500), 9% (<200) | 40% (≤4000), 60% (>4000) | No |
| Massad et al.[36] | 2017 | USA | registry linkage study | 1994–2015 | WIHS cohort | 35 | Black (49%), Hispanic (33%), Whitne Non-Hispanic/Other (18%) | — | — | — | No |
| Mayans et al.[37] | 1999 | Spain | registry linkage study (population-based) | 1994–1996 | Catalan population-based cancer registry; Catalan AIDS registry | 31.6 | — | 87% | 226.3 | — | Yes |
| Mayor et al.[38] | 2016 | Puerto Rico | registry linkage study (population-based) | 1991–2009 | Retrovirus Research Center (RCC) HIV cohort, Puerto Rico Central Cancer Registry (PRCCR) data and the Puerto Rican Morality Registry (PRMR) | 36.6 | Hispanic (100%) | 0% | 49% (<200) | — | No |
| Mbulaiteye et al.[39] | 2006 | Uganda | registry linkage study (population-based) | 1988–2002 | The AIDS Support Organization (TASO), Kampala Cancer Registry (KCR); Kyadondo County | 30 | — | — | — | — | No |
| Moscicki et al.[40] | 2004 | USA | cohort study | 1996–2000 | REACH; 13-18 yo girls without HSIL | 16.7 | African American (75.8%), White and other (24.2%) | — | 4.7% (<200), 39.9% (200–500), 55.4% (>500) | 73.1% (<10,000) | No |

| Authors | Year | Country | Study type | Study period | Cohort/setting | Mean age | Ethnicity | ART coverage | CD4 count/mL (mean/ category) | Viral load copies/mL (mean/ category) | AIDS patients only |
|---|---|---|---|---|---|---|---|---|---|---|---|
| Mpunga et al.[41] | 2018 | Rwanda | case-control study | 2012–2016 | Butaro Cancer Centre of Excellence | 41 | — | — | — | — | No |
| Newnham et al.[42] | 2005 | England | registry linkage study (population-based) | 1985–2001 | Southeast England | — | — | — | — | — | No |
| Newton et al.[43] | 2001 | Uganda | case-control study | 1994–1998 | Kampala: Mulago (including the Uganda Cancer Institute), Nsambya, Mengo and Rubaga | 45 | — | — | — | — | No |
| Phelps et al.[44] | 2001 | USA | cohort study | 1993–1995 | HERS and SEER cohort | 35 | Black (58%), White (24%), Hispanic (16%), native American/Asian and other (2%) | — | 443 (only cervical cancer patients) | — | No |
| Salters et al.[45] | 2016 | Canada | registry linkage study (population-based) | 1994–2008 | British Columbia Cancer Agency, British Columbia Centre for Excellence in HIV/AIDS | 40 | — | 100% | 150 | — | No |
| Sekirime et al.[46] | 2007 | Uganda | case-control study | 1993–1995 | Mulago Hospital, Kampala, Uganda | 45 | — | — | — | — | No |
| Selik et al.[47] | 1998 | USA | registry linkage study | 1990–1995 | cancer registry (U.S. Bureau of the Census) and HIV registry | — | — | — | — | — | No |
| Serraino et al.[48] | 1999 | Italy/France | registry linkage study (population-based) | 1988–1998 | DMI-2, ISS, San Patrignano Community clinical database | 28 | — | — | — | — | No |
| Silverberg et al.[49] | 2009 | USA | cohort study | 1996–2007 | Kaisers Permanente Northern and Southern Californian HIV Registry, SEER, California Cancer Registry (CCR) | 41 | Asian/Pacific (7.2%), Black/African-American (7.5%), Hispanic/Latino (15.2%), White (28.8%), other (0.7%), unknown (40.6%) | 27% | 388.9 | — | No |
| Silverberg et al.[50] | 2018 | USA | case-control study | 1996–2014 | Kaisers Permanente Northern California HIV Registry, Northern California Cancer Registry (CCR) | 35.6 | Non-Hispanic White (47%), Non-Hispanic Black (8%), Hispanic (20%), other (19%), unknown (5%) | — | — | — | No |

| Authors | Year | Country | Study type | Study period | Cohort/setting | Mean age | Ethnicity | ART coverage | CD4 count/mL (mean/ category) | Viral load copies/mL (mean/ category) | AIDS patients only |
|---|---|---|---|---|---|---|---|---|---|---|---|
| Sitas et al.[51] | 1997 | South Africa | case-control study | 1992–1995 | Johannesburg Hospital, Hillbrow Hospital (Johannesburg) and Baragwanath Hospital in Soweto | — | Black (100%) | — | — | — | No |
| Stein et al.[52] | 2008 | South Africa | case-control study | 1995–2004 | Johannesburg Hospital, Hillbrow Hospital (Johannesburg) and Baragwanath Hospital in Soweto | 48 | — | — | — | — | No |
| Tanaka et al.[53] | 2018 | Brazil | registry linkage study (population-based) | 1997–2012 | Population-based Cancer Registry of Sao Paulo (PBCR-SP), AIDS notification database (SINAN) | — | white (27.8%), black (6.7%), asian (0.4%), pardo (15.2%), Indigineous (0.1%), unknown (49.9%) | — | — | — | Yes |
| Tanon et al.[54] | 2012 | Côte d'Ivoire, Benin | cohort study | 2009–2011 | IeDEA West Africa collaboration, referral hospitals of Abidjan, Côte d'Ivoire and Cotonou, Benin | 48 | — | — | — | — | No |
| Thorsteinsson et al.[55] | 2016 | Denmark | registry linkage study (population-based) | 1995–2010 | Danish HIV Cohort Study (DHCS), the Danish Pathology Data Bank (DPDB), the Danish Cancer Registry (DCR), the Civil Registration System (CRS) | 33.6 | White (48.7%), Asian (10.8%), Black (37.7%), other (2.8%), missing (18%) | — | 27.9% (<200cells), 27.2% (200–350cells), 44.9% (>350cells) | — | Yes |
| Uribe Parra et al.[56] | 2017 | Colombia | registry linkage study (population-based) | 1998–2015 | CAC population-based cancer registry, Colombian National HIV Registry | — | — | 95% | 100% (<200cells) | — | No |
| Vogel et al.[57] | 2011 | Germany | registry linkage study (pop.-based) | 1996–2009 | Bonn single centre HIV cohort; Saarland reference population | 36 | — | 40% | 359, 35% (<200) | 7940 | No |
| Whitham et al.[58] | 2017 | Senegal | cohort study | 1994–2010 | two infectious disease, two family planning, and two sexually transmitted disease clinics in or around Dakar. | 35 | — | 31% | 406, 25.1% (<200) | — | No |
| Zhang et al.[59] | 2011 | China | registry linkage study (hospital-based) | 2004–2008 | Centre of AIDS prevention and cure of Zhongnan Hospital, Wuhan Cancer Registry | — | — | — | — | — | No |
| Zohar et al.[60] | 2015 | Israel | registry linkage study (hospital-based) | 1981–2010 | National HIV/AIDS Registry (NHAR), Israeli Cancer Registry | 35 | Jewish (78%), non-Jewish (22%) | 0% | — | — | Yes |

Table 3: Characteristics of included studies - continued

| Authors | Risk measure | Number WLHIV | Number HIV negative women | Comparison group | Follow-up length (years) | Follow-up person years | Outcome | Case definition | Number cases outcome | Included in overall estimate? | Risk of bias |
|---|---|---|---|---|---|---|---|---|---|---|---|
| Abraham et al.[1] | SIR | 13690 | 12021 | general population | 4.5 | 61605 | ICC | ICD-O-3, ICD-9 | 17 | Yes | Low/ moderate |
| Adjorlolo-Johnson et al.[2] | OR | 32 | 220 | HIV negative women | – | – | ICC | 1986 International Federation of Gynecology and Obstetrics (FIGO) architectural staging system; histologically confirmed cancer | 132 | Yes | High |
| Akarolo-Anthony et al.[3] | SIR | 10580 | – | general population | – | – | ICC | ICD-O-3 | 8 | Yes | High |
| Allardice et al.[4] | SIR | 619 | – | general population | – | 4236 | ICC | ICD-9 | 1 | No, because of too few cases | High |
| Bedimo et al.[5] | IRR | – | 1429 | HIV negative women | 5.1 | – | ICC | ICD-9-CM | 12 | Yes | Low/ moderate |
| Carlander et al.[6] | IR | 893 | 205842 | HIV negative women | 7.2 | 2.8 million | CIN2+ | Systematized Nomenclature of Medicine (SNOMED) | 117 | No, because of outcome measure | Low/ moderate |
| Castilho et al.[7] | SIR | 978 | – | general population | 3.5 | – | ICC | – | 0 | No, because too few cases | High |
| Chaturvedi et al.[8] | SIR | 96303 | – | general population | – | – | ICC | ICD-O-3 C530–539 | 192 | No, because of shorter study period than Hernandez et al. (2017) | Low/ moderate |
| Chaturvedi et al.[9] | SIR | 96303 | – | general population | – | – | ICC | ICD-O-3 C530–539 | 101 | No, because of shorter study period than Hernandez et al. (2017) | Low/ moderate |
| Chen et al.[10] | SIR | 1115 | 4460 | HIV negative women | 4.6 | 61883 | ICC | CD-9-CM 180 | 4 | No, because the cohort is smaller than the cohort in Chen et al. (2014) | Low/ moderate |
| Chen et al.[11] | SIR | 1360 | 358141 | HIV negative women | 3.2 | 4377 | ICC | CD-9-CM 180 | 7 | Yes | High |
| Clifford et al.[12] | SIR | 2045 | – | general population | 3.9 | 8074 | ICC | ICD-O-2 and ICD-10 | 6 | Yes | High |
| Cooksley et al.[13] | SIR | – | – | general population | – | – | ICC | ICD-O | 13 | Yes | High |

| Authors | Risk measure | Number WLHIV | Number HIV negative women | Comparison group | Follow-up length (years) | Follow-up person years | Outcome | Case definition | Number cases outcome | Included in overall estimate? | Risk of bias |
|---|---|---|---|---|---|---|---|---|---|---|---|
| Dal Maso et al.[14] | SIR | 4891 | – | general population | 2.3 | 11062 | ICC | ICD-10 C53 | 30 | No, because only AIDS patients were included | Low/ moderate |
| Engels et al.[15] | SIR | 19785 | – | general population | 3.2 | 63312 | ICC | ICD-O-3 | 28 | No, because of shorter study period than Hernandez et al. (2017) | Low/ moderate |
| Engels et al.[16] | SIR | 60485 | – | general population | | | ICC | ICD-O-3 | 10 | No, because of shorter study period than Hernandez et al. (2017) | Low/ moderate |
| Franceschi et al.[17] | SIR | 1266 | – | general population | – | – | ICC | ICD-9 180 | 1 | No, because of shorter study period than Dal Maso et al. (2018) | High |
| Frisch et al.[18] | RR | 48949 | – | general population | – | – | ICC | ICD-O-2 | 46 | No, because only AIDS patients were included | Low/ moderate |
| Galceran et al.[19] | SIR | 355 | – | general population | 6.1 | 1509 | ICC | ICD-10, ICD-O-2 | 10 | No, because only AIDS patients were included | High |
| Gallagher et al.[20] | SIR | – | – | general population | – | – | ICC | ICD-9 | 133 | No, because only AIDS patients were included | High |
| Godbole et al.[21] | SIR | 11710 | – | general population | 4 | 46840 | ICC | ICD-9, ICD-10 | 23 | Yes | Low/ moderate |
| Grabar et al.[22] | SIR | 28977 | – | general population | – | 30223 | ICC | ICD9:180.x and ICD10:C53.x | 14 | Yes (four estimates by the four study periods: 1992–1996, 1997–2000, 2001–2004, 2005–2009) | High |
| Hawes et al.[23] | HR | 165 | 381 | commercial sex workers and women older than 15 years | 2.2 | 1380 | HSIL | – | 70 | No, because of shorter study period than Whithman et al. (2017) | High |

| Authors | Risk measure | Number WLHIV | Number HIV negative women | Comparison group | Follow-up length (years) | Follow-up person years | Outcome | Case definition | Number cases outcome | Included in overall estimate? | Risk of bias |
|---|---|---|---|---|---|---|---|---|---|---|---|
| Helleberg et al.[24] | IRR | 820 | 2600 | general population | 5.2 | 4592 | ICC | ICD-10 C53 | 2 | No, because of too few cases | High |
| Hernández-Ramírez et al.[25] | SIR | 129995 | – | general population | – | 893325 | ICC | ICD-O | 428 | Yes | Low/ moderate |
| Hessol et al.[26] | SIR | 1554 | 396 | general population | 5.1 | 7909 | ICC | ICD-O-2 | 1 | No, because of shorter study period than Massad et al. (2017) | High |
| Hleyhel et al.[27] | SIR | 11314 | – | general population | 2.6 | 29198 | ICC | ICD-9, ICD-10 | 39 | No, because no quality check on diagnosis was made as opposed to the study by Grabar et al. (2019) which used the same cohort. | Low/ moderate |
| Holmes et al.[28] | OR | 16 | 645 | Patients from an outpatient department or primary care centre in Dakar | – | – | ICC | cytology; diagnosis identified by biopsy | 150 | Yes (three estimates by HIV type: HIV-1, HIV-2, HIV-1 and HIV-2) | High |
| Jaquet et al.[29] | OR | 127 | 864 | all adult patients seeking care with a confirmed diagnosis of cancer | – | – | ICC | ICD-O-3 | 258 | Yes | High |
| Kadhel et al.[30] | SIR | 1232 | – | general population | 6.3 | 7738 | ICC | histologically confirmed cervical lesions (ICC or CIN) | 3 | No, because too few cases | High |
| Kahesa et al.[31] | OR | 45 | 231 | center attendants and visitors | – | – | ICC | histologically confirmed cancer of the cervix | 138 | Yes | High |

| Authors | Risk measure | Number WLHIV | Number HIV negative women | Comparison group | Follow-up length (years) | Follow-up person years | Outcome | Case definition | Number cases outcome | Included in overall estimate? | Risk of bias |
|---|---|---|---|---|---|---|---|---|---|---|---|
| Keller et al.[32] | HR | 1021 | 518 | general population | 5 | 7650 | CIN2+ | 2001 Bethesda System criteria for cytologic diagnosis, colposycopy | – | No, because of shorter study period than Massad et al. (2017) | Low/ moderate |
| Khuakoonratt et al.[33] | RR | 5 | 221 | women undergoing PAP smear who have LSIL | – | – | ICC | Bethesda criteria | 37 | No, because of poor data quality; study design seems to be cross-sectional | High |
| Mahale et al.[34] | SIR | 183542 | – | – | – | 928194 | ICC | ICD-O-3 | 87 | No, because only a subgroup of the HACM cohort (women aged 50 years and older) was analysed | Low/ moderate |
| Massad et al.[35] | RR | 45 | 87 | HIV negative women with acknowledged HIV risk behavior | 2.6 | – | CIN2+; HSIL | Bethesda System for cervicovaginal cytological diagnosis | 8 | No, because of shorter study period than Massad et al. (2017) | Low/ moderate |
| Massad et al.[36] | SIR | 1807 | 488 | HIV negative women with acknowledged HIV risk behavior | 11.8 | 27176 | ICC | Bethesda System for cervicovaginal cytological diagnosis | 4 | Yes | Low/ moderate |
| Mayans et al.[37] | IRR | 56 | – | – | – | – | ICC | – | 56 | No, because of shorter study period than Galceran et al. (2007) | Low/ moderate |
| Mayor et al.[38] | SIR | 502 | – | – | – | – | ICC | ICD-9, ICD-10, ICD-O-3 | 8 | Yes (estimates for the three study periods: 1992–1995, 1996–2002, 2003–2009) | High |
| Mbulaiteye et al.[39] | SIR | 8423 | – | – | 5 | – | ICC | ICD-O-2 | 27 | Yes | High |
| Moscicki et al.[40] | HR | 84 | 172 | HIV negative women without HSIL | 2.8 | 707 | HSIL | Bethesda criteria | 41 | No, because of outcome measure | High |
| Mpunga et al.[41] | OR | – | – | non-cancer patients | – | – | ICC | ICD-O-3 and ICD-10 | 560 | Yes | High |
| Newnham et al.[42] | SIR | 7110 | – | general population | – | 31098 | ICC | ICD-10 | 3 | No, because of too few cases | High |

| Authors | Risk measure | Number WLHIV | Number HIV negative women | Comparison group | Follow-up length (years) | Follow-up person years | Outcome | Case definition | Number cases outcome | Included in overall estimate? | Risk of bias |
|---|---|---|---|---|---|---|---|---|---|---|---|
| Newton et al.[43] | OR | 45 | 132 | non-malignant conditions and cancers not known to be caused by infections | – | – | ICC | Laboratory diagnosis of cancer | 65 | No | High |
| Phelps et al.[44] | IRR | 871 | 439 | HIV negative women with acknowledged HIV risk behavior | 5 | 6488 | ICC | National Death Index Plus (NDI Plus), ICD-9, ICD-10 | 5 | Yes | High |
| Salters et al.[45] | SIR | 2211 | – | general population | 5.7 | 12529 | ICC | ICD-9, ICD-10 | 22 | Yes | High |
| Sekirime et al.[46] | OR | 33 | 199 | women attending the gynecological or medical outpatient clinics of the same hospital for illnesses other than ICC | – | – | ICC | histologically confirmed diagnosis | 116 | Yes | High |
| Selik et al.[47] | RR | NA | – | 25–44 year old HIV negative women | 6 | 720000 | ICC | ICD-9 | 12 | No, because the study only examines cervical cancer mortality | High |
| Serraino et al.[48] | SIR | 2141 | – | general population France and Italy (15–49 years) | 4.2 | 9070 | ICC | ICD-9 | 10 | Yes | High |
| Silverberg et al.[50] | RR | 1935 | 19322 | active Kaiser Permanente health plan members (>18 years) | 4.9 | 105000 | ICC | All registries follow SEER practices in verifying and coding incident cancers. SEER requirements include categorization of histopathology, invasiveness, tumor size, extension, and lymph node involvement. | 8 | No, because of shorter study period than Silverberg et al. (2009) | Low/ moderate |
| Silverberg et al.[49] | OR | 115 | 120175 | Non-CIN2+ patients | – | – | CIN2+ | Systematized Nomenclature of Medicine | 20146 | No, because of outcome measure | Low/ moderate |
| Sitas et al.[51] | OR | – | – | cancer patients with cancer believed not to be of infectious origin | – | – | ICC | ICD-O-2 | 180 | No, because of the selection of controls (patients with cancer believed not to be of infectious origin) | High |
| Stein et al.[52] | OR | – | – | subjects with other cancer types | – | – | ICC | ICD-O-2 C53 | 1586 | No, because of the selection of controls (patients with cancer | High |

| Authors | Risk measure | Number WLHIV | Number HIV negative women | Comparison group | Follow-up length (years) | Follow-up person years | Outcome | Case definition | Number cases outcome | Included in overall estimate? | Risk of bias |
|---|---|---|---|---|---|---|---|---|---|---|---|
| | | | | | | | | | | believed not to be of infectious origin) | |
| Tanaka et al.[53] | SIR | – | – | general population | – | 142161 | ICC | – | 114 | No, because only AIDS patients | Low/ moderate |
| Tanon et al.[54] | OR | 93 | 488 | HIV negative women part of the IeDEA West Africa collaboration | – | – | ICC | ICD-O-3 C53 | 153 | Yes | High |
| Thorsteinsson et al.[55] | SIR | 1140 | 17046 | HIV negative women of Denmark, matched for age (15 for each WLHIV) | 9.1 | 166356 | CIN2+ | Systemized Nomenclature of Medicine (SNOMED) codes T8x3* and T83*, 2001 Bethesda criteria, ICD-10 codes C53 | 407 | No, because of outcome measure | Low/ moderate |
| Uribe Parra et al.[56] | SIR | 34076 | – | general population | | | ICC | ICD-10 | 11 | No, because only AIDS patients were included | High |
| Vogel et al.[57] | SIR | 280 | – | general population | 5.9 | 8772 | ICC | ICD-9 (180, 233.1), ICD-O-3 | 6 | Yes | High |
| Whitham et al.[58] | SIR | 618 | 702 | HIV negative women | 2 | 2640 | HSIL | Bethesda criteria | 28 | No, because of outcome measure | High |
| Zhang et al.[59] | SIR | 4982 | – | general population | 3.1 | 4892 | ICC | ICD-10 | 14 | Yes | High |
| Zohar et al.[60] | SIR | 3485 | – | general population | 7 | 24530 | ICC | ICD-O-3 | 16 | No, because no confidence intervals were reported and because the data are questionable | Low/ moderate |

Table 4: MOOSE checklist[61]

| Item No | Recommendation | Reported on Page No |
|---|---|---|
| **Reporting of background should include** | | |
| 1 | Problem definition | p.1 |
| 2 | Hypothesis statement | p.2 |
| 3 | Description of study outcome(s) | p.2 |
| 4 | Type of exposure or intervention used | p.2 |
| 5 | Type of study designs used | p.2 |
| 6 | Study population | p.2 |
| **Reporting of search strategy should include** | | |
| 7 | Qualifications of searchers (eg, librarians and investigators) | Appendix Table 1, p.3 |
| 8 | Search strategy, including time period included in the synthesis and key words | Appendix Table 1, p.3–6 |
| 9 | Effort to include all available studies, including contact with authors | No additional contact pursued |
| 10 | Databases and registries searched | Appendix Table 1, p.3–6 |
| 11 | Search software used, name and version, including special features used (eg, explosion) | Not mentioned |
| 12 | Use of hand searching (eg, reference lists of obtained articles) | Appendix Figure 1, p.21 |
| 13 | List of citations located and those excluded, including justification | Appendix Figure 1, p.21 |
| 14 | Method of addressing articles published in languages other than English | No additional methods |
| 15 | Method of handling abstracts and unpublished studies | Appendix Figure 1, p.21 |
| 16 | Description of any contact with authors | Not applicable |
| **Reporting of methods should include** | | |
| 17 | Description of relevance or appropriateness of studies assembled for assessing the hypothesis to be tested | Appendix Tables 2/3, p.7–18 |
| 18 | Rationale for the selection and coding of data (eg, sound clinical principles or convenience) | p.2 |
| 19 | Documentation of how data were classified and coded (eg, multiple raters, blinding and interrater reliability) | p.2 |
| 20 | Assessment of confounding (eg, comparability of cases and controls in studies where appropriate) | p.4, Figure 2; Appendix Table 3, p.13–18 |
| 21 | Assessment of study quality, including blinding of quality assessors, stratification or regression on possible predictors of study results | p.3 |
| 22 | Assessment of heterogeneity | p.3, Figure 1 |
| 23 | Description of statistical methods (eg, complete description of fixed or random effects models, justification of whether the chosen models account for predictors of study results, dose-response models, or cumulative meta-analysis) in sufficient detail to be replicated | p.3, Appendix p.23–25 |
| 24 | Provision of appropriate tables and graphics | p.3, Table 1, p.3–6; Figures 1–4; Appendix Tables/Figures |
| **Reporting of results should include** | | |
| 25 | Graphic summarizing individual study estimates and overall estimate | p.3, Figure 1 |
| 26 | Table giving descriptive information for each study included | Appendix Table 2, p.7–12 |
| 27 | Results of sensitivity testing (eg, subgroup analysis) | p.4, Figure 2; Appendix Table 11, p.48 |
| 28 | Indication of statistical uncertainty of findings | p.4, Table 1; p.3–4, Figures 1 and 2 |
| **Reporting of discussion should include** | | |
| 29 | Quantitative assessment of bias (eg, publication bias) | p.4, Figure 2; Appendix Figure 2, p.22 |

| | | |
|---|---|---|
| 30 | Justification for exclusion (eg, exclusion of non-English language citations) | Appendix Figure 1, p.21 |
| 31 | Assessment of quality of included studies | Appendix Table 3, p.13–18 |
| **Reporting of conclusions should include** | | |
| 32 | Consideration of alternative explanations for observed results | p.7–8 |
| 33 | Generalization of the conclusions (ie, appropriate for the data presented and within the domain of the literature review) | p.8 |
| 34 | Guidelines for future research | p.8 |
| 35 | Disclosure of funding source | p.3 |

Figure 1: Flow chart of the search and included studies (29/04/2019)

**PRISMA 2009 Flow Diagram**[62]

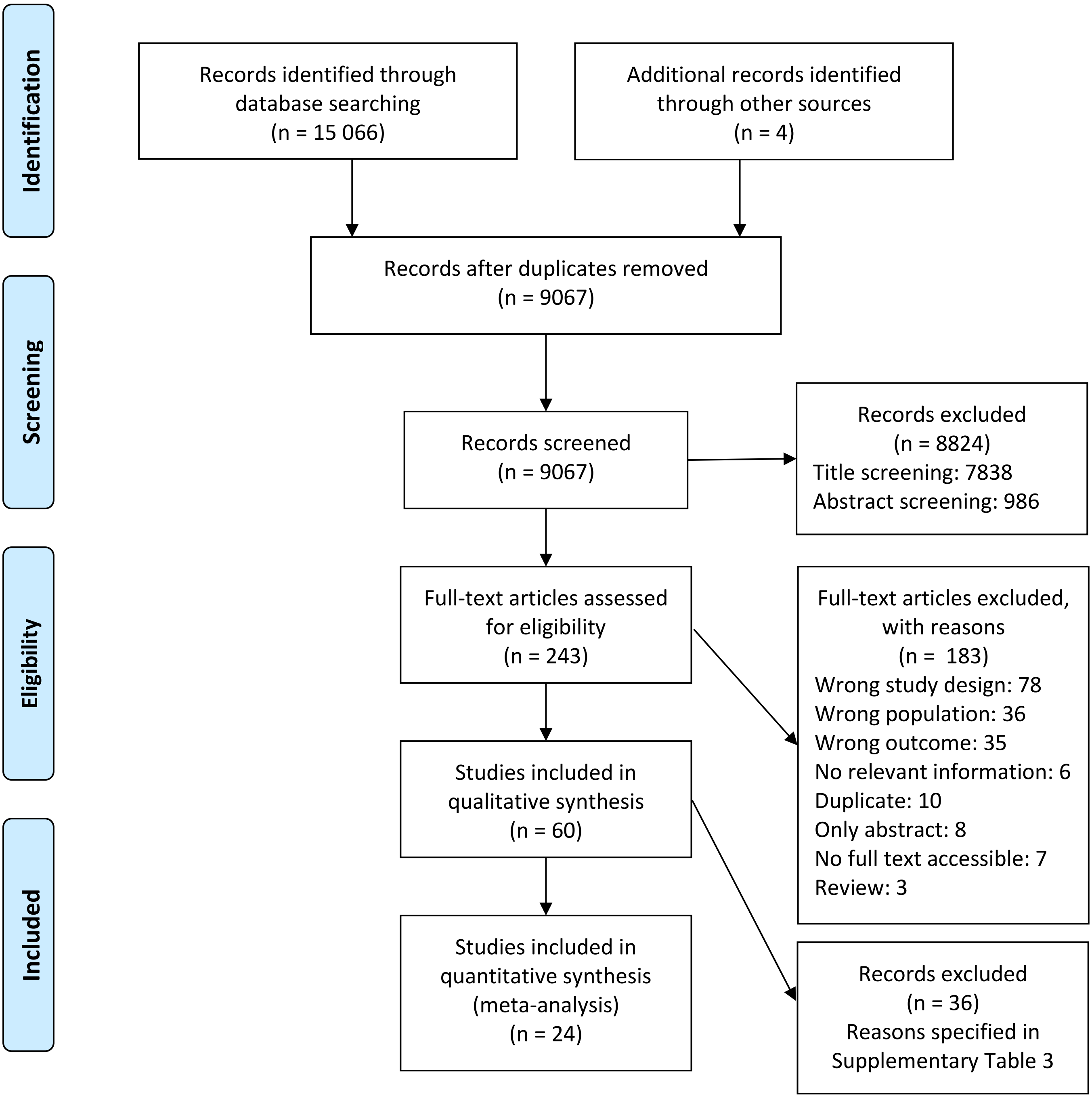

Figure 2: Funnel plot of included studies for the overall estimate

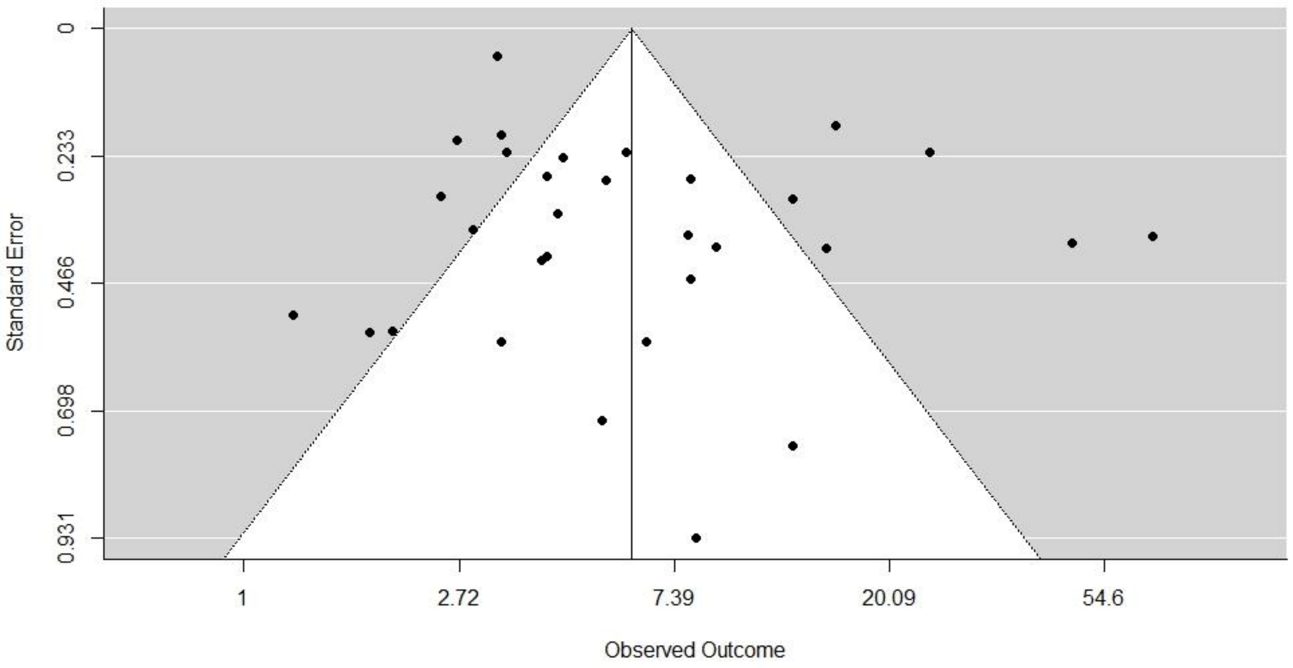

Method for calculating country-specific estimates for cervical cancer attributable to HIV

*Step 1*
We performed a systematic literature review and meta-analysis to calculate the pooled risk ratio for cervical cancer associated with HIV infection. We anticipated heterogeneity between studies when estimating the risk ratio due to different study designs, methods of analysis and varying adjustment, and geographical and population differences. We therefore used a random effects model to account for both within and between study heterogeneity.

*Step 2*
At a study level, we explored interaction between ART coverage and risk for cervical cancer among adult females (Supplementary figures 3/4).

*Step 3*
We used a random-effects model to account for within and between study heterogeneity. We extracted risk ratios for cervical cancer among WLHIV compared to HIV negative women from the relevant studies. The risk ratios were then pooled using a random effects model with the rma function in the metafor package in R.

*Step 4*
We obtained age-specific HIV prevalence rates (female adults aged 15 years and older) for 2018 for 170 countries from UNAIDS. Similarly, we obtained age-specific (female adults 15+ years) estimates of cervical cancer cases in 2018 from GLOBOCAN for 185 countries. We had both data types for 170 countries.

*Step 5*
Assumptions:

- We assumed that the pooled risk ratios obtained from a range of registry linkage, cohort and case-control studies were applicable across both low- and middle-income, and high-income countries.
- The degree of adjustment for confounders varied between the primary studies and therefore estimates may be limited by residual confounding.
- No correlation within studies was taken into account (for studies with more than one estimate); the estimates were considered to be independent

*Step 6*
We estimated the population attributable fraction – including the total numbers and the proportion of WLHIV among new cervical cancer patients – as follows:

$$Population\ Attributable\ Fraction\ (PAF)_c = \frac{HIV\ prevalence_c * \ (RR-1)}{1 + HIV\ prevalence_c * (RR-1)}$$

$$Proportion\ of\ women\ with\ cervical\ cancer\ living\ with\ HIV\ (Proportion)_c$$

$$= \frac{HIV\ prevalence_c * \ RR}{(1 - HIV\ prevalence_c) + (HIV\ prevalence_c * \ RR)}$$

*c country-specific*

Multiplying the *PAF* and the *Proportion* with the total number of cervical cancer cases yielded the total number of cervical cancer cases attributable to HIV and the total number of new cervical cancer patients living with HIV, respectively.

$$N_{\text{cervical cancer cases attributable to HIV}} = PAF_c * N_{\text{cervical cancer cases}}$$

$$N_{\text{cervical cancer patients living with HIV}} = Proportion_c * N_{\text{cervical cancer cases}}$$

*c country-specific*

*Step 7*

We estimated the uncertainty intervals for cervical cancer cases associated with HIV infection using simulation. We sampled from the probability distributions which captured the uncertainty around the estimate for each component (specifically, log normal for rate ratios, normal for the cervical cancer numbers and beta for the prevalence of HIV infection) calculating the PAF, proportion and numbers for each of 10,000 samples. Total numbers for each country were reported with uncertainty estimates as 95% confidence interval. The uncertainty ranges can be viewed as approximate Bayesian posterior probability limits with very diffuse priors for the estimates and risk factor rate ratios.

Example for Zimbabwe:

The HIV prevalence is represented as 10,000 samples of a variable following a beta distribution (left upper figure). The pooled risk ratio relative risk estimator of interest is presented in the bottom left figure as a histogram of 10 000 samples of a normally distributed variable. Using the above-mentioned formula, we simulated 10 000 samples of the PAF.

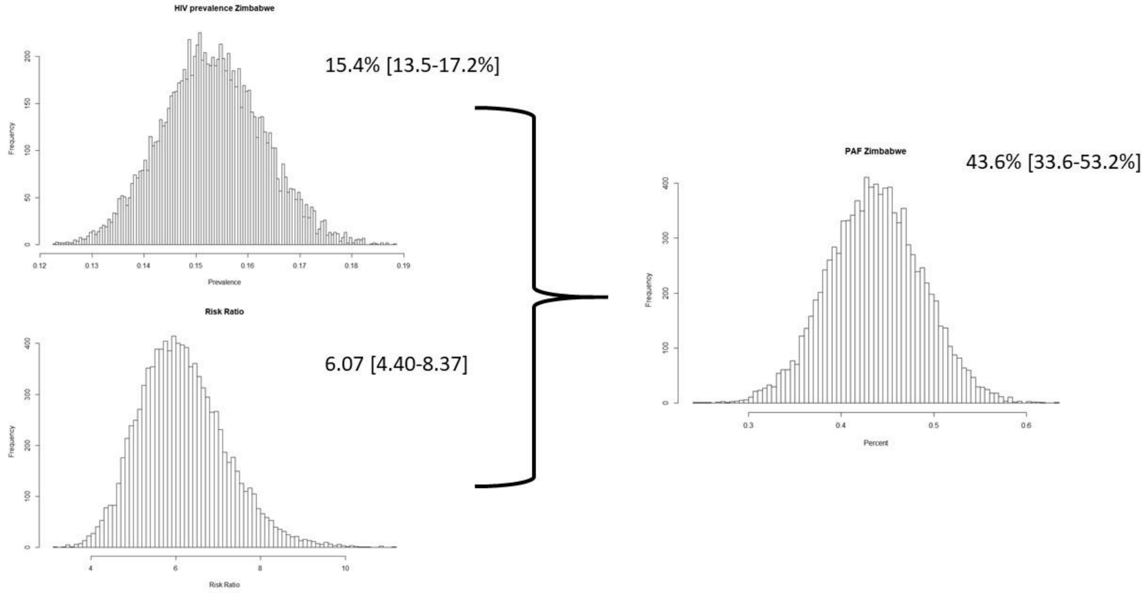


In the next step, we simulated the number of cervical cancer cases for Zimbabwe (bottom left figure) and multiplied it with the simulated PAF for Zimbabwe (left upper figure). The result was the number of cervical cancer cases attributable to HIV for Zimbabwe (right figure).

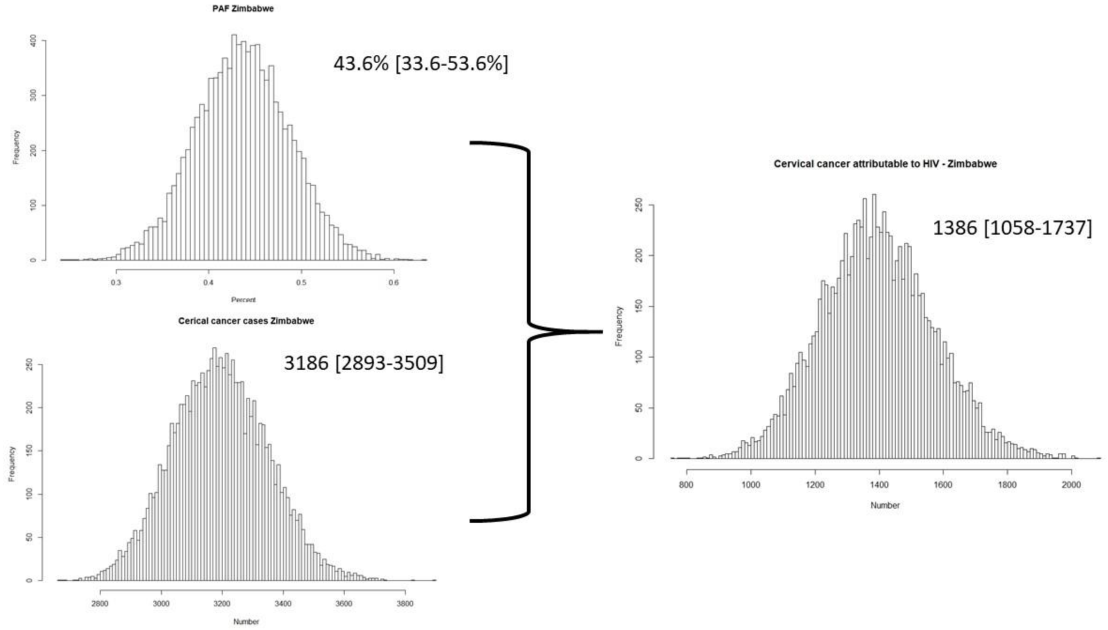


We ran these analyses for all countries and integrated the results into regional and global estimates.

Figure 3. Meta-regression by proportion on antiretroviral therapy

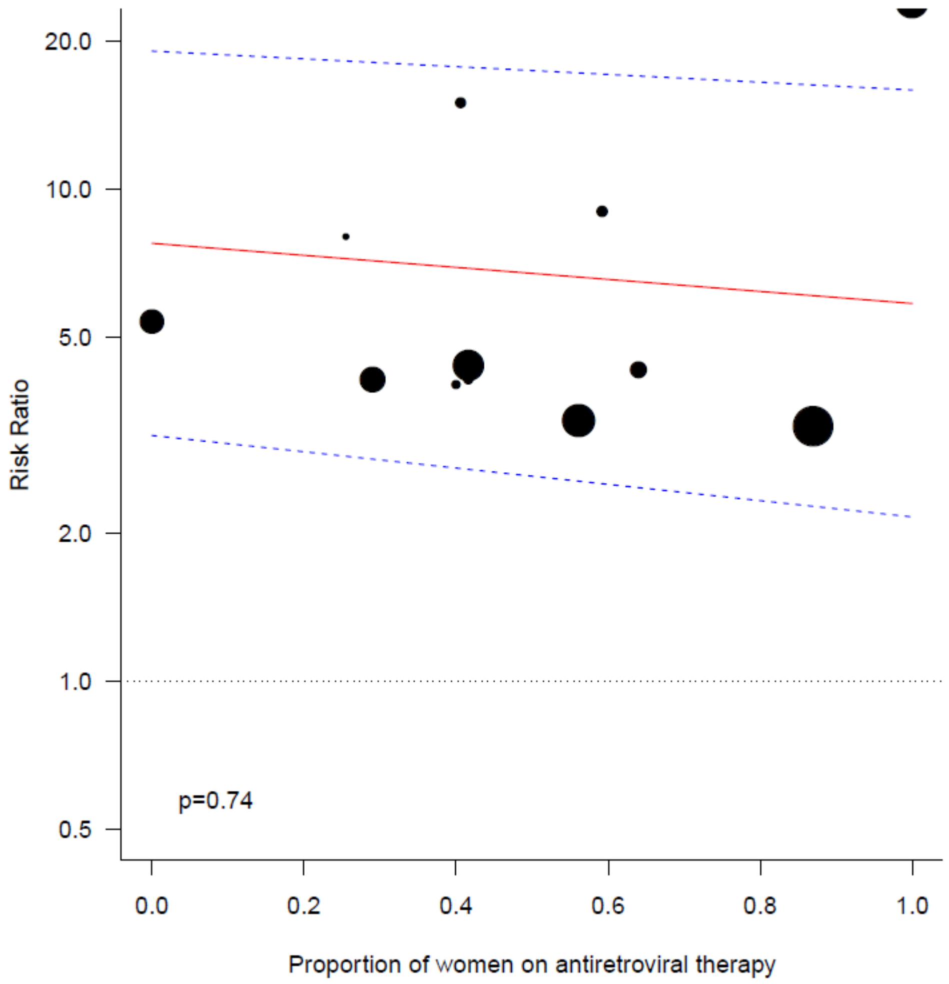

Table 5. Estimated proportion of cervical cancer associated with HIV and PAF to HIV in 2018, by UNAIDS regions

| | Region | | Number of new cervical cancer cases 2018 | HIV prevalence (Females 15 years and older; %) | Proportion of new cervical cancer patients who are living with HIV (%) | Number of new cervical cancer patients living with HIV (2018) | Population attributable fraction for HIV (%) | Number of cervical cancer cases attributable to HIV (2018) |
|---|---|---|---|---|---|---|---|---|
| Global | | | 569 847 [545 771–594 985] | 0·67 [0·59–0·78] | 5·8 [4·6–7·3] | 33 000 [26 000–42 000] | 4·9 [3·6–6·4] | 28 000 [20 000–36 000] |
| UNAIDS region | Asia and the Pacific | AP | 305 603 [285 201–331 333] | 0·13 [0·12–0·17] | 0·9 [0·6–1·4] | 2 900 [2 000–4 200] | 0·8 [0·5–1·2] | 2 400 [1 500–3 600] |
| | Caribbean | CAR | 4 010 [2 775–6 716] | 1·06 [0·91–1·24] | 5·8 [4·3–8·0] | 230 [170–320] | 4·8 [3·3–7·0] | 190 [130–280] |
| | Eastern and southern Africa | ESA | 66 958 [56 541–80 087] | 8·39 [7·47–9·38] | 35·5 [29·1–42·3] | 24 000 [20 000–28 000] | 29·7 [22·7–36·9] | 20 000 [15 000–25 000] |
| | Eastern Europe and central Asia | EECA | 31 855 [29 955–34 124] | 0·45 [0·41–0·49] | 2·7 [1·9–3·7] | 850 [600–1 200] | 2·2 [1·5–3·2] | 710 [470–1 000] |
| | Latin America | LA | 51 703 [47 159–56 814] | 0·25 [0·2–0·31] | 1·5 [1·0–2·2] | 780 [540–1 100] | 1·3 [0·8–1·9] | 650 [420–990] |
| | Middle East and North Africa | MENA | 11 164 [8 902–14 322] | 0·06 [0·04–0·09] | 0·5 [0·3–0·8] | 50 [30–90] | 0·4 [0·3–0·7] | 40 [30–80] |
| | Western and central Africa | WCA | 43 484 [33 335–61 098] | 1·81 [1·45–2·26] | 10·0 [7·0–13·9] | 4 300 [3 000–6 000] | 8·3 [5·5–12·1] | 3 600 [2 400–5 300] |
| | Western and central Europe and North America | WCENA | 52 732 [48 757–57 246] | 0·12 [0·10–0·13] | 0·6 [0·5–0·9] | 340 [240–480] | 0·5 [0·4–0·8] | 280 [190–410] |

Figure 4: Proportion of women with cervical cancer living with HIV, 2018

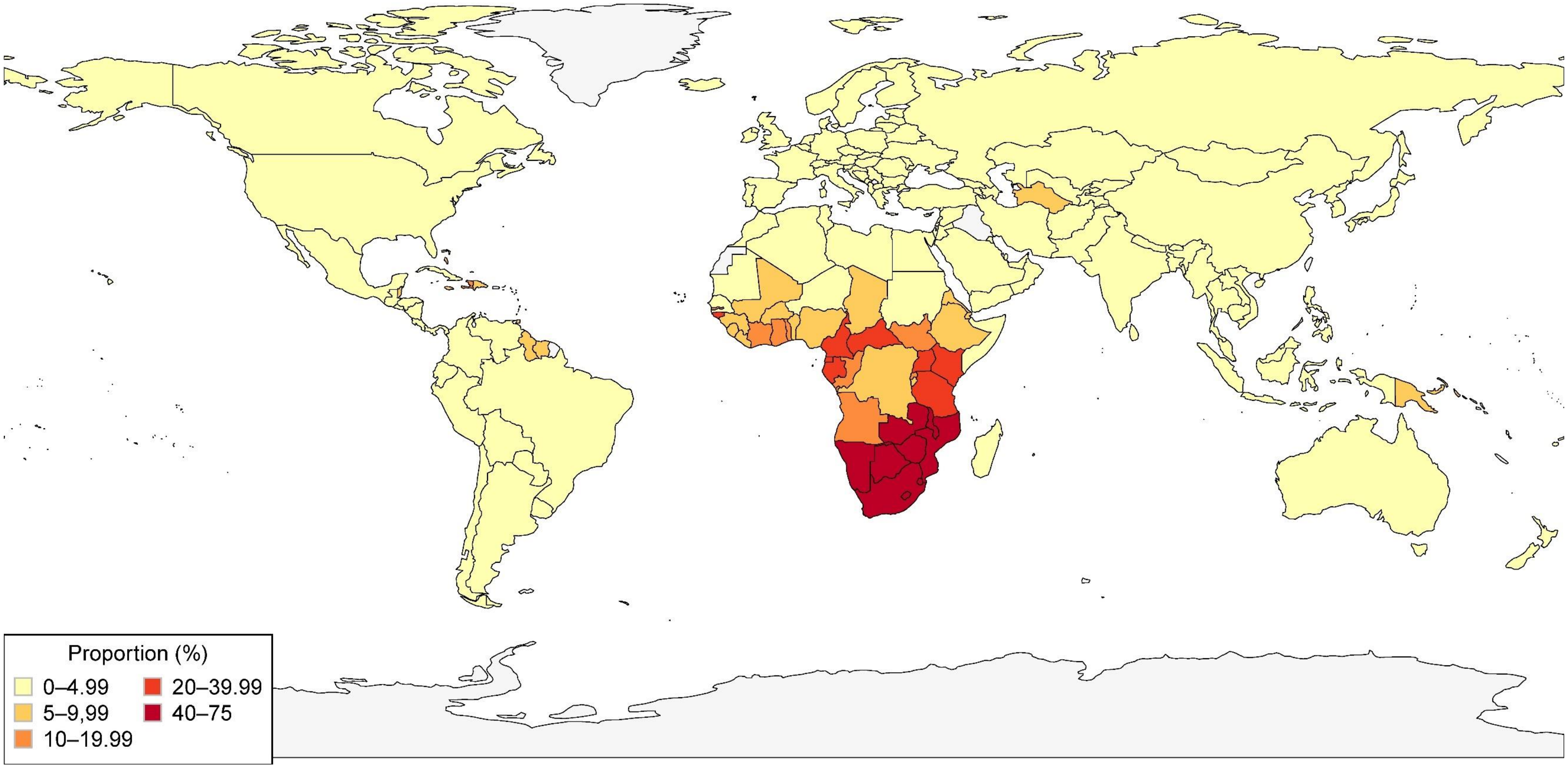

Figure 5: Total number of incident cervical cancer cases among WLHIV

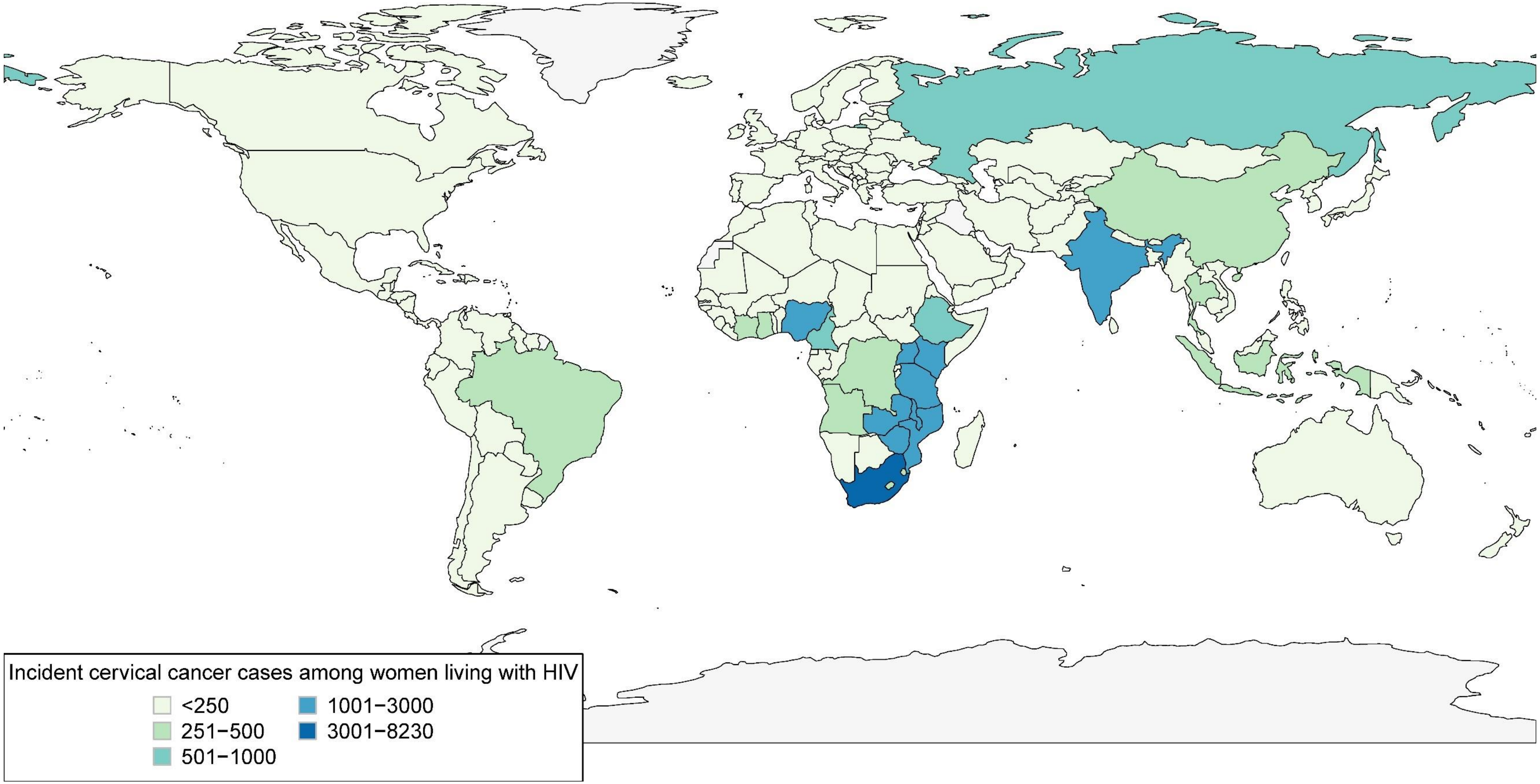

Figure 6: Total number of incident cervical cancer cases attributable to HIV

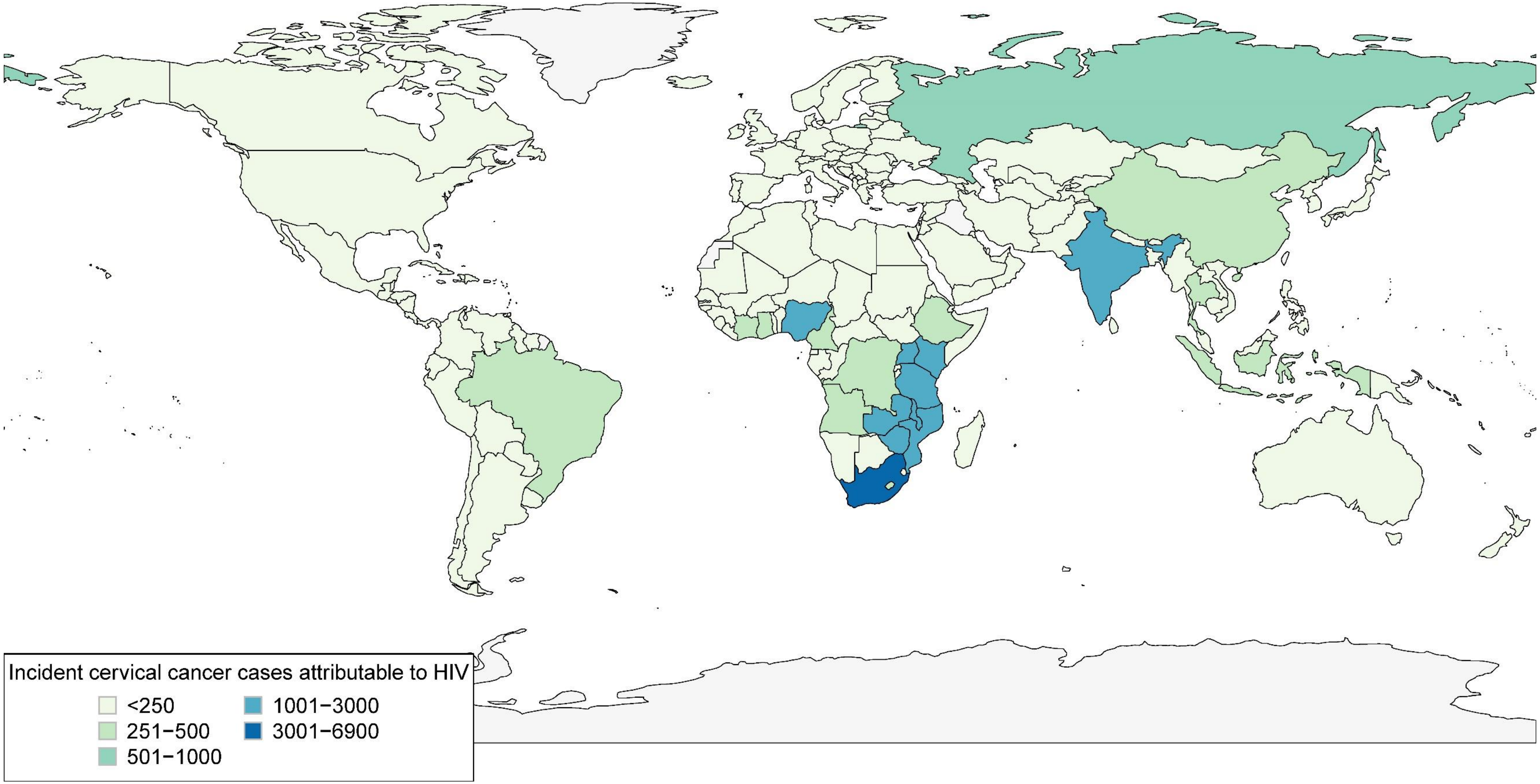

Figure 7: Proportion of women living with HIV among all new cervical cancer patients in 2018, stratified by WHO region

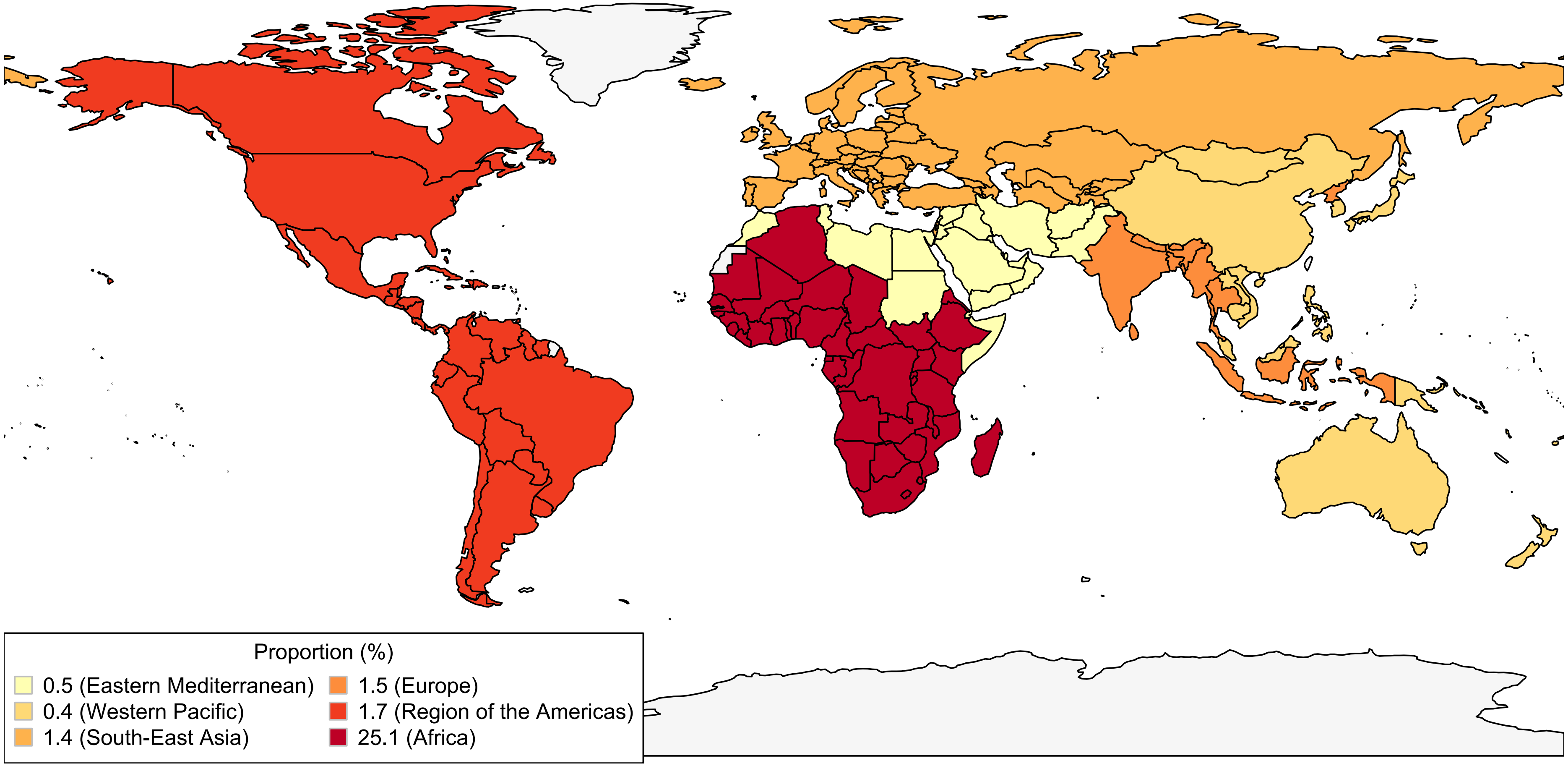

Figure 8: Population attributable fraction for HIV associated with cervical cancer, stratified by WHO region

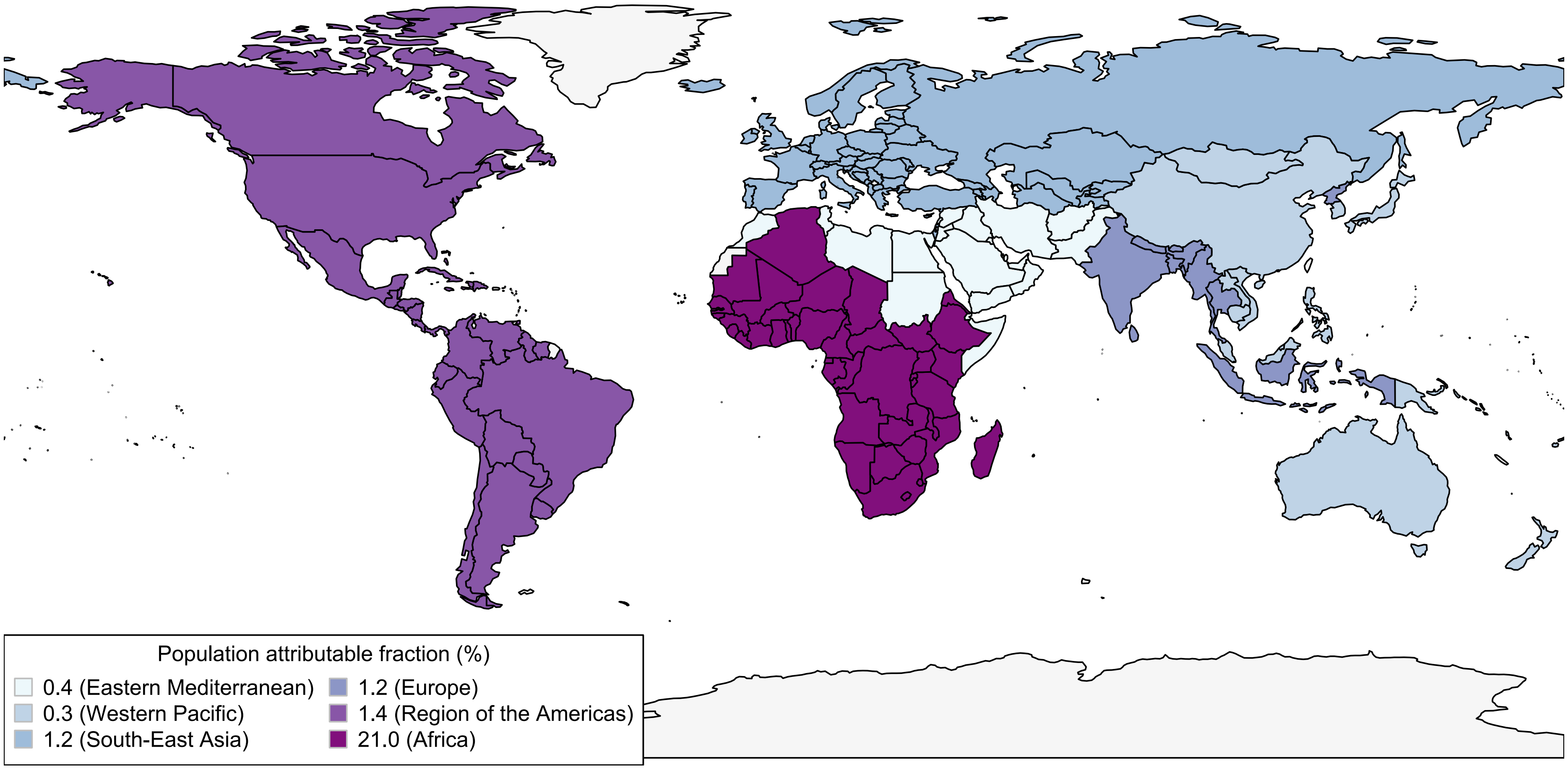

Figure 9: Total number of new cervical cancer cases attributable to HIV in 2018, stratified by WHO region

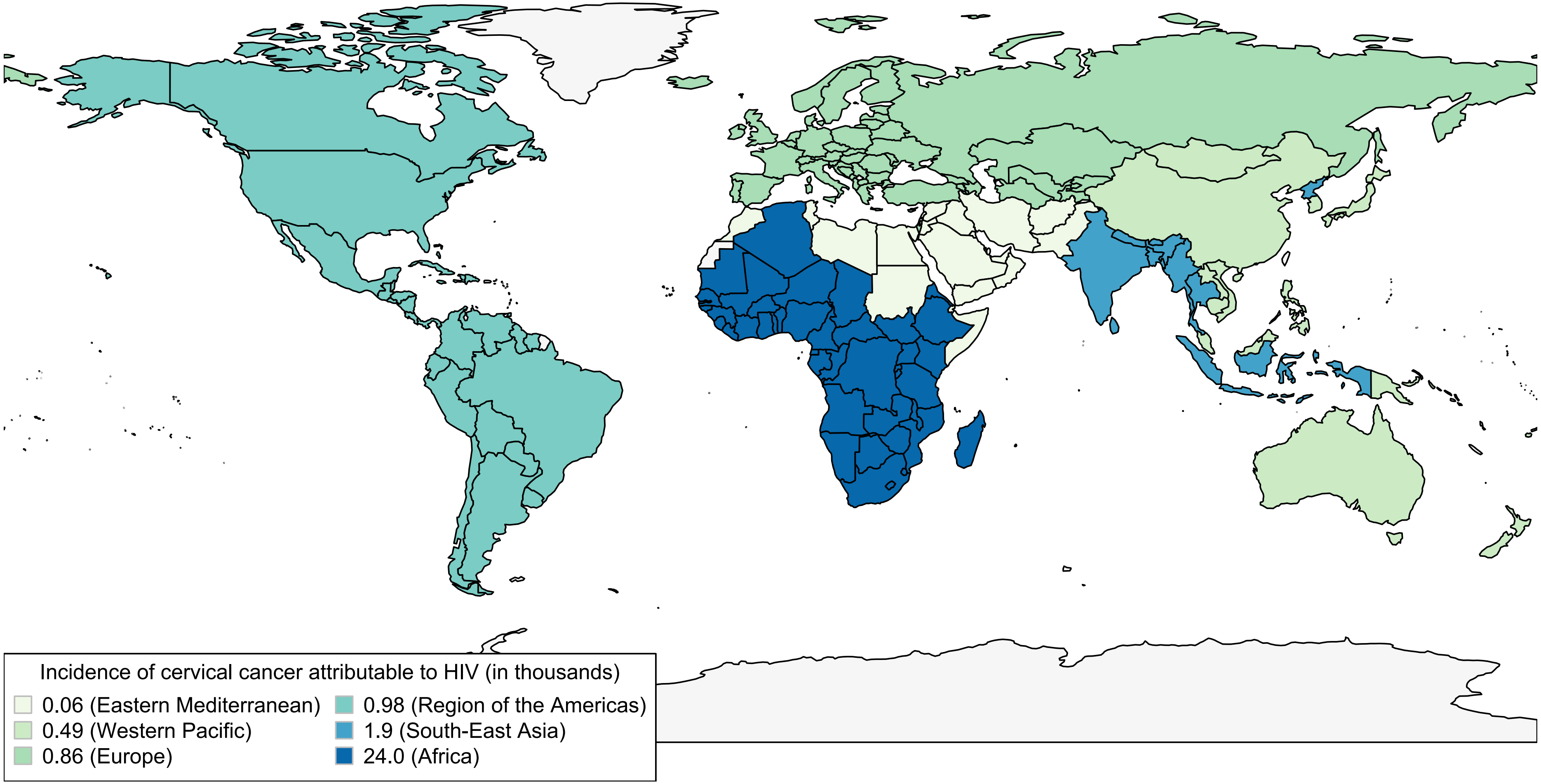

Figure 10: Proportion of women living with HIV among all new cervical cancer patients in 2018, stratified by UNAIDS region

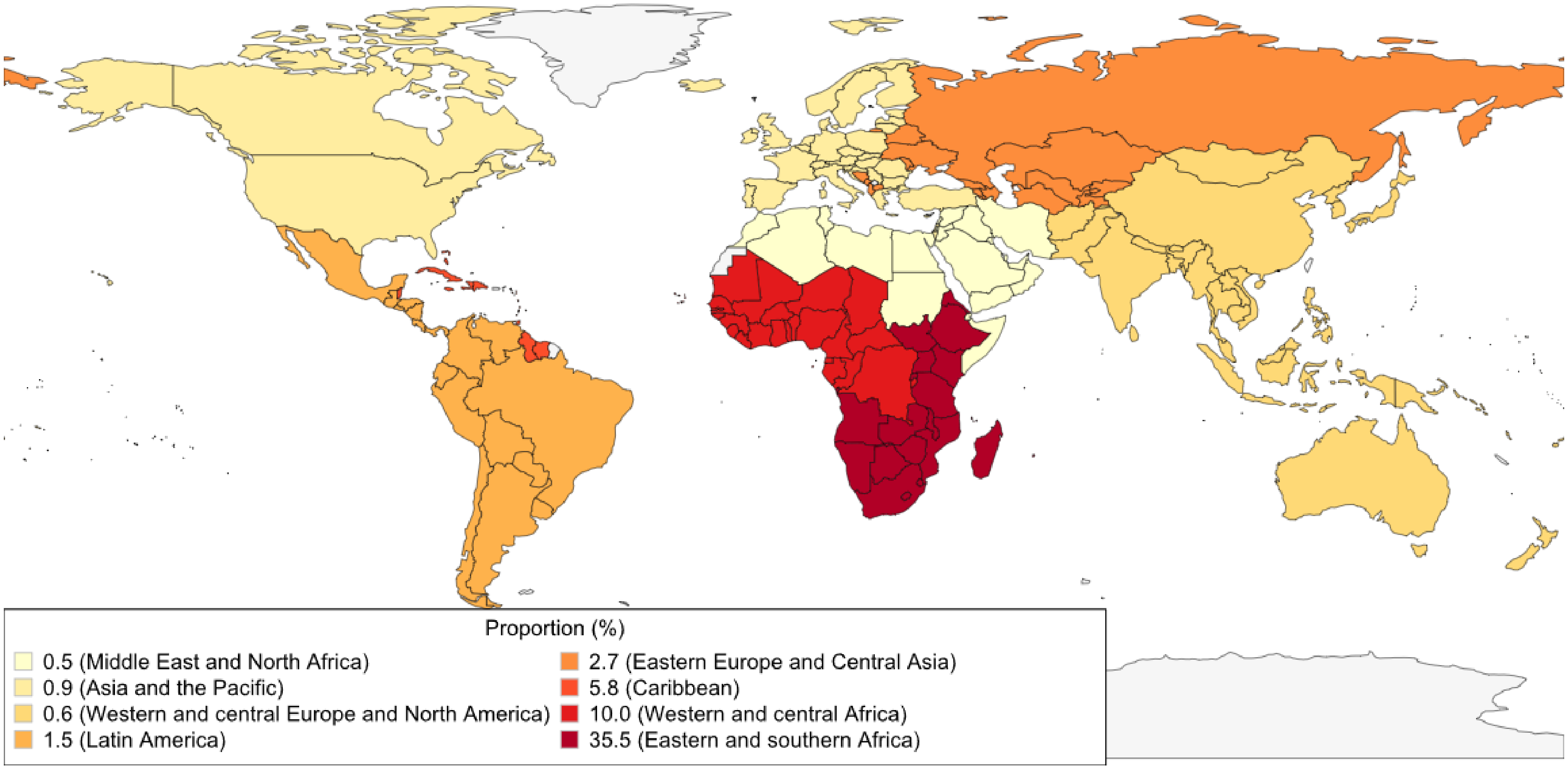

Figure 11: Population attributable fraction for HIV associated with cervical cancer, stratified by UNAIDS region

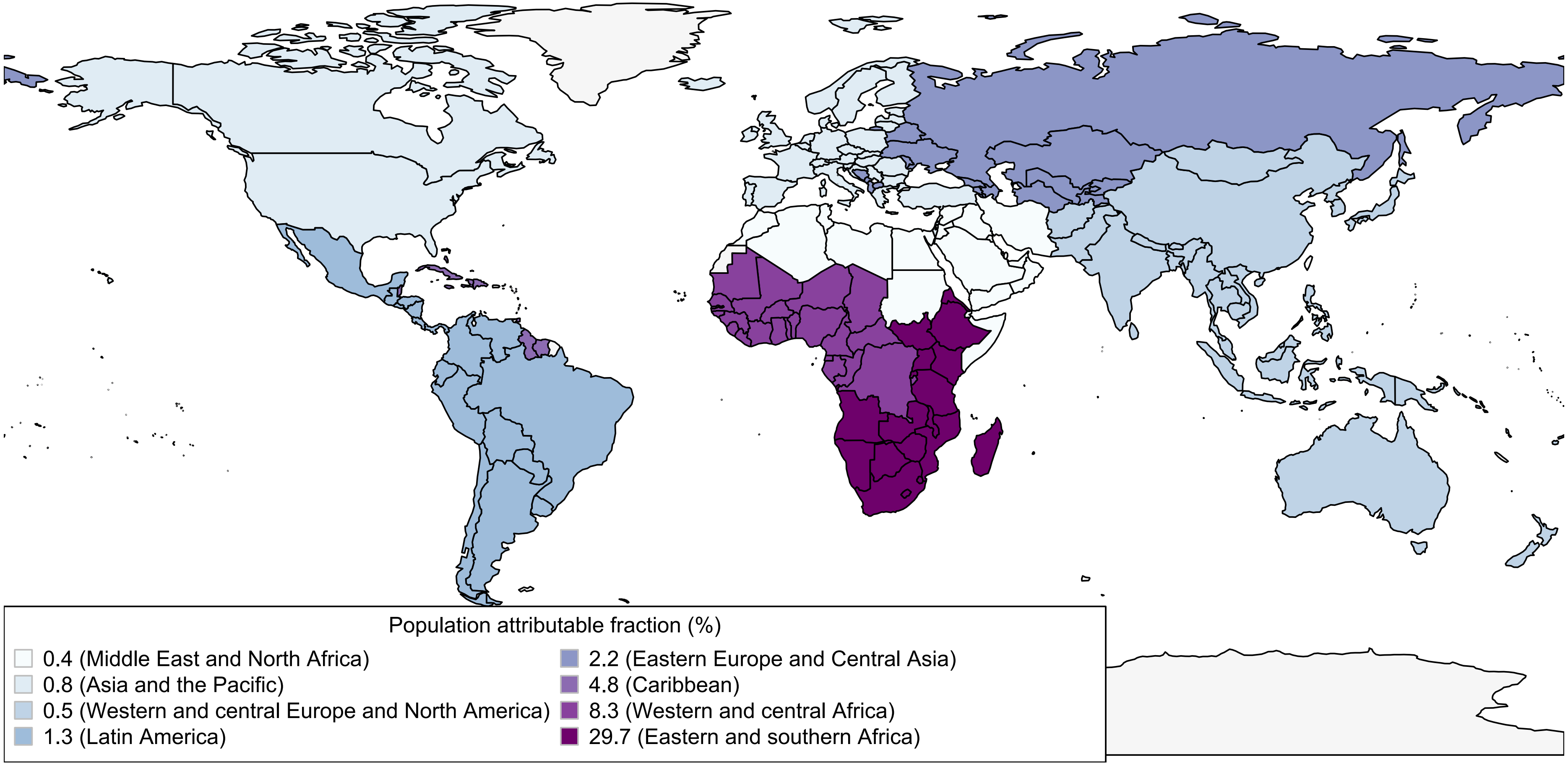

Figure 12: Total number of new cervical cancer cases attributable to HIV in 2018, stratified by UNAIDS regions

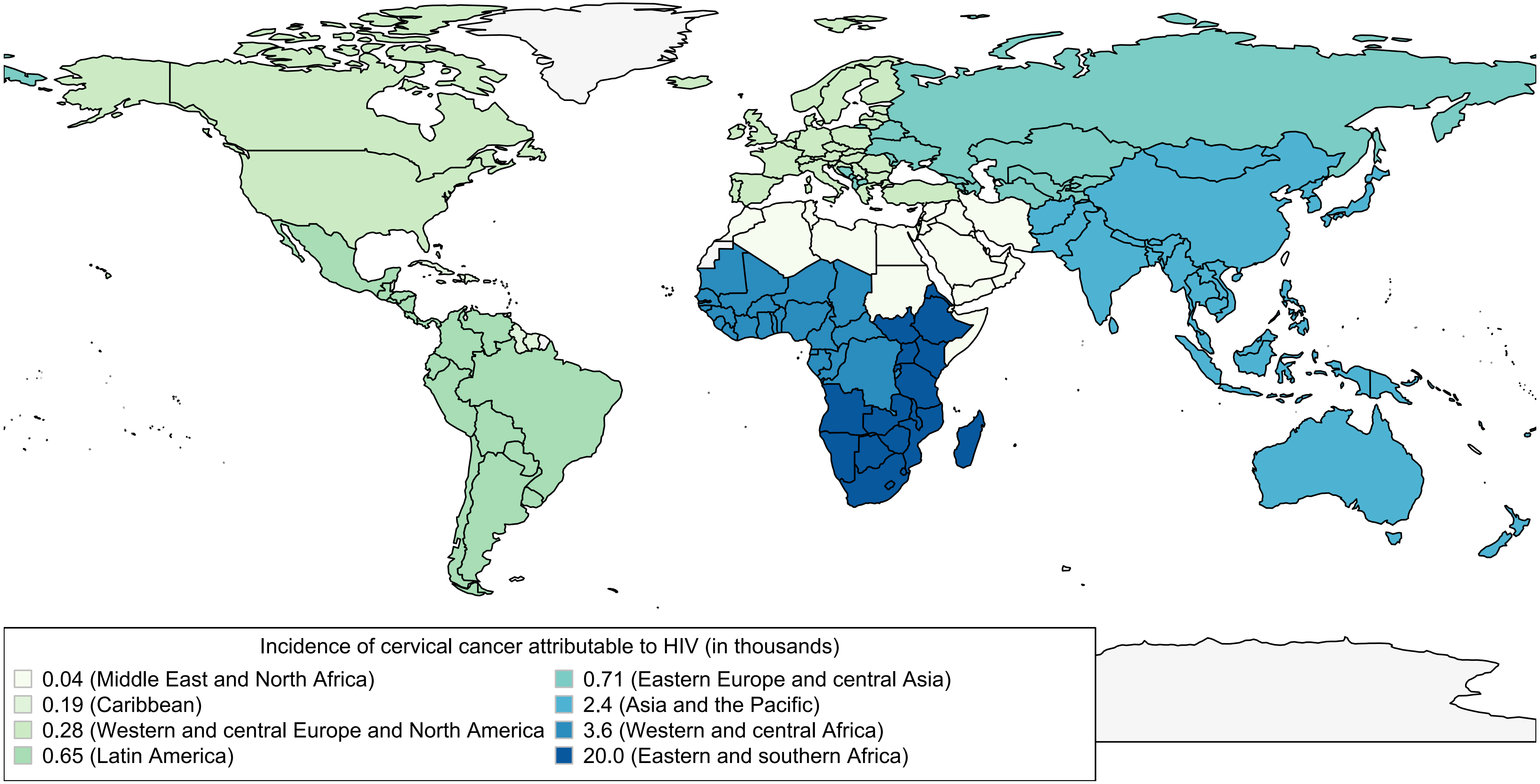

Table 6: Country-level burden of cervical cancer associated with HIV*

| Country | Number of new cervical cancer cases 2018 | HIV prevalence (Females 15 years and older; %) | Proportion of new cervical cancer patients who are living with HIV (%) | Number of cervical cancer patients living with HIV (2018) | Population attributable fraction for HIV (%) | Number of cervical cancer cases attributable to HIV (2018) |
|---|---|---|---|---|---|---|
| Afghanistan | 694 [497–970] | 0.02 [0.01–0.03] | 0.12 [0.06–0.21] | 1 [0–2] | 0.1 [0.05–0.18] | 1 [0–1] |
| Albania | 134 [89–202] | … […–…] | … […–…] | … […–…] | … […–…] | … […–…] |
| Algeria | 1594 [1402–1812] | 0.05 [0.04–0.05] | 0.29 [0.21–0.4] | 5 [3–6] | 0.24 [0.16–0.34] | 4 [2–6] |
| Angola | 2949 [2225–3909] | 2.53 [2.16–2.96] | 13.61 [9.91–18.42] | 401 [262–605] | 11.41 [7.66–16.02] | 335 [208–528] |
| Argentina | 4484 [4068–4943] | 0.26 [0.24–0.28] | 1.54 [1.1–2.13] | 69 [49–96] | 1.28 [0.86–1.86] | 57 [38–84] |
| Armenia | 196 [124–311] | 0.09 [0.08–0.11] | 0.57 [0.39–0.82] | 1 [1–2] | 0.48 [0.31–0.72] | 1 [0–2] |
| Australia | 924 [832–1026] | 0.03 [0.03–0.04] | 0.2 [0.14–0.28] | 2 [1–3] | 0.16 [0.11–0.24] | 2 [1–2] |
| Austria | 390 [314–485] | … […–…] | … […–…] | … […–…] | … […–…] | … […–…] |
| Azerbaijan | 397 [351–449] | … […–…] | … […–…] | … […–…] | … […–…] | … […–…] |
| Bahamas (the) | 29 [16–51] | 1.58 [1.4–1.74] | 8.77 [6.39–11.95] | 3 [1–5] | 7.33 [4.97–10.37] | 2 [1–4] |
| Bahrain | 19 [11–34] | … […–…] | … […–…] | … […–…] | … […–…] | … […–…] |
| Bangladesh | 8068 [5772–11277] | 0.01 [0.01–0.01] | 0.05 [0.03–0.07] | 4 [2–6] | 0.04 [0.03–0.06] | 3 [2–5] |
| Barbados | 38 [22–65] | 0.85 [0.76–0.94] | 4.9 [3.54–6.76] | 2 [1–4] | 4.1 [2.73–5.91] | 2 [1–3] |
| Belarus | 979 [860–1115] | 0.28 [0.23–0.36] | 1.73 [1.17–2.55] | 17 [11–25] | 1.44 [0.92–2.2] | 14 [9–22] |
| Belgium | 640 [570–718] | … […–…] | … […–…] | … […–…] | … […–…] | … […–…] |
| Belize | 46 [27–79] | 1.63 [1.46–1.8] | 9.09 [6.65–12.32] | 4 [2–8] | 7.59 [5.12–10.72] | 3 [2–7] |
| Benin | 783 [651–942] | 1.25 [0.83–1.99] | 7.48 [4.42–11.97] | 58 [33–98] | 6.27 [3.54–10.32] | 49 [27–83] |
| Bhutan | 48 [43–54] | 0.15 [0.09–0.3] | 1.03 [0.48–1.95] | 0 [0–1] | 0.86 [0.39–1.67] | 0 [0–1] |
| Bolivia | 1949 [1732–2193] | 0.18 [0.17–0.19] | 1.08 [0.78–1.5] | 21 [15–30] | 0.9 [0.6–1.3] | 18 [11–26] |
| Bosnia and Herzegovina | 556 [474–653] | 0 [0–0] | 0.02 [0.01–0.03] | 0 [0–0] | 0.02 [0.01–0.02] | 0 [0–0] |
| Botswana | 333 [287–386] | 24.89 [23.01–26.38] | 66.47 [58.59–73.46] | 221 [183–266] | 55.51 [45.35–64.41] | 184 [144–228] |
| Brazil | 16298 [15319–17340] | 0.37 [0.28–0.46] | 2.17 [1.45–3.19] | 352 [236–522] | 1.81 [1.13–2.76] | 294 [184–452] |
| Brunei Darussalam | 52 [33–81] | … […–…] | … […–…] | … […–…] | … […–…] | … […–…] |
| Bulgaria | 1080 [954–1222] | 0.02 [0.02–0.03] | 0.15 [0.1–0.21] | 2 [1–2] | 0.12 [0.08–0.18] | 1 [1–2] |

| | | | | | | |
|---|---|---|---|---|---|---|
| Burkina Faso | 2517 [959–6608] | 0.94 [0.77–1.12] | 5.38 [3.78–7.62] | 136 [48–381] | 4.49 [2.97–6.66] | 113 [40–321] |
| Burundi | 1859 [1214–2846] | 1.41 [1.23–1.65] | 8.07 [5.8–11.1] | 150 [88–257] | 6.74 [4.52–9.69] | 125 [70–220] |
| Cabo Verde | 50 [29–86] | 0.65 [0.55–0.77] | 3.83 [2.69–5.45] | 2 [1–4] | 3.2 [2.11–4.75] | 2 [1–3] |
| Cambodia | 993 [555–1776] | 0.62 [0.55–0.71] | 3.65 [2.61–5.11] | 36 [19–71] | 3.05 [2.02–4.45] | 30 [15–61] |
| Cameroon | 2356 [1878–2955] | 4.69 [4.17–5.09] | 22.66 [17.21–29.1] | 533 [376–752] | 18.96 [13.45–25.45] | 444 [295–647] |
| Canada | 1434 [1255–1638] | … […–…] | … […–…] | … […–…] | … […–…] | … […–…] |
| Central African Republic | 276 [208–366] | 4.21 [3.47–5.21] | 21.28 [15.52–28.4] | 59 [39–88] | 17.76 [12.17–24.63] | 49 [30–76] |
| Chad | 742 [560–984] | 1.45 [1.14–1.79] | 8.12 [5.63–11.58] | 60 [38–94] | 6.78 [4.41–10.06] | 50 [30–82] |
| Chile | 1549 [1301–1845] | 0.16 [0.15–0.17] | 0.96 [0.69–1.34] | 15 [10–22] | 0.8 [0.53–1.16] | 12 [8–19] |
| China | 106430 [103074–109895] | … […–…] | … […–…] | … […–…] | … […–…] | … […–…] |
| Colombia | 3853 [3421–4340] | 0.16 [0.13–0.18] | 0.94 [0.66–1.35] | 36 [25–53] | 0.78 [0.51–1.17] | 30 [19–46] |
| Comoros | 141 [92–216] | 0.02 [0.01–0.04] | 0.13 [0.06–0.25] | 0 [0–0] | 0.11 [0.05–0.21] | 0 [0–0] |
| Democratic Republic of the Congo | 5762 [4347–7638] | 1.11 [0.91–1.32] | 6.3 [4.45–8.98] | 363 [232–571] | 5.26 [3.46–7.79] | 302 [183–492] |
| Congo | 278 [219–352] | 3.53 [2.71–4.68] | 18.43 [12.8–25.7] | 51 [33–77] | 15.33 [10.03–22.27] | 43 [26–67] |
| Costa Rica | 351 [263–468] | 0.15 [0.14–0.17] | 0.91 [0.65–1.27] | 3 [2–5] | 0.76 [0.5–1.1] | 3 [2–4] |
| Côte d'Ivoire | 1789 [1528–2094] | 3.58 [2.84–4.54] | 18.51 [13.04–25.35] | 331 [226–471] | 15.48 [10.29–21.96] | 277 [179–406] |
| Croatia | 266 [208–341] | 0.01 [0.01–0.01] | 0.06 [0.04–0.08] | 0 [0–0] | 0.05 [0.03–0.07] | 0 [0–0] |
| Cuba | 1231 [1065–1423] | 0.12 [0.09–0.15] | 0.69 [0.47–1.03] | 9 [6–13] | 0.58 [0.36–0.89] | 7 [4–11] |
| Cyprus | 45 [30–67] | … […–…] | … […–…] | … […–…] | … […–…] | … […–…] |
| Czech Republic | 813 [711–930] | 0.01 [0.01–0.01] | 0.07 [0.05–0.09] | 1 [0–1] | 0.05 [0.04–0.08] | 0 [0–1] |
| Denmark | 415 [356–484] | 0.07 [0.06–0.08] | 0.4 [0.28–0.57] | 2 [1–2] | 0.34 [0.22–0.49] | 1 [1–2] |
| Djibouti | 52 [34–80] | 1.36 [1.11–1.71] | 7.85 [5.44–11.17] | 4 [2–7] | 6.54 [4.25–9.66] | 3 [2–6] |
| Dominican Republic | 981 [807–1193] | 0.86 [0.68–1.12] | 5.08 [3.45–7.56] | 50 [32–77] | 4.26 [2.71–6.4] | 42 [25–66] |
| Ecuador | 1612 [1462–1778] | 0.21 [0.14–0.34] | 1.35 [0.78–2.24] | 22 [12–37] | 1.12 [0.61–1.92] | 18 [10–31] |
| Egypt | 969 [713–1316] | 0.02 [0.02–0.02] | 0.12 [0.08–0.16] | 1 [1–2] | 0.1 [0.07–0.14] | 1 [1–2] |
| El Salvador | 724 [604–868] | 0.36 [0.29–0.42] | 2.1 [1.45–3.01] | 15 [10–23] | 1.75 [1.13–2.63] | 13 [8–20] |
| Equatorial Guinea | 100 [75–133] | 8.37 [6.72–10.78] | 36.3 [27.39–46.25] | 36 [24–53] | 30.28 [21.43–39.98] | 30 [19–45] |
| Eritrea | 218 [142–334] | 0.87 [0.66–1.18] | 5.15 [3.39–7.64] | 11 [6–20] | 4.3 [2.69–6.62] | 9 [5–17] |
| Estonia | 230 [184–287] | 0.45 [0.4–0.5] | 2.65 [1.88–3.68] | 6 [4–9] | 2.21 [1.47–3.21] | 5 [3–8] |

| | | | | | | |
|---|---|---|---|---|---|---|
| Eswatini | 380 [306–472] | 32.92 [31.29–35.02] | 75.03 [68.24–80.75] | 284 [225–358] | 62.61 [52.81–70.81] | 237 [181–304] |
| Ethiopia | 6294 [5057–7834] | 1.42 [1.09–1.85] | 8.12 [5.46–11.84] | 510 [322–790] | 6.77 [4.32–10.16] | 427 [259–680] |
| Fiji | 124 [102–150] | … […–…] | … […–…] | … […–…] | … […–…] | … […–…] |
| Finland | 182 [144–230] | 0.05 [0.03–0.06] | 0.27 [0.18–0.4] | 0 [0–1] | 0.23 [0.14–0.35] | 0 [0–1] |
| France | 3067 [2836–3317] | 0.16 [0.14–0.18] | 0.97 [0.68–1.37] | 30 [21–42] | 0.81 [0.53–1.18] | 25 [16–37] |
| Gabon | 156 [125–194] | 5.35 [4.29–6.79] | 25.87 [18.78–34.23] | 40 [27–58] | 21.56 [14.95–29.59] | 33 [22–49] |
| Gambia | 184 [107–318] | 2.4 [1.95–3.13] | 13.38 [9.36–18.71] | 24 [13–47] | 11.15 [7.34–16.36] | 20 [10–40] |
| Georgia | 297 [201–439] | 0.17 [0.15–0.2] | 1.03 [0.72–1.45] | 3 [2–5] | 0.86 [0.56–1.26] | 3 [1–4] |
| Germany | 4608 [4332–4901] | 0.05 [0.04–0.05] | 0.29 [0.2–0.4] | 13 [9–19] | 0.24 [0.16–0.35] | 11 [7–16] |
| Ghana | 3151 [2803–3542] | 2.19 [1.86–2.55] | 11.92 [8.56–16.22] | 375 [265–524] | 9.96 [6.74–14.1] | 313 [208–452] |
| Greece | 696 [524–925] | … […–…] | … […–…] | … […–…] | … […–…] | … […–…] |
| Guatemala | 1503 [1300–1737] | 0.33 [0.3–0.36] | 1.95 [1.4–2.7] | 29 [21–42] | 1.63 [1.09–2.35] | 24 [16–36] |
| Guinea | 1810 [1449–2261] | 1.77 [1.49–2.08] | 9.84 [7.02–13.58] | 178 [119–263] | 8.19 [5.45–11.8] | 149 [93–230] |
| Guinea-Bissau | 191 [73–502] | 4.11 [3.58–4.59] | 20.38 [15.34–26.63] | 39 [14–108] | 17.06 [11.94–23.22] | 33 [11–89] |
| Guyana | 124 [99–156] | 1.46 [1.29–1.66] | 8.26 [5.98–11.25] | 10 [7–15] | 6.89 [4.66–9.86] | 9 [5–13] |
| Haiti | 835 [265–2634] | 2.31 [2.07–2.61] | 12.63 [9.28–16.96] | 105 [33–341] | 10.54 [7.17–14.71] | 87 [26–293] |
| Honduras | 804 [666–970] | 0.27 [0.22–0.32] | 1.6 [1.11–2.3] | 13 [9–19] | 1.33 [0.86–2.01] | 11 [7–17] |
| Hungary | 1312 [1125–1531] | 0.01 [0.01–0.01] | 0.08 [0.05–0.11] | 1 [1–1] | 0.06 [0.04–0.09] | 1 [1–1] |
| Iceland | 15 [9–26] | 0.06 [0.06–0.07] | 0.37 [0.27–0.52] | 0 [0–0] | 0.31 [0.21–0.46] | 0 [0–0] |
| India | 96922 [93365–100615] | … […–…] | … […–…] | … […–…] | … […–…] | … […–…] |
| Indonesia | 32469 [30005–35135] | 0.23 [0.2–0.26] | 1.37 [0.97–1.94] | 443 [311–638] | 1.14 [0.75–1.69] | 370 [242–552] |
| Iran | 917 [833–1009] | 0.05 [0.03–0.1] | 0.34 [0.16–0.63] | 3 [1–6] | 0.28 [0.13–0.54] | 3 [1–5] |
| Ireland | 340 [264–437] | 0.13 [0.12–0.14] | 0.78 [0.56–1.09] | 3 [2–4] | 0.65 [0.43–0.94] | 2 [1–4] |
| Israel | 241 [196–297] | 0.07 [0.06–0.08] | 0.43 [0.31–0.6] | 1 [1–2] | 0.36 [0.24–0.52] | 1 [1–1] |
| Italy | 3105 [2903–3321] | 0.15 [0.12–0.16] | 0.86 [0.61–1.21] | 27 [19–38] | 0.72 [0.47–1.06] | 22 [15–33] |
| Jamaica | 486 [324–729] | 1.28 [1.14–1.43] | 7.26 [5.27–10] | 35 [21–58] | 6.06 [4.09–8.68] | 29 [17–51] |
| Japan | 13277 [12653–13932] | 0 [0–0.01] | 0.03 [0.02–0.04] | 4 [3–6] | 0.03 [0.02–0.04] | 3 [2–5] |
| Jordan | 104 [76–142] | 0 [0–0] | 0.01 [0.01–0.02] | 0 [0–0] | 0.01 [0.01–0.01] | 0 [0–0] |
| Kazakhstan | 1729 [1633–1830] | 0.13 [0.12–0.14] | 0.76 [0.55–1.05] | 13 [9–18] | 0.64 [0.42–0.92] | 11 [7–16] |

| | | | | | | |
|---|---|---|---|---|---|---|
| Kenya | 5250 [4615–5972] | 6.21 [5.23–7.48] | 28.87 [21.84–36.99] | 1516 [1116–1998] | 24.17 [17.1–32.29] | 1265 [875–1745] |
| Democratic People's Republic of Korea | 1922 [1648–2242] | … […–…] | … […–…] | … […–…] | … […–…] | … […–…] |
| Republic of Korea | 3348 [3165–3541] | … […–…] | … […–…] | … […–…] | … […–…] | … […–…] |
| Kuwait | 59 [42–83] | 0.04 [0.03–0.04] | 0.23 [0.17–0.33] | 0 [0–0] | 0.19 [0.13–0.28] | 0 [0–0] |
| Kyrgyzstan | 601 [453–798] | 0.12 [0.09–0.17] | 0.77 [0.5–1.19] | 5 [3–8] | 0.64 [0.39–1.02] | 4 [2–7] |
| Lao People's Democratic Republic | 320 [179–572] | 0.21 [0.19–0.23] | 1.26 [0.89–1.77] | 4 [2–8] | 1.05 [0.7–1.53] | 3 [2–7] |
| Latvia | 339 [260–442] | 0.18 [0.17–0.2] | 1.1 [0.79–1.54] | 4 [2–6] | 0.92 [0.61–1.35] | 3 [2–5] |
| Lebanon | 192 [154–239] | 0.02 [0.01–0.02] | 0.1 [0.07–0.14] | 0 [0–0] | 0.08 [0.06–0.12] | 0 [0–0] |
| Lesotho | 477 [366–621] | 27.03 [25.4–28.93] | 69.3 [61.65–76.01] | 330 [248–440] | 57.85 [47.71–66.57] | 275 [200–372] |
| Liberia | 548 [209–1439] | 1.53 [1.4–1.68] | 8.63 [6.32–11.71] | 47 [17–130] | 7.2 [4.92–10.16] | 40 [14–108] |
| Libya | 319 [224–455] | 0.11 [0.1–0.12] | 0.67 [0.48–0.94] | 2 [1–3] | 0.56 [0.37–0.82] | 2 [1–3] |
| Lithuania | 431 [370–502] | … […–…] | … […–…] | … […–…] | … […–…] | … […–…] |
| Luxembourg | 25 [11–56] | 0.13 [0.12–0.15] | 0.8 [0.57–1.12] | 0 [0–0] | 0.67 [0.44–0.98] | 0 [0–0] |
| Madagascar | 4353 [2844–6663] | 0.15 [0.11–0.21] | 0.94 [0.6–1.43] | 41 [22–75] | 0.78 [0.48–1.24] | 34 [18–64] |
| Malawi | 4163 [3500–4952] | 11.21 [10.3–11.95] | 43.04 [35.12–51.42] | 1787 [1373–2298] | 35.94 [27.18–44.86] | 1492 [1082–1999] |
| Malaysia | 1682 [1551–1825] | 0.12 [0.1–0.13] | 0.71 [0.5–0.99] | 12 [8–17] | 0.59 [0.39–0.87] | 10 [7–15] |
| Maldives | 41 [29–57] | … […–…] | … […–…] | … […–…] | … […–…] | … […–…] |
| Mali | 2206 [1924–2530] | 1.69 [1.35–2.11] | 9.49 [6.6–13.39] | 209 [142–302] | 7.93 [5.15–11.63] | 175 [111–262] |
| Malta | 11 [6–21] | … […–…] | … […–…] | … […–…] | … […–…] | … […–…] |
| Mauritania | 481 [183–1263] | 0.17 [0.14–0.21] | 1.04 [0.71–1.51] | 5 [2–14] | 0.87 [0.55–1.31] | 4 [1–12] |
| Mauritius | 120 [85–169] | 0.69 [0.57–0.83] | 4.03 [2.8–5.74] | 5 [3–8] | 3.36 [2.2–4.97] | 4 [2–7] |
| Mexico | 7869 [7469–8290] | 0.07 [0.06–0.09] | 0.45 [0.32–0.64] | 35 [25–50] | 0.38 [0.25–0.55] | 30 [19–44] |
| Mongolia | 370 [327–419] | 0.01 [0.01–0.01] | 0.06 [0.04–0.08] | 0 [0–0] | 0.05 [0.03–0.07] | 0 [0–0] |
| Montenegro | 54 [29–101] | 0.02 [0.02–0.02] | 0.11 [0.08–0.16] | 0 [0–0] | 0.09 [0.06–0.14] | 0 [0–0] |
| Morocco | 3388 [3004–3821] | 0.06 [0.05–0.08] | 0.4 [0.27–0.6] | 14 [9–20] | 0.33 [0.21–0.51] | 11 [7–18] |
| Mozambique | 4291 [3768–4887] | 14.23 [11.47–17.42] | 50.21 [39.87–60.14] | 2151 [1669–2690] | 41.91 [31.43–52.23] | 1793 [1299–2330] |
| Myanmar | 6472 [3620–11572] | 0.42 [0.38–0.48] | 2.53 [1.81–3.53] | 164 [84–317] | 2.12 [1.4–3.06] | 136 [67–269] |
| Namibia | 236 [191–292] | 14.19 [13.3–15.28] | 50.22 [41.86–58.47] | 118 [89–154] | 41.9 [32.38–51.19] | 98 [72–132] |
| Nepal | 2942 [1892–4574] | 0.11 [0.09–0.12] | 0.64 [0.46–0.91] | 19 [11–33] | 0.54 [0.36–0.79] | 16 [9–29] |

| | | | | | | |
|---|---|---|---|---|---|---|
| Netherlands | 670 [584–769] | … […–…] | … […–…] | … […–…] | … […–…] | … […–…] |
| New Zealand | 190 [145–250] | 0.03 [0.03–0.03] | 0.18 [0.13–0.25] | 0 [0–1] | 0.15 [0.1–0.22] | 0 [0–0] |
| Nicaragua | 677 [533–859] | 0.13 [0.11–0.17] | 0.82 [0.55–1.19] | 6 [3–9] | 0.68 [0.43–1.03] | 5 [3–7] |
| Niger | 543 [336–879] | 0.31 [0.26–0.37] | 1.85 [1.29–2.66] | 10 [5–19] | 1.55 [1–2.3] | 8 [4–16] |
| Nigeria | 14943 [12946–17249] | 1.72 [1.29–2.29] | 9.67 [6.42–14.03] | 1445 [941–2154] | 8.07 [5.03–12.16] | 1203 [737–1868] |
| Norway | 361 [305–428] | 0.07 [0.07–0.08] | 0.44 [0.31–0.61] | 2 [1–2] | 0.37 [0.24–0.53] | 1 [1–2] |
| Oman | 77 [52–114] | 0.09 [0.08–0.1] | 0.56 [0.4–0.78] | 0 [0–1] | 0.46 [0.31–0.68] | 0 [0–1] |
| Pakistan | 5601 [4538–6913] | 0.07 [0.07–0.08] | 0.45 [0.32–0.63] | 25 [17–37] | 0.38 [0.24–0.55] | 21 [13–32] |
| Panama | 432 [334–559] | 0.52 [0.47–0.57] | 3.08 [2.22–4.22] | 13 [9–20] | 2.57 [1.72–3.69] | 11 [7–17] |
| Papua New Guinea | 1024 [890–1178] | 0.91 [0.83–1] | 5.28 [3.83–7.26] | 54 [38–77] | 4.4 [2.95–6.31] | 45 [29–67] |
| Paraguay | 1033 [842–1267] | 0.27 [0.2–0.37] | 1.67 [1.08–2.55] | 17 [11–28] | 1.39 [0.85–2.2] | 14 [8–24] |
| Peru | 4103 [3776–4459] | 0.15 [0.11–0.21] | 0.94 [0.59–1.45] | 39 [24–60] | 0.78 [0.46–1.27] | 32 [19–52] |
| Philippines | 7190 [6784–7620] | 0.01 [0.01–0.01] | 0.08 [0.05–0.11] | 6 [4–8] | 0.06 [0.04–0.1] | 5 [3–7] |
| Poland | 3220 [2994–3463] | … […–…] | … […–…] | … […–…] | … […–…] | … […–…] |
| Portugal | 750 [684–822] | 0.25 [0.22–0.28] | 1.49 [1.06–2.08] | 11 [8–16] | 1.24 [0.82–1.8] | 9 [6–14] |
| Qatar | 19 [13–27] | … […–…] | … […–…] | … […–…] | … […–…] | … […–…] |
| Republic of Moldova | 639 [509–802] | 0.39 [0.31–0.5] | 2.37 [1.6–3.49] | 15 [10–24] | 1.98 [1.26–3.02] | 13 [8–20] |
| Romania | 3308 [3156–3468] | 0.08 [0.07–0.08] | 0.46 [0.33–0.64] | 15 [11–21] | 0.38 [0.25–0.55] | 13 [8–18] |
| Russian Federation | 18164 [17728–18611] | … […–…] | … […–…] | … […–…] | … […–…] | … […–…] |
| Rwanda | 1304 [969–1756] | 3.32 [2.95–3.69] | 17.16 [12.82–22.63] | 224 [149–335] | 14.33 [9.9–19.74] | 187 [117–290] |
| Saudi Arabia | 316 [186–537] | … […–…] | … […–…] | … […–…] | … […–…] | … […–…] |
| Senegal | 1876 [715–4925] | 0.52 [0.45–0.59] | 3.05 [2.17–4.29] | 58 [20–159] | 2.55 [1.69–3.73] | 48 [17–134] |
| Serbia | 1327 [1205–1461] | 0.01 [0.01–0.02] | 0.07 [0.05–0.11] | 1 [1–2] | 0.06 [0.04–0.1] | 1 [0–1] |
| Sierra Leone | 299 [275–325] | 1.69 [1.35–2.06] | 9.37 [6.51–13.12] | 28 [19–40] | 7.83 [5.15–11.5] | 23 [15–35] |
| Singapore | 429 [282–652] | 0.04 [0.04–0.05] | 0.26 [0.19–0.37] | 1 [1–2] | 0.22 [0.14–0.32] | 1 [1–2] |
| Slovakia | 692 [635–755] | 0.01 [0.01–0.01] | 0.05 [0.03–0.08] | 0 [0–1] | 0.04 [0.02–0.07] | 0 [0–0] |
| Slovenia | 110 [74–164] | 0.01 [0.01–0.02] | 0.07 [0.04–0.1] | 0 [0–0] | 0.06 [0.03–0.09] | 0 [0–0] |
| Somalia | 989 [646–1514] | 0.14 [0.1–0.2] | 0.86 [0.54–1.34] | 8 [5–16] | 0.72 [0.43–1.18] | 7 [4–14] |
| South Africa | 12983 [12221–13793] | 22.39 [20.63–23.89] | 63.35 [55.18–70.74] | 8223 [7087–9316] | 52.93 [42.75–61.94] | 6866 [5511–8147] |

| | | | | | | |
|---|---|---|---|---|---|---|
| South Sudan | 1101 [719–1685] | 2.75 [2.01–3.46] | 14.28 [9.66–20.1] | 157 [88–272] | 11.83 [7.69–17.52] | 130 [72–233] |
| Spain | 1942 [1751–2154] | 0.13 [0.12–0.15] | 0.81 [0.58–1.12] | 16 [11–22] | 0.67 [0.45–0.98] | 13 [9–19] |
| Sri Lanka | 1136 [1051–1228] | 0.01 [0.01–0.01] | 0.07 [0.05–0.1] | 1 [1–1] | 0.06 [0.04–0.09] | 1 [0–1] |
| Sudan | 1084 [751–1565] | 0.21 [0.09–0.39] | 1.23 [0.52–2.55] | 13 [5–30] | 1.01 [0.42–2.15] | 11 [4–26] |
| Suriname | 85 [57–126] | 1.13 [0.75–1.63] | 6.48 [3.93–10.17] | 5 [3–10] | 5.39 [3.15–8.81] | 5 [2–9] |
| Sweden | 558 [496–628] | … […–…] | … […–…] | … […–…] | … […–…] | … […–…] |
| Switzerland | 258 [225–296] | … […–…] | … […–…] | … […–…] | … […–…] | … […–…] |
| Syrian Arab Republic | 259 [123–545] | 0 [0–0] | 0.02 [0.01–0.03] | 0 [0–0] | 0.02 [0.01–0.02] | 0 [0–0] |
| Tajikistan | 220 [157–308] | 0.11 [0.1–0.14] | 0.69 [0.48–1] | 2 [1–3] | 0.58 [0.37–0.87] | 1 [1–2] |
| Tanzania | 9772 [8065–11841] | 5.7 [5.17–6.21] | 26.73 [20.6–33.79] | 2611 [1900–3520] | 22.29 [16.01–29.4] | 2177 [1478–3067] |
| Thailand | 8622 [8246–9015] | 0.72 [0.62–0.83] | 4.19 [2.99–5.89] | 361 [257–509] | 3.51 [2.32–5.1] | 303 [199–440] |
| North Macedonia | 151 [107–214] | 0.01 [0.01–0.01] | 0.04 [0.03–0.05] | 0 [0–0] | 0.03 [0.02–0.05] | 0 [0–0] |
| Timor-Leste | 50 [28–89] | … […–…] | … […–…] | … […–…] | … […–…] | … […–…] |
| Togo | 568 [516–625] | 2.83 [2.62–3.06] | 15.02 [11.24–19.9] | 85 [63–115] | 12.55 [8.69–17.28] | 71 [49–99] |
| Trinidad and Tobago | 140 [86–229] | … […–…] | … […–…] | … […–…] | … […–…] | … […–…] |
| Tunisia | 285 [229–355] | 0.02 [0.01–0.03] | 0.12 [0.07–0.2] | 0 [0–1] | 0.1 [0.06–0.17] | 0 [0–1] |
| Turkey | 2356 [2125–2612] | … […–…] | … […–…] | … […–…] | … […–…] | … […–…] |
| Turkmenistan | 397 [298–528] | … […–…] | … […–…] | … […–…] | … […–…] | … […–…] |
| Uganda | 6413 [5607–7334] | 7.14 [6.67–7.8] | 32.05 [25.2–39.6] | 2052 [1564–2642] | 26.74 [19.49–34.68] | 1715 [1222–2302] |
| Ukraine | 5733 [5450–6030] | 0.42 [0.39–0.46] | 2.52 [1.81–3.47] | 144 [103–200] | 2.1 [1.41–3.02] | 120 [81–173] |
| United Arab Emirates | 108 [89–132] | … […–…] | … […–…] | … […–…] | … […–…] | … […–…] |
| United Kingdom | 3430 [3237–3634] | … […–…] | … […–…] | … […–…] | … […–…] | … […–…] |
| USA | 14065 [13724–14414] | … […–…] | … […–…] | … […–…] | … […–…] | … […–…] |
| Uruguay | 288 [231–359] | 0.28 [0.21–0.37] | 1.67 [1.08–2.54] | 5 [3–8] | 1.39 [0.85–2.2] | 4 [2–7] |
| Uzbekistan | 1608 [1492–1733] | 0.13 [0.12–0.14] | 0.77 [0.55–1.07] | 12 [9–17] | 0.64 [0.43–0.93] | 10 [7–15] |
| Venezuela | 4174 [3838–4539] | … […–…] | … […–…] | … […–…] | … […–…] | … […–…] |
| Viet Nam | 4177 [3834–4551] | 0.2 [0.17–0.22] | 1.17 [0.83–1.65] | 49 [34–70] | 0.98 [0.65–1.44] | 41 [27–61] |
| Yemen | 170 [115–251] | 0.03 [0.02–0.04] | 0.17 [0.09–0.28] | 0 [0–1] | 0.14 [0.07–0.25] | 0 [0–0] |
| Zambia | 2994 [2589–3462] | 13.85 [12.27–15.73] | 49.57 [40.75–58.15] | 1481 [1165–1848] | 41.32 [31.68–50.9] | 1234 [913–1586] |

| | | | | | | |
|---|---|---|---|---|---|---|
| Zimbabwe | 3186 [2893–3509] | 15.36 [13.47–17.22] | 52.19 [43.47–60.94] | 1660 [1350–1996] | 43.58 [33.62–53.2] | 1386 [1058–1737] |

* Countries with suppressed UNAIDS HIV estimates were labelled … […–…] for all estimates derived from HIV prevalence.

Figure 13. Estimated proportion and population attributable fraction for HIV associated cervical cancer, ranked by burden proportion from highest to lowest, according to UNAIDS region

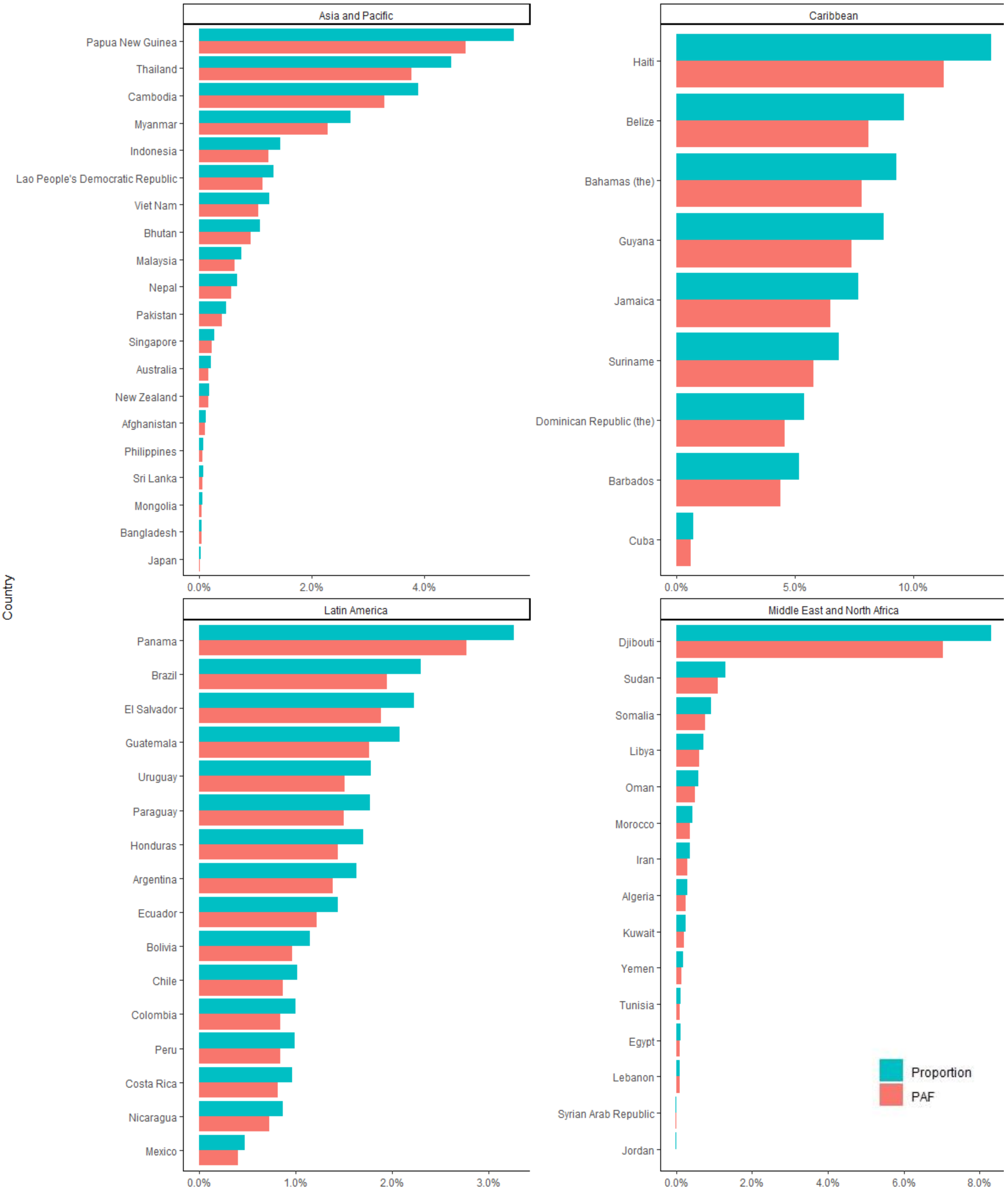

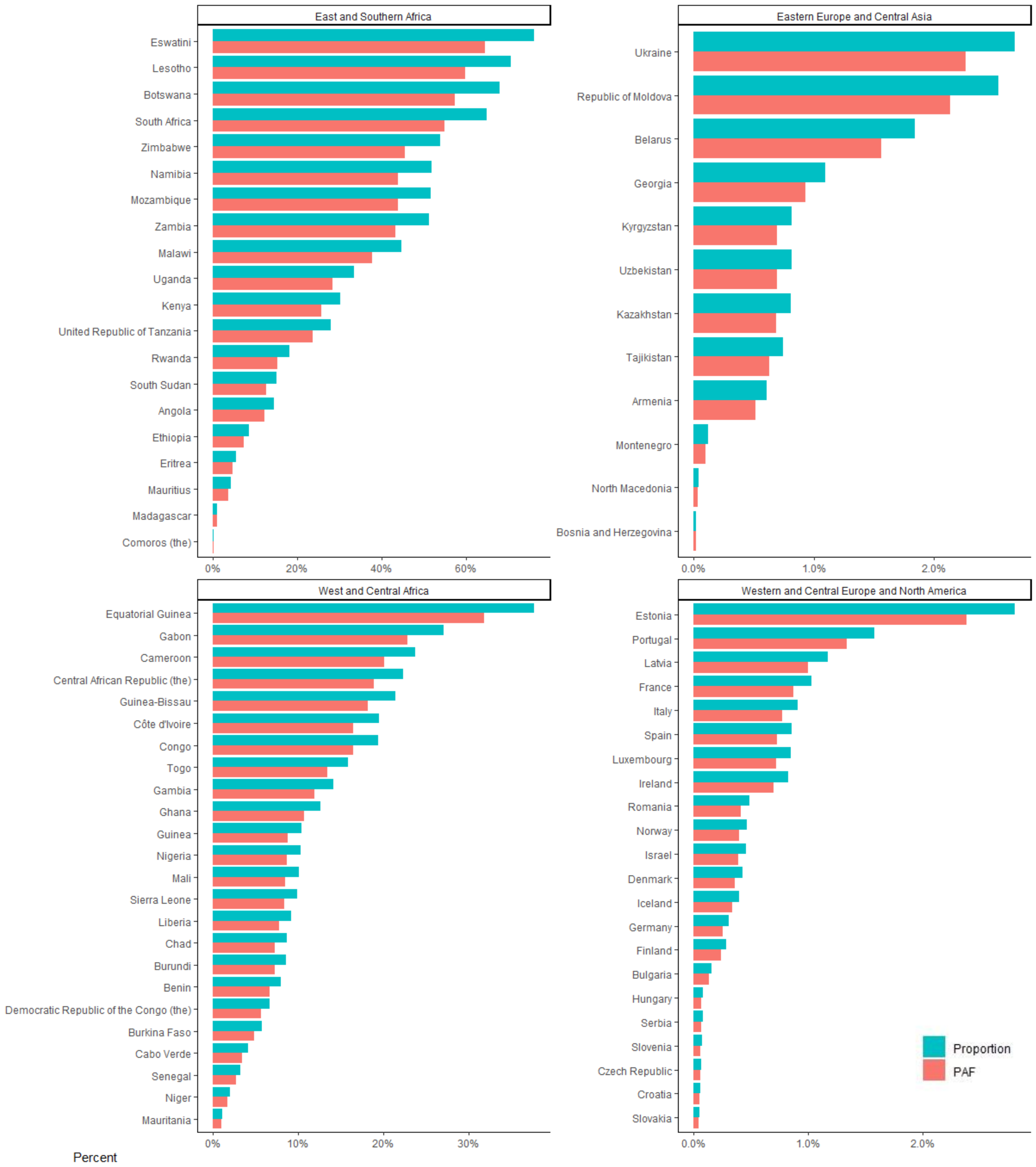


Proportion: Proportion of cervical cancer patients living with HIV
PAF: Proportion of cervical cancer cases attributable to HIV

Figure 14. Estimated population attributable fraction for cervical cancer and HIV, ranked from highest to lowest proportion, restricted to 50 highest ranked countries (by UNAIDS region)⸸

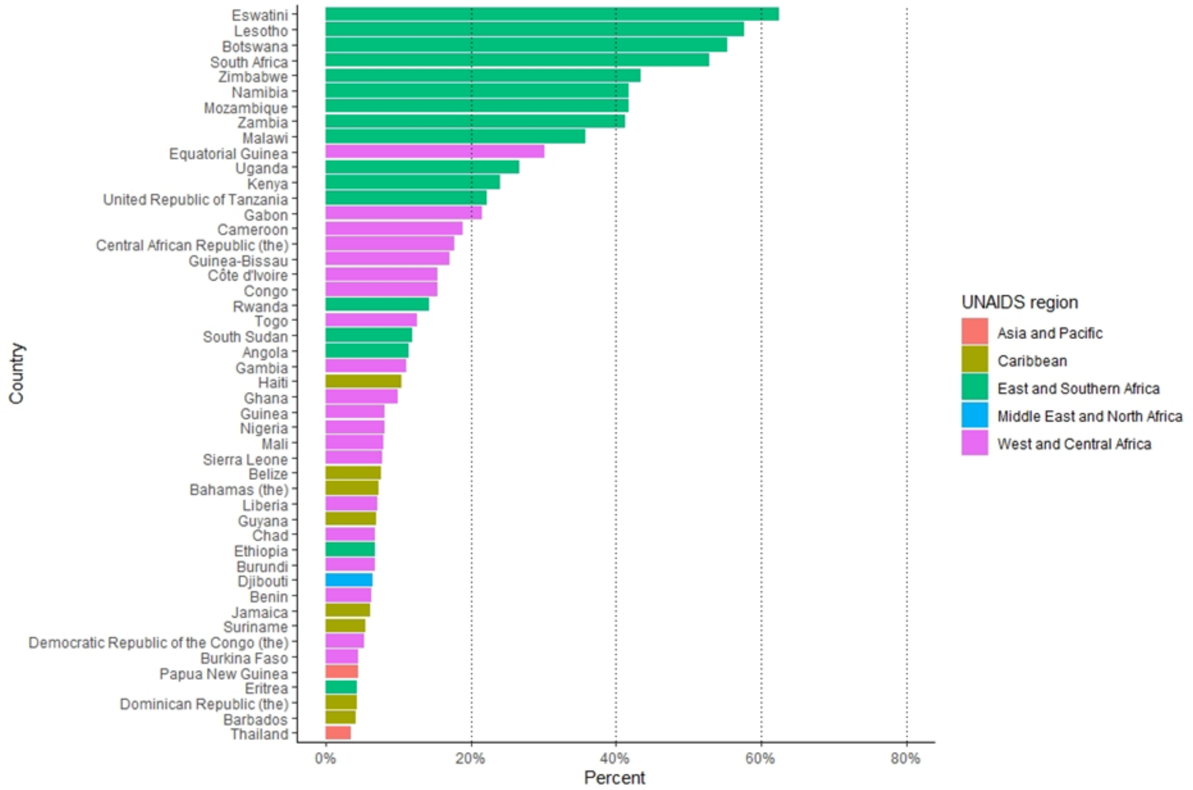


⸸ Turkmenistan (Eastern Europe and Central Asia; 5–9.99%), and Trinidad and Tobago (Caribbean; <5%) are also among the top 50 countries; they are not displayed because their HIV estimates are not published by UNAIDS

Figure 15. Countries with >250 cervical cancer cases attributable to HIV in 2018, by UNAIDS region‡

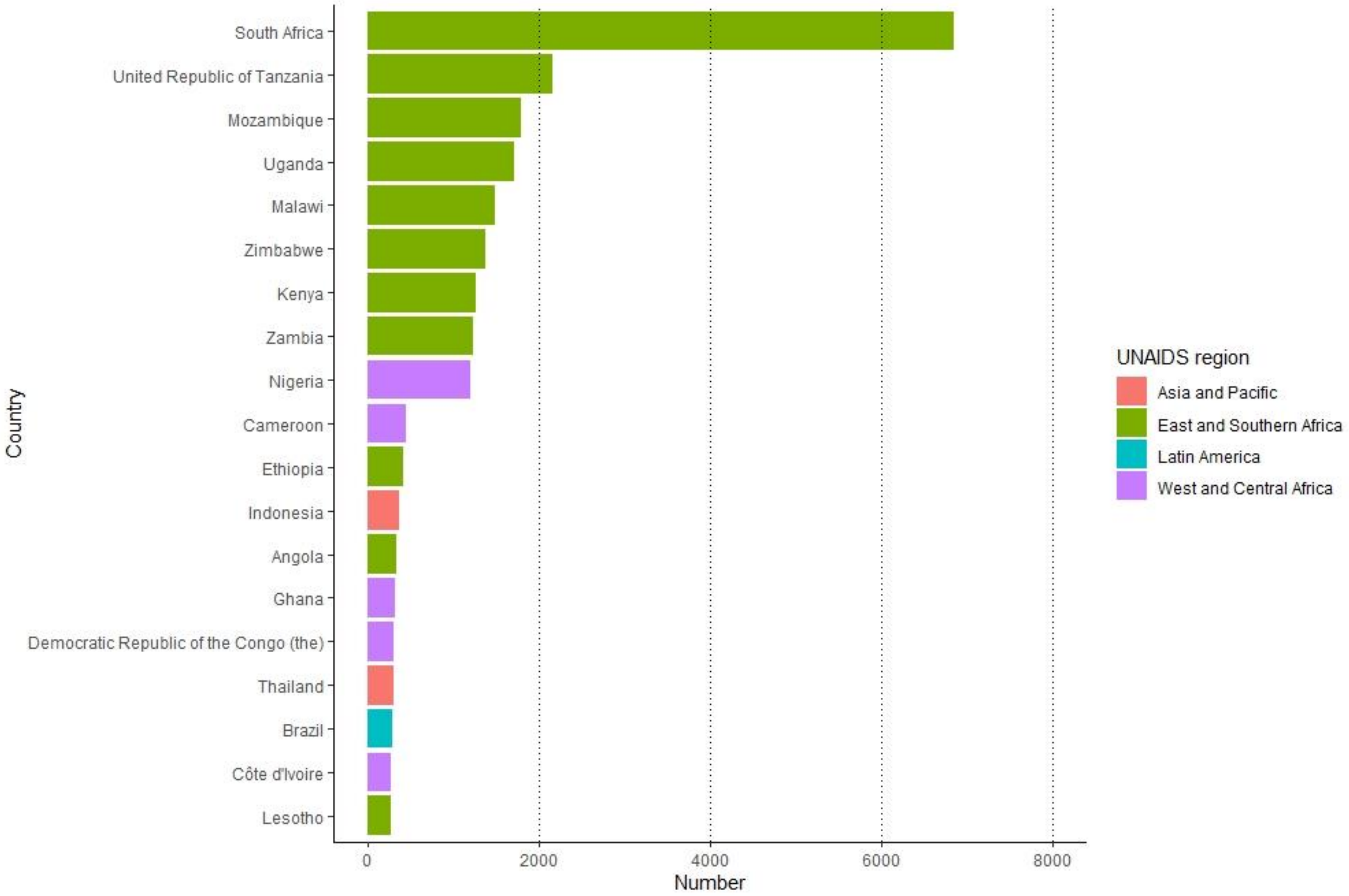


‡ India, China and Russia also have >250 cases, but their HIV estimates are suppressed.

Figure 16. Forest plots for analyses by risk measure and continent

| Subgroups | No. of Estimates | $I^2$ | Pooled risk ratios [95% CI] |
|---|---|---|---|
| ***Risk measure*** | | | |
| SIR | 20 | 94.5% | 6.79 [4.41-10.47] |
| RR | 2 | 0% | 8.43 [5.09-13.98] |
| OR | 9 | 47.5% | 4.17 [2.8-6.22] |
| ***Continent*** | | | |
| Africa | 11 | 51.2% | 3.7 [2.62-5.24] |
| Asia | 3 | 94.7% | 16.47 [3.48-77.97] |
| Europe | 7 | 67.6% | 5.01 [3.47-7.23] |
| Americas | 10 | 93.2% | 8.23 [4.46-15.21] |

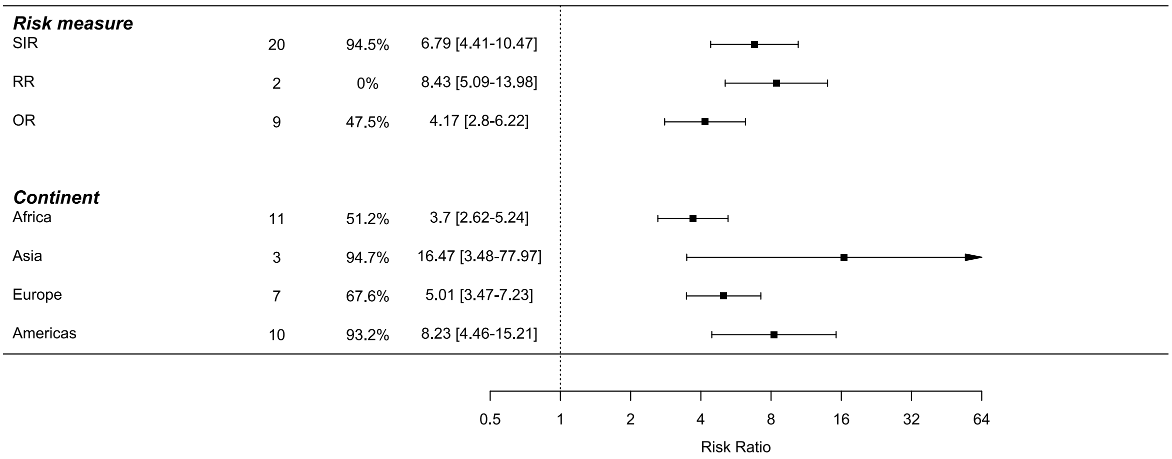

Table 7: Definition of WHO regions

| WHO region | Country |
|---|---|
| Africa (AFRO) | Algeria |
| | Angola |
| | Benin |
| | Botswana |
| | Burkina Faso |
| | Burundi |
| | Cabo Verde |
| | Cameroon |
| | Central African Republic (the) |
| | Chad |
| | Comoros (the) |
| | Congo |
| | Côte d'Ivoire |
| | Democratic Republic of the Congo (the) |
| | Equatorial Guinea |
| | Eritrea |
| | Eswatini |
| | Ethiopia |
| | Gabon |
| | Gambia |
| | Ghana |
| | Guinea |
| | Guinea-Bissau |
| | Kenya |
| | Lesotho |
| | Liberia |
| | Madagascar |
| | Malawi |
| | Mali |
| | Mauritania |
| | Mauritius |
| | Mozambique |
| | Namibia |
| | Niger |
| | Nigeria |
| | Rwanda |
| | Sao Tome and Principe |
| | Senegal |
| | Seychelles |
| | Sierra Leone |
| | South Africa |
| | South Sudan |
| | Togo |
| | Uganda |

| | |
|---|---|
| | United Republic of Tanzania<br>Zambia<br>Zimbabwe |
| America (PAHO) | Antigua and Barbuda<br>Argentina<br>Bahamas (the)<br>Barbados<br>Belize<br>Bolivia<br>Brazil<br>Canada<br>Chile<br>Colombia<br>Costa Rica<br>Cuba<br>Dominica<br>Dominican Republic (the)<br>Ecuador<br>El Salvador<br>Grenada<br>Guatemala<br>Guyana<br>Haiti<br>Honduras<br>Jamaica<br>Mexico<br>Nicaragua<br>Panama<br>Paraguay<br>Peru<br>Saint Kitts and Nevis<br>Saint Lucia<br>Saint Vincent and the Grenadines<br>Suriname<br>Trinidad and Tobago<br>United States of America<br>Uruguay<br>Venezuela |
| Eastern Mediterranean (EMRO) | Afghanistan<br>Bahrain<br>Djibouti<br>Egypt<br>Iran<br>Iraq<br>Jordan<br>Kuwait |

| | |
|---|---|
| | Lebanon |
| | Libya |
| | Morocco |
| | Occupied Palestinian Territory |
| | Oman |
| | Pakistan |
| | Qatar |
| | Saudi Arabia |
| | Somalia |
| | Sudan |
| | Syrian Arab Republic |
| | Tunisia |
| | United Arab Emirates |
| | Yemen |
| Europe (EURO) | Albania |
| | Andorra |
| | Armenia |
| | Austria |
| | Azerbaijan |
| | Belarus |
| | Belgium |
| | Bosnia and Herzegovina |
| | Bulgaria |
| | Croatia |
| | Cyprus |
| | Czech Republic |
| | Denmark |
| | Estonia |
| | Finland |
| | France |
| | Georgia |
| | Germany |
| | Greece |
| | Hungary |
| | Iceland |
| | Ireland |
| | Israel |
| | Italy |
| | Kazakhstan |
| | Kosovo |
| | Kyrgyzstan |
| | Latvia |
| | Liechtenstein |
| | Lithuania |
| | Luxembourg |
| | Malta |

| | |
|---|---|
| | Monaco |
| | Montenegro |
| | Netherlands |
| | North Macedonia |
| | Norway |
| | Poland |
| | Portugal |
| | Republic of Moldova |
| | Romania |
| | Russian Federation |
| | San Marino |
| | Serbia |
| | Slovakia |
| | Slovenia |
| | Spain |
| | Sweden |
| | Switzerland |
| | Tajikistan |
| | Turkey |
| | Turkmenistan |
| | Ukraine |
| | United Kingdom |
| | Uzbekistan |
| South-East Asia (SEARO) | Bangladesh |
| | Bhutan |
| | Democratic People's Republic of Korea (the) |
| | India |
| | Indonesia |
| | Maldives |
| | Myanmar |
| | Nepal |
| | Sri Lanka |
| | Thailand |
| | Timor-Leste |
| Western Pacific (WPRO) | Australia |
| | Brunei Darussalam |
| | Cambodia |
| | China |
| | Cook Islands |
| | Fiji |
| | Japan |
| | Kiribati |
| | Lao People's Democratic Republic |
| | Malaysia |
| | Marshall Islands |
| | Micronesia (Federated States of) |

| | |
|---|---|
| | Mongolia |
| | Nauru |
| | New Zealand |
| | Palau |
| | Papua New Guinea |
| | Philippines |
| | Republic of Korea |
| | Samoa |
| | Singapore |
| | Solomon Islands |
| | Tonga |
| | Tuvalu |
| | Vanuatu |
| | Viet Nam |

Table 8. Definition of UN Africa subregion

| WHO subregion | Country |
|---|---|
| Eastern Africa | Burundi |
| | Comoros |
| | Djibouti |
| | Eritrea |
| | Ethiopia |
| | Kenya |
| | Madagascar |
| | Malawi |
| | Mauritius |
| | Mayotte |
| | Mozambique |
| | Réunion |
| | Rwanda |
| | Seychelles |
| | Somalia |
| | South Sudan |
| | Uganda |
| | United Republic of Tanzania |
| | Zambia |
| | Zimbabwe |
| Central Africa | Angola |
| | Cameroon |
| | Central African Republic |
| | Chad |
| | Congo |
| | Democratic Republic of the Congo |
| | Equatorial Guinea |
| | Gabon |
| | Sao Tome and Principe |
| Northern Africa | Algeria |
| | Egypt |
| | Libya |
| | Morocco |
| | Sudan |
| | Tunisia |
| | Western Sahara |
| Southern Africa | Botswana |
| | Eswatini |
| | Lesotho |
| | Namibia |
| | South Africa |
| Western Africa | Benin |
| | Burkina Faso |
| | Cabo Verde |

| | |
|---|---|
| | Côte d’Ivoire |
| | Gambia |
| | Ghana |
| | Guinea |
| | Guinea-Bissau |
| | Liberia |
| | Mali |
| | Mauritania |
| | Niger |
| | Nigeria |
| | Senegal |
| | Sierra Leone |
| | Togo |

Table 9: Definition of UNAIDS regions

| UNAIDS region | Country |
|---|---|
| Asia and the Pacific (AP) | Afghanistan |
| | Australia |
| | Bangladesh |
| | Bhutan |
| | Brunei Darussalam |
| | Cambodia |
| | China |
| | Cook Islands |
| | Democratic People's Republic of Korea (the) |
| | Fiji |
| | India |
| | Indonesia |
| | Japan |
| | Kiribati |
| | Lao People's Democratic Republic |
| | Malaysia |
| | Maldives |
| | Marshall Islands |
| | Micronesia (Federated States of) |
| | Mongolia |
| | Myanmar |
| | Nauru |
| | Nepal |
| | New Zealand |
| | Niue |
| | Pakistan |
| | Palau |
| | Papua New Guinea |
| | Philippines |
| | Republic of Korea |
| | Samoa |
| | Singapore |
| | Solomon Islands |
| | Sri Lanka |
| | Thailand |
| | Timor-Leste |
| | Tonga |
| | Tuvalu |
| | Vanuatu |
| | Viet Nam |
| Caribbean (CAR) | Antigua and Barbuda |
| | Bahamas (the) |
| | Barbados |

| | |
|---|---|
| | Belize |
| | Cuba |
| | Dominica |
| | Dominican Republic (the) |
| | Grenada |
| | Guyana |
| | Haiti |
| | Jamaica |
| | Saint Kitts and Nevis |
| | Saint Lucia |
| | Saint Vincent and the Grenadines |
| | Suriname |
| | Trinidad and Tobago |
| East and Southern Africa (ESA) | Angola |
| | Botswana |
| | Comoros (the) |
| | Eritrea |
| | Eswatini |
| | Ethiopia |
| | Kenya |
| | Lesotho |
| | Madagascar |
| | Malawi |
| | Mauritius |
| | Mozambique |
| | Namibia |
| | Rwanda |
| | Seychelles |
| | South Africa |
| | South Sudan |
| | Uganda |
| | United Republic of Tanzania |
| | Zambia |
| | Zimbabwe |
| Eastern Europe and Central Asia (EECA) | Albania |
| | Armenia |
| | Azerbaijan |
| | Belarus |
| | Bosnia and Herzegovina |
| | Georgia |
| | Kazakhstan |
| | Kyrgyzstan |
| | Montenegro |
| | North Macedonia |
| | Republic of Moldova |
| | Russian Federation |

| | |
|---|---|
| | Tajikistan |
| | Turkmenistan |
| | Ukraine |
| | Uzbekistan |
| Latin America (LA) | Argentina |
| | Bolivia |
| | Brazil |
| | Chile |
| | Colombia |
| | Costa Rica |
| | Ecuador |
| | El Salvador |
| | Guatemala |
| | Honduras |
| | Mexico |
| | Nicaragua |
| | Panama |
| | Paraguay |
| | Peru |
| | Uruguay |
| | Venezuela |
| Middle East and North Africa (MENA) | Algeria |
| | Bahrain |
| | Djibouti |
| | Egypt |
| | Iran |
| | Iraq |
| | Jordan |
| | Kuwait |
| | Lebanon |
| | Libya |
| | Morocco |
| | Occupied Palestinian Territory |
| | Oman |
| | Qatar |
| | Saudi Arabia |
| | Somalia |
| | Sudan |
| | Syrian Arab Republic |
| | Tunisia |
| | United Arab Emirates |
| | Yemen |
| West and Central Africa (WCA) | Benin |
| | Burkina Faso |
| | Burundi |
| | Cabo Verde |

| | |
|---|---|
| | Cameroon |
| | Central African Republic (the) |
| | Chad |
| | Congo |
| | Côte d'Ivoire |
| | Democratic Republic of the Congo (the) |
| | Equatorial Guinea |
| | Gabon |
| | Gambia |
| | Ghana |
| | Guinea |
| | Guinea-Bissau |
| | Liberia |
| | Mali |
| | Mauritania |
| | Niger |
| | Nigeria |
| | Sao Tome and Principe |
| | Senegal |
| | Sierra Leone |
| | Togo |
| Western and Central Europe and North America (WCENA) | Andorra |
| | Austria |
| | Belgium |
| | Bulgaria |
| | Canada |
| | Croatia |
| | Cyprus |
| | Czech Republic |
| | Denmark |
| | Estonia |
| | Finland |
| | France |
| | Germany |
| | Greece |
| | Hungary |
| | Iceland |
| | Ireland |
| | Israel |
| | Italy |
| | Kosovo |
| | Latvia |
| | Liechtenstein |
| | Lithuania |
| | Luxembourg |
| | Malta |

| | |
|---|---|
| | Monaco |
| | Netherlands |
| | Norway |
| | Poland |
| | Portugal |
| | Romania |
| | San Marino |
| | Serbia |
| | Slovakia |
| | Slovenia |
| | Spain |
| | Sweden |
| | Switzerland |
| | Turkey |
| | United Kingdom |
| | United States of America |

Table 10: Definition of income level by the World Bank

| Income group | Country | | |
|---|---|---|---|
| High income | Andorra | Germany | Oman |
| | Antigua and Barbuda | Gibraltar | Palau |
| | Argentina | Greece | Panama |
| | Aruba | Greenland | Poland |
| | Australia | Guam | Portugal |
| | Austria | Hong Kong SAR | Puerto Rico |
| | Bahamas | Hungary | Qatar |
| | Bahrain | Iceland | San Marino |
| | Barbados | Ireland | Saudi Arabia |
| | Belgium | Isle of Man | Seychelles |
| | Bermuda | Israel | Singapore |
| | British Virgin Islands | Italy | Sint Maarten (Dutch part) |
| | Brunei Darussalam | Japan | Slovak Republic |
| | Canada | Korea | Slovenia |
| | Cayman Islands | Kuwait | Spain |
| | Channel Islands | Latvia | St. Kitts and Nevis |
| | Chile | Liechtenstein | St. Martin (French part) |
| | Croatia | Lithuania | Sweden |
| | Curaçao | Luxembourg | Switzerland |
| | Cyprus | Macao SAR | Taiwan |
| | Czech Republic | Malta | Trinidad and Tobago |
| | Denmark | Monaco | Turks and Caicos Islands |
| | Estonia | Netherlands | United Arab Emirates |
| | Faroe Islands | New Caledonia | United Kingdom |
| | Finland | New Zealand | United States |
| | France | Northern Mariana Islands | Uruguay |
| | French Polynesia | Norway | Virgin Islands (U.S.) |

| | | | |
|---|---|---|---|
| Upper middle income | Albania | Gabon | Nauru |
| | Algeria | Grenada | Paraguay |
| | American Samoa | Guatemala | Peru |
| | Armenia | Guyana | Romania |
| | Azerbaijan | Iran | Russian Federation |
| | Belarus | Iraq | Samoa |
| | Belize | Jamaica | Serbia |
| | Bosnia and Herzegovina | Jordan | South Africa |
| | Botswana | Kazakhstan | St. Lucia |
| | Brazil | Lebanon | St. Vincent and the Grenadines |
| | Bulgaria | Libya | Suriname |
| | China | Macedonia | Thailand |
| | Colombia | Malaysia | Tonga |
| | Costa Rica | Maldives | Turkey |
| | Cuba | Marshall Islands | Turkmenistan |
| | Dominica | Mauritius | Tuvalu |
| | Dominican Republic | Mexico | Venezuela |
| | Ecuador | Montenegro | |
| | Equatorial Guinea | Namibia | |
| Lower middle income | Angola | Indonesia | Papua New Guinea |
| | Bangladesh | Kenya | Philippines |
| | Bhutan | Kiribati | São Tomé and Principe |
| | Bolivia | Kosovo | Solomon Islands |
| | Cabo Verde | Kyrgyz Republic | Sri Lanka |
| | Cambodia | Lao PDR | Sudan |
| | Cameroon | Lesotho | Swaziland |
| | Congo | Mauritania | Timor-Leste |
| | Côte d'Ivoire | Micronesia | Tunisia |
| | Djibouti | Moldova | Ukraine |
| | Egypt | Mongolia | Uzbekistan |

| | | | |
|---|---|---|---|
| | El Salvador | Morocco | Vanuatu |
| | Georgia | Myanmar | Vietnam |
| | Ghana | Nicaragua | West Bank and Gaza |
| | Honduras | Nigeria | Zambia |
| | India | Pakistan | |
| Low income | Benin | Guinea-Bissau | Senegal |
| | Burkina Faso | Haiti | Sierra Leone |
| | Burundi | Korea | Somalia |
| | Central African Republic | Liberia | South Sudan |
| | Chad | Madagascar | Syrian Arab Republic |
| | Comoros | Malawi | Tajikistan |
| | Congo | Mali | Tanzania |
| | Eritrea | Mozambique | Togo |
| | Ethiopia | Nepal | Uganda |
| | Gambia | Niger | Yemen |
| | Guinea | Rwanda | Zimbabwe |

Table 11. Countries and territories with no HIV estimates, for which no estimates of HIV-associated cervical cancer burden were calculated in this study

| **Country** | **Number of new cervical cancer cases 2018** |
|---|---|
| France, Guadeloupe | 39 [26–58] |
| France, La Réunion | 70 [53–93] |
| France, Martinique | 32 [22–48] |
| France, New Caledonia | 30 [19–48] |
| French Guyana | 29 [16–52] |
| French Polynesia | 17 [9–31] |
| Gaza Strip and West Bank | 38 [18–80] |
| Guam | 18 [14–23] |
| Iraq | 244 [205–291] |
| Puerto Rico | 262 [229–300] |
| Saint Lucia | 15 [7–31] |
| Samoa | 10 [5–20] |
| Sao Tome and Principe | 16 [6–42] |
| Solomon Islands | 55 [44–68] |
| Vanuatu | 21 [12–36] |

## References


1. Abraham AG, D'Souza G, Jing Y, et al. Invasive cervical cancer risk among HIV-infected women: a North American multicohort collaboration prospective study. *J Acquir Immune Defic Syndr* 2013; **62**(4): 405-13.
2. Adjorlolo-Johnson G, Unger ER, Boni-Ouattara E, et al. Assessing the relationship between HIV infection and cervical cancer in Cote d'Ivoire: a case-control study. *BMC Infect Dis* 2010; **10**: 242.
3. Akarolo-Anthony SN, Maso Ld, Igbinoba F, Mbulaiteye SM, Adebamowo CA. Cancer burden among HIV-positive persons in Nigeria: preliminary findings from the Nigerian AIDS-cancer match study. *Infect Agents Cancer* 2014; **9**(1).
4. Allardice GM, Hole DJ, Brewster DH, Boyd J, Goldberg DJ. Incidence of malignant neoplasms among HIV-infected persons in Scotland. *Br J Cancer* 2003; **89**(3): 505-7.
5. Bedimo RJ, McGinnis KA, Dunlap M, Rodriguez-Barradas MC, Justice AC. Incidence of Non-AIDS-Defining Malignancies in HIV-Infected Versus Noninfected Patients in the HAART Era: Impact of Immunosuppression. *J Acquir Immune Defic Syndr* 2009; **52**(2): 203-8.
6. Carlander C, Wagner P, Svedhem V, et al. Impact of immunosuppression and region of birth on risk of cervical intraepithelial neoplasia among migrants living with HIV in Sweden. *Int J Cancer* 2016; **139**(7): 1471-9.
7. Castilho JL, Luz PM, Shepherd BE, et al. HIV and cancer: A comparative retrospective study of Brazilian and U.S. clinical cohorts. *Infect Agents Cancer* 2015; **10**(1).
8. Chaturvedi AK, Madeleine MM, Biggar RJ, Engels EA. Risk of human papillomavirus-associated cancers among persons with AIDS. *J Natl Cancer Inst* 2009; **101**(16): 1120-30.
9. Chaturvedi AK, Mbulaiteye SM, Engels EA. Underestimation of relative risks by standardized incidence ratios for AIDS-related cancers. *Ann Epidemiol* 2008; **18**(3): 230-4.
10. Chen CH, Chung CY, Wang LH, Lin C, Lin HL, Lin HC. Risk of cancer among HIV-infected patients from a population-based nested case-control study: implications for cancer prevention. *BMC Cancer* 2015; **15**: 133.
11. Chen YC, Li CY, Liu HY, Lee NY, Ko WC, Ko NY. Effect of antiretroviral therapy on the incidence of cervical neoplasia among HIV-infected women: a population-based cohort study in Taiwan. *AIDS* 2014; **28**(5): 709-15.
12. Clifford GM, Polesel J, Rickenbach M, et al. Cancer risk in the Swiss HIV Cohort Study: associations with immunodeficiency, smoking, and highly active antiretroviral therapy. *J Natl Cancer Inst* 2005; **97**(6): 425-32.
13. Cooksley CD, Hwang LY, Waller DK, Ford CE. HIV-related malignancies: community-based study using linkage of cancer registry and HIV registry data. *Int J STD AIDS* 1999; **10**(12): 795-802.
14. Dal Maso L, Polesel J, Serraino D, et al. Pattern of cancer risk in persons with AIDS in Italy in the HAART era. *Br J Cancer* 2009; **100**(5): 840-7.
15. Engels EA, Biggar RJ, Hall HI, et al. Cancer risk in people infected with human immunodeficiency virus in the United States. *Int J Cancer* 2008; **123**(1): 187-94.
16. Engels EA, Pfeiffer RM, Goedert JJ, et al. Trends in cancer risk among people with AIDS in the United States 1980-2002. *AIDS* 2006; **20**(12): 1645-54.
17. Franceschi S, Dal Maso L, Arniani S, et al. Risk of cancer other than Kaposi's sarcoma and non-Hodgkin's lymphoma in persons with AIDS in Italy. Cancer and AIDS Registry Linkage Study. *Br J Cancer* 1998; **78**(7): 966-70.

18. Frisch M, Biggar RJ, Engels EA, Goedert JJ. Association of cancer with AIDS-related immunosuppression in adults. *Jama* 2001; **285**(13): 1736-45.
19. Galceran J, Marcos-Gragera R, Soler M, et al. Cancer incidence in AIDS patients in Catalonia, Spain. *Eur J Cancer* 2007; **43**(6): 1085-91.
20. Gallagher B, Wang Z, Schymura MJ, Kahn A, Fordyce EJ. Cancer incidence in New York State acquired immunodeficiency syndrome patients. *Am J Epidemiol* 2001; **154**(6): 544-56.
21. Godbole SV, Nandy K, Gauniyal M, et al. HIV and cancer registry linkage identifies a substantial burden of cancers in persons with HIV in India. *Medicine (Baltimore)* 2016; **95**(37): e4850.
22. Grabar S, Hleyhel M, Belot A, et al. Invasive cervical cancer in HIV-infected women: risk and survival relative to those of the general population in France. Results from the French Hospital Database on HIV (FHDH)–Agence Nationale de Recherches sur le SIDA et les Hépatites Virales (ANRS) CO4 cohort study. *HIV Med* 2019; **20**(3): 222-9.
23. Hawes SE, Critchlow CW, Sow PS, et al. Incident high-grade squamous intraepithelial lesions in Senegalese women with and without human immunodeficiency virus type 1 (HIV-1) and HIV-2. *J Natl Cancer Inst* 2006; **98**(2): 100-9.
24. Helleberg M, Gerstoft J, Afzal S, et al. Risk of cancer among HIV-infected individuals compared to the background population: Impact of smoking and HIV. *AIDS* 2014; **28**(10): 1499-508.
25. Hernandez-Ramirez RU, Shiels MS, Dubrow R, Engels EA. Cancer risk among HIV-infected people in the us during the modern treatment era. *Topics in Antiviral Medicine* 2017; **25**(1): 249s.
26. Hessol NA, Seaberg EC, Preston-Martin S, et al. Cancer risk among participants in the women's interagency HIV study. *J Acquir Immune Defic Syndr* 2004; **36**(4): 978-85.
27. Hleyhel M, Belot A, Bouvier AM, et al. Risk of AIDS-defining cancers among HIV-1-infected patients in France between 1992 and 2009: results from the FHDH-ANRS CO4 cohort. *Clin Infect Dis* 2013; **57**(11): 1638-47.
28. Holmes RS, Hawes SE, Toure P, et al. HIV infection as a risk factor for cervical cancer and cervical intraepithelial neoplasia in Senegal. *Cancer Epidemiol Biomarkers Prev* 2009; **18**(9): 2442-6.
29. Jaquet A, Odutola M, Ekouevi DK, et al. Cancer and HIV infection in referral hospitals from four West African countries. *Cancer Epidemiol* 2015; **39**(6): 1060-5.
30. Kadhel P, Multigner L, Bardinet F, Goerger-Sow M, Janky E. Cervical intraepithelial neoplasia and invasive cancer risks in women infected with HIV in the French West Indies. *HIV Med* 2012; **13**(1): 79-82.
31. Kahesa C, Mwaiselage J, Wabinga HR, Ngoma T, Kalyango JN, Karamagi CA. Association between invasive cancer of the cervix and HIV-1 infection in Tanzania: the need for dual screening. *BMC Public Health* 2008; **8**: 262.
32. Keller MJ, Burk RD, Massad LS, et al. Cervical precancer risk in HIV-infected women who test positive for oncogenic human Papillomavirus despite a normal pap test. *Clin Infect Dis* 2015; **61**(10): 1573-81.
33. Khuakoonratt N, Tangjitgamol S, Manusirivithaya S, et al. Prevalence of high grade squamous intraepithelial lesion (HSIL) and invasive cervical cancer in patients with low grade squamous intraepithelial lesion (LSIL) at cervical pap smear. *Asian Pac J Cancer Prev* 2008; **9**(2): 253-7.
34. Mahale P, Engels EA, Coghill AE, Kahn AR, Shiels MS. Cancer risk in older persons living with Human Immunodeficiency Virus infection in the United States. *Clinical Infectious Diseases* 2018; **67**(1): 50-7.

35. Massad LS, Evans CT, D'Souza G, et al. High-grade cervical disease in adolescents with HIV. *J Low Genit Tract Dis* 2008; **12**(3): 199-203.
36. Massad LS, Hessol NA, Darragh TM, et al. Cervical cancer incidence after up to 20 years of observation among women with HIV. *Int J Cancer* 2017; **141**(8): 1561-5.
37. Mayans MV, Maguire A, Miret M, Casabona J. Disproportionate high incidence of invasive cervical cancer as an AIDS-indicative disease among young women in Catalonia, Spain. *Sex Transm Dis* 1999; **26**(9): 500-3.
38. Mayor AM, Santiago-Rodriguez EJ, Rios-Olivares E, Tortolero-Luna G, Hunter-Mellado RF. Malignancies trends in a hispanic cohort of HIV persons in puerto rico before and after cART. *International Journal of Cancer Research* 2016; **12**(2): 92-100.
39. Mbulaiteye SM, Katabira ET, Wabinga H, et al. Spectrum of cancers among HIV-infected persons in Africa: the Uganda AIDS-Cancer Registry Match Study. *Int J Cancer* 2006; **118**(4): 985-90.
40. Moscicki AB, Ellenberg JH, Crowley-Nowick P, Darragh TM, Xu J, Fahrat S. Risk of high-grade squamous intraepithelial lesion in HIV-infected adolescents. *J Infect Dis* 2004; **190**(8): 1413-21.
41. Mpunga T, Znaor A, Uwizeye FR, et al. A case-control study of HIV infection and cancer in the era of antiretroviral therapy in Rwanda. *Int J Cancer* 2018; **143**(6): 1348-55.
42. Newnham A, Harris J, Evans HS, Evans BG, Moller H. The risk of cancer in HIV-infected people in southeast England: a cohort study. *Br J Cancer* 2005; **92**(1): 194-200.
43. Newton R, Ziegler J, Beral V, et al. A case-control study of human immunodeficiency virus infection and cancer in adults and children residing in Kampala, Uganda. *Int J Cancer* 2001; **92**(5): 622-7.
44. Phelps RM, Smith DK, Heilig CM, et al. Cancer incidence in women with or at risk for HIV. *Int J Cancer* 2001; **94**(5): 753-7.
45. Salters KA, Cescon A, Zhang W, et al. Cancer incidence among HIV-positive women in British Columbia, Canada: Heightened risk of virus-related malignancies. *HIV Med* 2016; **17**(3): 188-95.
46. Sekirime WK, Gray R. HIV infection among Uganda women with cervical cancer: a retrospective study. *Gynecol Obstet Invest* 2007; **63**(4): 222-8.
47. Selik RM, Rabkin CS. Cancer death rates associated with human immunodeficiency virus infection in the United States. *J Natl Cancer Inst* 1998; **90**(17): 1300-2.
48. Serraino D, Carrieri P, Pradier C, et al. Risk of invasive cervical cancer among women with, or at risk for, HIV infection. *Int J Cancer* 1999; **82**(3): 334-7.
49. Silverberg MJ, Chao C, Leyden WA, et al. HIV infection and the risk of cancers with and without a known infectious cause. *AIDS* 2009; **23**(17): 2337-45.
50. Silverberg MJ, Leyden WA, Chi A, et al. Human immunodeficiency Virus (HIV)- and non-HIV-associated immunosuppression and risk of cervical neoplasia. *Obstet Gynecol* 2018; **131**(1): 47-55.
51. Sitas F, Bezwoda WR, Levin V, et al. Association between human immunodeficiency virus type 1 infection and cancer in the black population of Johannesburg and Soweto, South Africa. *Br J Cancer* 1997; **75**(11): 1704-7.
52. Stein L, Urban MI, O'Connell D, et al. The spectrum of human immunodeficiency virus-associated cancers in a South African black population: results from a case-control study, 1995-2004. *Int J Cancer* 2008; **122**(10): 2260-5.
53. Tanaka LF, Latorre M, Gutierrez EB, et al. Risk for cancer among people living with AIDS, 1997-2012: the Sao Paulo AIDS-cancer linkage study. *Eur J Cancer Prev* 2018; **27**(4): 411-7.
54. Tanon A, Jaquet A, Ekouevi DK, et al. The spectrum of cancers in West Africa: associations with human immunodeficiency virus. *PLoS One* 2012; **7**(10): e48108.

55. Thorsteinsson K, Ladelund S, Jensen-Fangel S, et al. Incidence of cervical dysplasia and cervical cancer in women living with HIV in Denmark: comparison with the general population. *HIV Med* 2016; **17**(1): 7-17.
56. Uribe Parra D, Pulido D, Lopes G, et al. Cancer incidence in patients diagnosed with Acquired Immunodeficiency Syndrome (AIDS) in Colombia. *Journal of AIDS and Clinical Research* 2017; **8**(5): 692-.
57. Vogel M, Friedrich O, Luchters G, et al. Cancer risk in HIV-infected individuals on HAART is largely attributed to oncogenic infections and state of immunocompetence. *Eur J Med Res* 2011; **16**(3): 101-7.
58. Whitham HK, Hawes SE, Chu H, et al. A Comparison of the Natural History of HPV Infection and Cervical Abnormalities among HIV-Positive and HIV-Negative Women in Senegal, Africa. *Cancer Epidemiol Biomarkers Prev* 2017; **26**(6): 886-94.
59. Zhang YX, Gui XE, Zhong YH, Rong YP, Yan YJ. Cancer in cohort of HIV-infected population: prevalence and clinical characteristics. *J Cancer Res Clin Oncol* 2011; **137**(4): 609-14.
60. Zohar M, Micha B. Cancer incidence in people Living with HIV/AIDS in Israel, 1981-2010. *AIDS Research and Human Retroviruses* 2015; **31**(9): 873-81.
61. Stroup DF, Berlin JA, Morton SC, et al. Meta-analysis of observational studies in epidemiology: a proposal for reporting. Meta-analysis Of Observational Studies in Epidemiology (MOOSE) group. *Jama* 2000; **283**(15): 2008-12.
62. Moher D, Liberati A, Tetzlaff J, Altman DG. Preferred reporting items for systematic reviews and meta-analyses: the PRISMA statement. *PLoS Med* 2009; **6**(7): e1000097.
